# Multiomic profiling of glioblastoma metabolic lesions reveals complex intratumoral genomic evolution and dipeptidase-1-driven vascular proliferation

**DOI:** 10.1093/neuonc/noaf071

**Published:** 2025-05-04

**Authors:** Atul Anand, Jeanette Krogh Petersen, Lars van Brakel Andersen, Mark Burton, Clara Rosa Levina Oudenaarden, Martin Jakob Larsen, Philip Ahle Erichsen, Christian Bonde Pedersen, Frantz Rom Poulsen, Peter Grupe, Torben A Kruse, Mads Thomassen, Bjarne Winther Kristensen

**Affiliations:** Department of Pathology, The Bartholin Institute, Rigshospitalet, Copenhagen University Hospital, Copenhagen, Denmark; Department of Clinical Medicine and Biotech Research and Innovation Center (BRIC), University of Copenhagen, Copenhagen, Denmark; Department of Clinical Research, University of Southern Denmark, Odense, Denmark; Department of Pathology, Odense University Hospital, Odense, Denmark; Department of Pathology, Odense University Hospital, Odense, Denmark; Clinical Genome Center, Department of Clinical Research, University of Southern Denmark, Odense, Denmark; Department of Clinical Genetics, Odense University Hospital, Odense, Denmark; Clinical Genome Center, Department of Clinical Research, University of Southern Denmark, Odense, Denmark; Department of Clinical Genetics, Odense University Hospital, Odense, Denmark; Department of Pathology, The Bartholin Institute, Rigshospitalet, Copenhagen University Hospital, Copenhagen, Denmark; Department of Clinical Medicine and Biotech Research and Innovation Center (BRIC), University of Copenhagen, Copenhagen, Denmark; Clinical Genome Center, Department of Clinical Research, University of Southern Denmark, Odense, Denmark; Department of Clinical Genetics, Odense University Hospital, Odense, Denmark; Department of Pathology, The Bartholin Institute, Rigshospitalet, Copenhagen University Hospital, Copenhagen, Denmark; Department of Clinical Medicine and Biotech Research and Innovation Center (BRIC), University of Copenhagen, Copenhagen, Denmark; BRIDGE, Brain Research - Inter Disciplinary Guided Excellence, Odense University Hospital and University of Southern Denmark, Odense, Denmark; Department of Neurosurgery, Odense University Hospital, Odense, Denmark; Department of Clinical Research, University of Southern Denmark, Odense, Denmark; BRIDGE, Brain Research - Inter Disciplinary Guided Excellence, Odense University Hospital and University of Southern Denmark, Odense, Denmark; Department of Neurosurgery, Odense University Hospital, Odense, Denmark; Department of Clinical Research, University of Southern Denmark, Odense, Denmark; Department of Nuclear Medicine, Odense University Hospital, Odense, Denmark; Clinical Genome Center, Department of Clinical Research, University of Southern Denmark, Odense, Denmark; Department of Clinical Genetics, Odense University Hospital, Odense, Denmark; Clinical Genome Center, Department of Clinical Research, University of Southern Denmark, Odense, Denmark; Department of Clinical Genetics, Odense University Hospital, Odense, Denmark; Department of Pathology, The Bartholin Institute, Rigshospitalet, Copenhagen University Hospital, Copenhagen, Denmark; Department of Clinical Medicine and Biotech Research and Innovation Center (BRIC), University of Copenhagen, Copenhagen, Denmark; Department of Clinical Research, University of Southern Denmark, Odense, Denmark; Department of Pathology, Odense University Hospital, Odense, Denmark

**Keywords:** dipeptidase 1, hypermetabolism, mutational burden, tumor evolution, vascular tip

## Abstract

**Background:**

Glioblastoma undergoes a complex and dynamic evolution involving genetic and epigenetic changes. Understanding the mechanisms underlying this evolution is vital for the development of efficient therapeutic strategies. Although treatment resistance is associated with intratumoral heterogeneity in glioblastoma, it remains uncertain whether hypometabolic and hypermetabolic lesions observed through clinical positron emission tomography (PET) imaging are influenced by spatial intratumoral genomic evolution.

**Methods:**

In this study, we precisely isolated autologous hypometabolic and hypermetabolic lesions from glioblastoma using advanced neurosurgical and brain tumor imaging technologies, followed by comprehensive whole-genome, exome, transcriptome, and imaging analyses.

**Results:**

Our findings unveil that hypermetabolic lesions, originating from hypometabolic lesions, exhibit strategic focal amplifications and deletions, and heightened APOBEC3 activity. Furthermore, we identify dipeptidase 1 as a novel vascular endothelial tip marker for hypermetabolic lesions in glioblastoma, facilitating angiogenesis and tumor metabolism by regulating transporter activities.

**Conclusions:**

Hypermetabolic lesions are associated with a higher frequency of genomic abnormalities and dipeptidase 1 emerges as a novel diagnostic and prognostic vascular marker for hypermetabolic lesions. This study underscores a spatial genomic evolution with diagnostic implications and elucidates challenges and opportunities crucial for the development of novel therapeutic strategies.

## Graphical Abstract

**Graphical Abstract:**
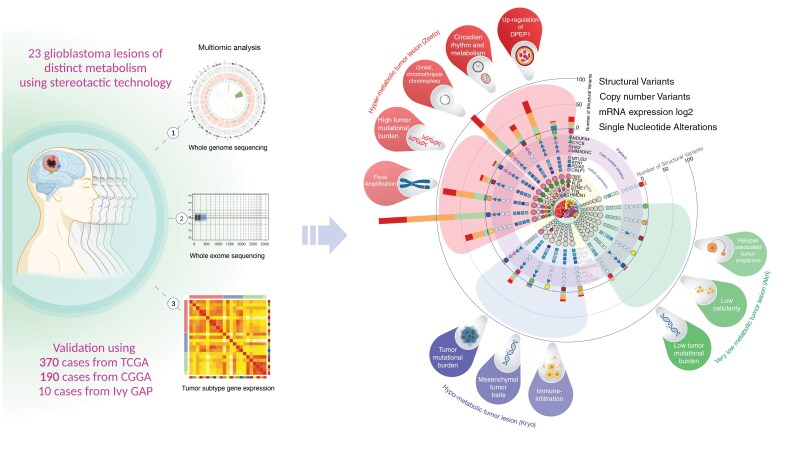

**Key Points**

1. Hypermetabolic lesions evolve and transform from hypometabolic lesions through harboring more genetic changes.
2. Hypermetabolic lesions exhibit greater spatial and genomic diversity compared to hypometabolic lesions.
3. Dipeptidase 1 is a novel diagnostic and prognostic vascular tip marker for hypermetabolic lesions.

**Importance of the Study**

Glioblastoma is a multifaceted disease that remains challenging to treat, with limited insights into the metabolic gradients observed in imaging and the underlying genomic evolution. This study is the first to investigate the molecular basis of hypermetabolic tumor lesions in glioblastoma using precise 3-dimensional biopsy isolation, whole genome–exome sequencing, and mRNA sequencing. These findings have diagnostic implications, reveal dipeptidase1 as a novel vascular tip marker, and illuminate the challenges faced by precision therapeutics in the treatment of glioblastoma.

Glioblastoma (GBM) is the most common and aggressive brain tumor, with about 80, 000 new cases per year worldwide and a median survival of about 15 months.^1,2^ Besides surgery, GBM treatment consists of radiation and chemotherapy, but most tumors inevitably recur.^3^ GBMs are characterized by extensive heterogeneity at both histological and molecular levels, representing the evolution of tumor aggressiveness and posing an obstacle to designing effective therapies.^4–8^ Although spatially random or bulk tumor biopsies have been used in analyses to understand the complexity of glioblastoma, they have contributed to our understanding of general GBM subtypes and their developmental trajectories to some extent.^9–11^ However, the link between intratumoral spatial metabolic heterogeneity and genomic evolution has not yet been fully explored.

In the clinical setting, magnetic resonance imaging (MRI) and positron emission tomography (PET) provide valuable information regarding tumor metabolism and location.^12–14^ However, it is unclear whether metabolically active tumor areas are genomically the most aggressive and whether this is associated with therapy resistance. We used MRI co-registered with ^11^C-methionine PET (^11^C-MET-PET) and ^18^F-fluorodeoxyglucose PET (^18^F-FDG-PET) brain imaging to identify tumor areas with different metabolic activities, followed by precise extraction of biopsies from these lesions using a state-of-the-art neurosurgical stereotactic approach, guided by 3-dimensional coordinates derived from MRI and PET imaging. The categorization of tumor areas in each patient was based on MRI, ^11^C-MET-PET, and ^18^F-FDG-PET. Regions with low to medium MRI and PET intensity were classified as ‘‘hypometabolic lesions,” symbolized by the Greek word ‘‘kryo” meaning cold. In contrast, regions with high MRI intensity and PET were classified as ‘‘hypermetabolic lesions,” represented by the Greek word ‘‘zesto” meaning hot. Finally, tissue regions located outside the enhancing border of the tumor observed on T1-MRI, with no PET signal intensity, were classified as “metabolically inactive” or referred to as “edge” (Greek: “akri”). These areas were isolated from lesions beyond the enhancing tumor boundary seen on T1-MRI.

The use of PET-guided stereotactic brain biopsies to obtain glioblastoma specimens is highly specialized and often not feasible globally. This study is the first to perform a comprehensive multi-omic analysis on predefined, multi-regional metabolic tumor lesions in glioblastoma patients. The analysis utilized advanced stereotactic biopsy techniques for optimal control. We examined genomic alterations in glioblastoma, including kryo, zesto, and akri samples, through exome, genome, and transcriptome sequencing of tissue and blood samples. This identified key copy number variations, single nucleotide variants, gene fusions, and somatic variants, along with RNA sequencing for functional analysis. By analyzing multisectoral paired biopsies, we explored intratumoral evolution and alterations, identifying molecular changes that impact glioblastoma biology, heterogeneity, and therapeutic resistance.

## Materials and Methods

### Glioblastoma Patients and Clinical Annotation

All patients in this study were histologically diagnosed with glioblastoma. For multi-omics analysis, only those with sufficient tumor tissue, normal tissue (400 mg), and blood specimens were included. In total, 23 fresh frozen glioblastoma patient tissues and 6 blood samples were collected at the Department of Neurosurgery, Odense University Hospital, Denmark. Tissues were collected during surgical resection, snap-frozen in liquid nitrogen for 45 minutes, and stored at −80 °C. DNA and RNA were extracted using the AllPrep DNA/RNA Kit (Qiagen#80284).

### Ethics Statement

This research complies with all ethical regulations. The use of stereotactic biopsy followed protocols from the Department of Neurosurgery, Odense University Hospital, Denmark. The study was approved by the Regions Scientific Ethical Committee of Southern Denmark (S-20140214). Patients were fully informed about the procedure and analysis, and written consent was obtained from each participant.

### MRI and PET Imaging

Patients with histologically confirmed glioblastoma, aged over 48, were scheduled for surgery the day after undergoing FDG-PET, MET-PET, and MRI. Conventional MRI was performed routinely, while ^18^F-FDG PET and ^11^C-MET PET were conducted separately on PET/CT scanners at Odense University Hospital’s Radiology Department. MRI (T1 with contrast enhancement) data was transferred to the Medtronic Stealth Neuro Navigation System.^15^

### Stereotactic Biopsy Isolation

To plan lesions from preoperatively defined areas in the tumor and its vicinity, MRI was co-registered with ^11^C-MET-PET and ^18^F-FDG PET. Areas of interest were predefined, and craniotomy was performed, ensuring the dura remained intact. Neuronavigational inaccuracy <1 mm was acceptable. To avoid brain shift, 3D stereotactic image-guided needle lesions were harvested before dural opening using the Medtronic^R^ vertex biopsy system. Regions with low uptake of ^18^F-FDG and ^11^C-MET were classified as metabolic kryo, high uptake as Zestos, and no uptake as akri in 6 glioblastoma patients. In one patient, a biopsy specimen surrounding the main tumor mass, called a tumor satellite, was isolated. The GE AW Server platform (Dexus) software analyzed, visualized, and co-registered the FDG-PET/MET-PET/MRI scans. Normal surgical time was 90–120 minutes, with the stereotactic biopsy procedure adding 60 minutes.

### Hematoxylin and Eosin, DPEP1, CD34 Staining

Biopsies were formaldehyde-fixed, paraffin-embedded, and cut into 3 µm sections. Hematoxylin and Eosin staining visualized cellularity. For immunohistochemistry, Dipeptidase 1 antibody (Sigma #HPA012783) or CD34 (QBEND/10) was diluted 1:100, incubated for 2 hours, followed by washing and secondary antibody application. Detection used a chromogen substrate, with optional hematoxylin or DAPI counterstaining. Slides were dehydrated, mounted, scanned with a NanoZoomer 2.0-HT, and analyzed using Visiopharm software.

### Aortic Ring Assay

Aortic rings were isolated from 8-week-old C57BL/6 mice, cleaned, and sectioned as described in Baker et al. (2012). The rings were plated in Geltrex-coated 48-well plates, serum-starved overnight, and treated every 2 days for 7 days with dimethyl sulfoxide (DMSO) or cilastatin. Sprouting was assessed by brightfield microscopy, and sprout number and area were quantified using the Celigo cytometer.

### Whole Genome Sequencing

We used 200 ng of DNA to prepare the library. For 25X coverage, whole-genome libraries underwent 150 bp paired-end sequencing on the Illumina NovaSeq 6000. FASTQ files were preprocessed per GATK’s best practices to obtain BAM files. BRASS identified structural variants, and TitanCNA identified copy number variants.

### Public Datasets Analysis

Normalized gene expression, patient survival, gene correlation, and mutational analyses in LGG and GBM were performed on data from TCGA, Chinese Glioma Genome Atlas (CGGA), Ivy GAP, and GEPIA20, obtained via GlioVis (http://gliovis.bioinfo.cnio.es/). Spatial and single-cell sequencing data were analyzed using Loupe cell browser software (v7) from 10X Genomics. Raw data were downloaded from the 10X Genomics website with consent.

### Data Availability

This study was approved by Danish law and required informed consent, but no personal data are disclosed. Gene expression data is available on NCBI GEO (GSE242838). Whole-genome and exome sequencing data are identity-sensitive and not publicly available per Danish legislation.

### Statistical Analysis and Data Visualization

Data analysis and visualization were performed using GraphPad Prism, R software, Biorender, and Adobe Illustrator. All grouped data were expressed as the mean ± SD of at least 3 independent experiments, unless otherwise specified in the figure legend, all statistical analysis tests were 2-tailed, and a *P* or FDR value of <.05 was considered statistically significant. Where applicable 2-sample *t*-tests, and linear models were used, and the resulting *P* values are reported in the Figure legend.

## Results

### Clinicopathologic Characteristics of Metabolic Stereotactic Tumor Lesions

**Figure 1.**
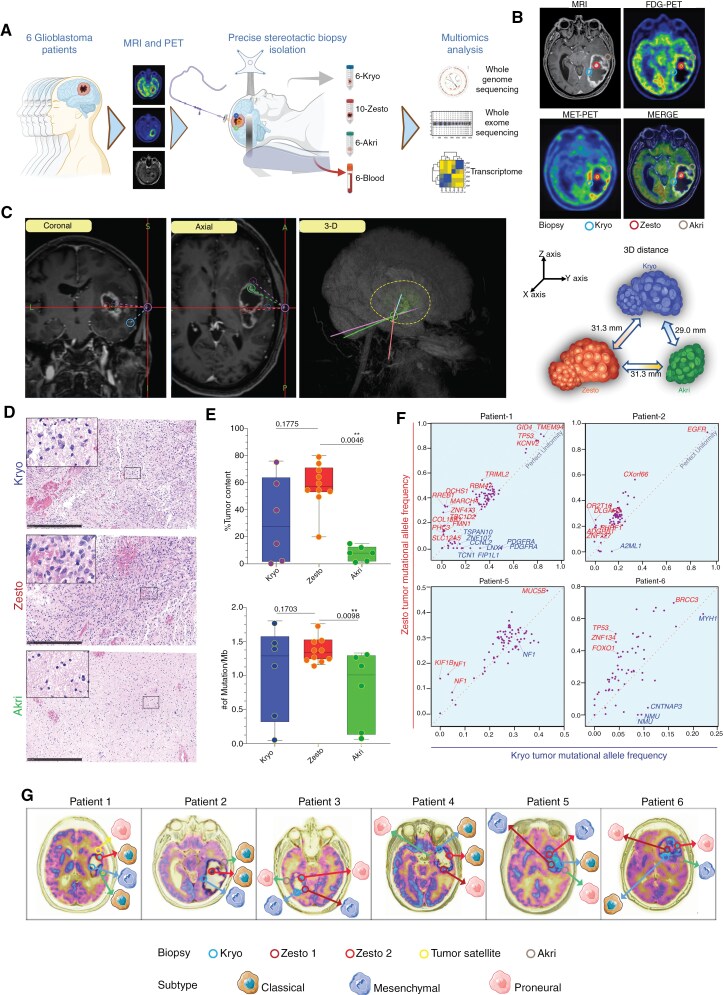
Summary of the stereotactic biopsy procedure, experimental design, and characterization of metabolic tumor lesions in glioblastoma: **(A)** The schematic representation illustrates the experimental setup designed to investigate molecular differences among hypometabolic (kryo), hypermetabolic (zesto), and non-metabolic (akri) activities in glioblastoma lesions. This configuration includes a framed stereotactic needle biopsy, a minimally invasive procedure that requires pre-MRI, MET-PET, or FDG-PET scans, guided by the predetermined location of lesions exhibiting distinct metabolic activities. **(B)** Images from MRI, [^18^F] FDG-PET, [^11^C] MET-PET, and co-registration of a representative glioblastoma patient (patient 2) reveal the locations of kryo, zesto, and akri lesions. Blue indicates the lowest, while red indicates the highest accumulation of radiotracers (additional patient data is presented in Supplementary Figure 2). The metabolic lesion regions indicated in this image are represented in 2 dimensions. However, please note that these lesions are distributed in 3-dimensional space and may not reside within the same plane. The circled areas represent only an approximate 2-dimensional projection, as depicted in Figure 1C. **(C)** A representative 3-dimensional geometric localization of lesions is depicted in the coronal, axial, and 3D views from a patient. The blue circle on the skull shows the entry point for the stereotactic needle The yellow dashed line represents the tumor area, and the green circle denotes the point of stereotactic needle insertion for lesion retrieval. The illustration demonstrates the 3-dimensional distances between kryo, zesto, and akri lesions (to the right). **(D)** Visualization of cellularity is achieved through HE-stained kryo, zesto, and akri lesions, with a scale bar of 500 µm. The inserts provide a higher magnification of a representative area. **(E)** Box plots illustrate tumor fraction estimates based on exome analysis (top) and the analysis of tumor mutational burden in kryo, zesto, and akri lesions (bottom). Error bar limits represent minimum to maximum values, with the center line indicating the median. Statistical significance was determined using an unpaired *t*-test. **(F)** Tumor mutational allele frequency analysis, conducted through whole-exome sequencing in autologous kryo (x-axis) and zesto (y-axis) lesions from 4 glioblastoma patients. Kryo and zesto-specific variants are respectively marked with blue and red (other patients are depicted in Supplementary Figure 1a). Data points represent mutations with the red dotted line indicating perfect uniformity. **(G)** Glioblastoma subtype predictions are based on transcriptome analysis of individual metabolic lesions.

We used MRI co-registered with ^11^C-MET-PET and ^18^F-FDG-PET to precisely geo-locate lesions. A computer-guided stereotactic biopsy device was employed to obtain biopsies from these lesions. We collected 23 biopsies, including 6 hypometabolic, 10 hypermetabolic, 1 tumor satellite, and 6 peripheral lesions, along with corresponding blood samples from 6 glioblastoma patients (Figure 1A, Supplementary Table S1). In our study, we used a 2 mm diameter stereotactic needle cannula inserted through a cranial burr hole, allowing us to obtain tissue with high targeting accuracy. Tumor regions with high ^18^F-FDG and ^11^C-MET uptake were classified as zesto, low uptake as kryo, and no uptake as akri (Figure 1B, Supplementary Figure 2). Biopsies from akri areas were taken at the tumor-to-normal-brain transition and from non-eloquent areas outside the tumor.

The average 3D spatial distance between autologous lesions measured with the StealthStation Surgical Navigation System ranged from 29 mm (kryo to akri) to 31.3 mm (kryo to zesto and zesto to akri; Figure 1C). Histological analysis revealed the highest cellular density in zesto lesions followed by kryo and akri lesions (Figure 1D and Supplementary Figure 1B). DNA from each lesion and blood sample was used for whole-exome sequencing (WES) and whole-genome sequencing (WGS), with RNA for transcriptome analysis. The median coverage was 100-fold for WES and 25-fold for WGS. We identified 438 single-nucleotide variants (SNVs) in WES and 700 structural variants (SVs) and 882 copy number variations (CNVs) in WGS (Supplementary Tables S2 and S3). Tumor content and mutational burden analyses showed no significant differences between kryo and zesto lesions, but akri lesions had significantly lower tumor content than zesto lesions. Despite the low tumor content in akri lesions, similar mutational calls were observed in akri and kryo lesions (Figure 1E).

We performed a tumor mutational allele frequency analysis between kryo and zesto lesions with similar tumor content from glioblastoma patients. Certain mutations had higher frequencies in zesto than kryo lesions in most patients (patients 1, 2, 5, and 6; Figure 1F and Supplementary Figure 1A). For instance, in patient 1, *RREB1*, *DCHS1*, and *MARCH4* mutations had a mean Variant Allele Frequency (VAF) of 35% in zesto lesions, while *PDGFRA*, *LNX1*, *FIP1L1*, and *CCNL2* had higher VAFs in kryo lesions. Patient 2 had zesto-specific mutations in *DLGAP3*, *OR2T10*, *PHRF1*, *ADGRB1*, and *ZNF727*, while *A2ML1* was exclusive to kryo. Patient 5 had 3 NF1 variants in zesto lesions, and patient 6 had *TP53*, *ZNF134*, and *FOXO1* mutations. SNV analysis revealed a distinct mutational pattern in zesto lesions, characterized by more specific subclonal variations than in kryo lesions. The median exome-wide mutational burden was 1.4 mutations/Mb (Figure 1E), comparable to previous reports.^16,17^ Zesto lesions stand out as the most mutated samples, with no significant difference from kryo, but significantly higher than akri lesions. Furthermore, we used predefined gene signatures to understand the transcriptional heterogeneity and subclass of these intratumoral lesions.^9^ We noticed a distribution of transcriptional subtypes with 8 proneural, 8 classical, and 7 mesenchymal subtypes across the 23 different lesions, in line with previous findings^10^ (Figure 1G). Among 10 hypermetabolic lesions, 4 were proneural, 3 mesenchymal, and 3 classical. Hypometabolic lesions were equally split between mesenchymal and classical. Akri lesions were mostly proneural. Overall, the multisectoral lesions obtained from the 6 patients display distinct subtypes at the transcriptional level that was independent of its metabolic state measured by PET.

### Glioblastoma Hypermetabolic Lesions Evolve From Hypometabolic Lesions

**Figure 2.**
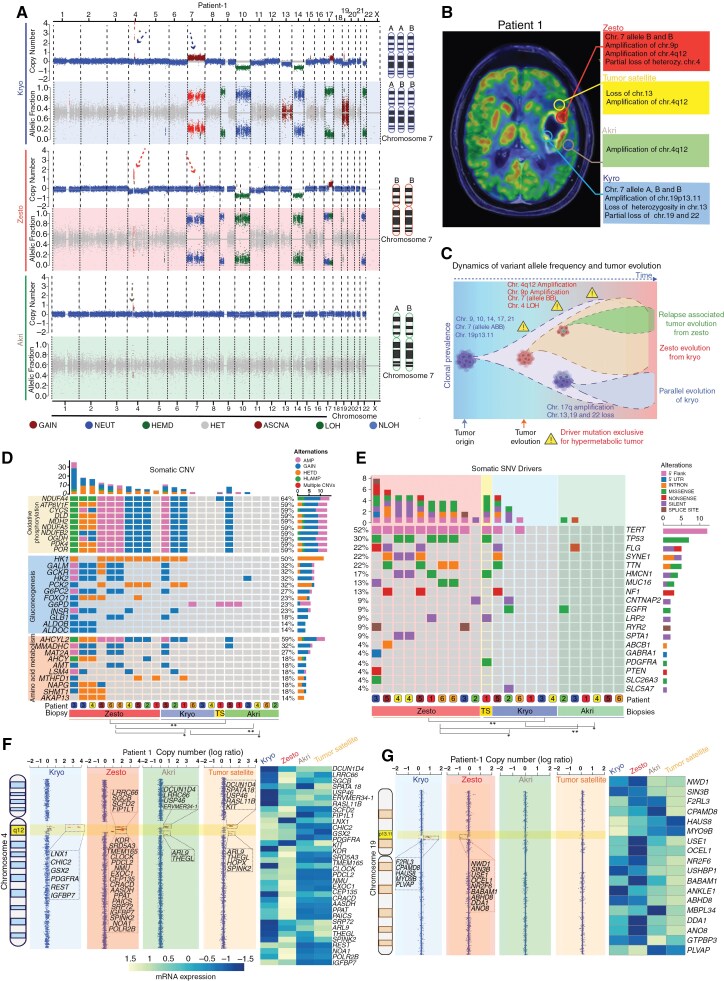
Hypermetabolic (Zesto) lesions strategically harbor pathway-specific CNVs and driver SNVs for evolving from kryo lesions. **(A)** Copy number variation plot from whole genome sequencing (top) and B allele frequency (BAF) plot from whole exome sequencing analysis (bottom) from kryo, zesto, and akri lesions. The x-axis depicts the genomic location. Arrows on chromosomes 4 and 7 show focal amplification, and chromosomal aberrations, respectively. A schematic representation of the number of alleles from chromosome 7 is indicated on the right side of the plots. **(B)** Merged transverse MRI and FDG-PET images showing different geolocations of lesions. Textboxes represent specific chromosomal alterations in the autologous lesions from patient#1, and data from other patients is represented in Supplementary Figure S3. **(C)** A schematic tumor evolution model based on variant allele frequencies and chromosomal alterations illustrates the emergence of a zesto lesion from a kryo lesion and the parallel evolution of a kryo. Zesto lesion-associated focal amplification of chromosome-4q12 was also observed in the akri and tumor satellite, and these subclones of tumor in the akri lesion may contribute to tumor recurrence. Triangles represent the driver mutations found only in the zesto biopsies. **(D)** Oncoplot showing the most prevalent putative driver mutations and CNAs in the metabolic zesto, kryo, and akri lesions of human glioblastoma grouped by metabolism pathways. (TS = tumor satellite). **(E)** OncoGrid plot from WES analysis showing genetic alterations (5′ flank, 5′ UTR, intron, missense, nonsense, silent, and splice site) of the most frequently mutated genes observed in glioblastoma. The top bar plot indicates the total no of genes altered in the corresponding patient lesion, and the right bar plot shows the SNV type and number of cases affected. The bottom color panel indicates the patient number and lesion type, TS represents the tumor satellite. **(F)** Copy number plots reveal focal amplifications on chromosome 4q12 and chromosome 19p13.11 **(G)** through whole-genome sequencing of autologous kryo, zesto, tumor satellite, and akri lesions. The dashed box highlights the amplicons and provides a list of implicated genes. mRNA expression analysis of the genes within the amplicon is conducted in zesto lesions. Genes are organized in chromosomal order to illustrate the pattern of copy number variation (CNV) and the differences in gene expression among autologous kryo, zesto, tumor satellite, and akri lesions.

To explore chromosomal abnormalities, we used somatic copy number variations and B allele fraction (BAF) analyses of autologous kryo, zesto, and akri lesions. Analysis of patient-1 showed 3 copies of chromosome 7 (allele ABB) in kryo, but only 2 copies of chromosome 7 (allele BB) were present in the zesto lesion (Figure 2A). This indicates that the flow of evolution occurred from the hypometabolic to the hypermetabolic tumor, as the A allele was lost in the hypermetabolic state. Additionally, in the zesto biopsy, we observed a specific loss of chromosome 4 and micro amplification of the chromosomal region 4q12. Remarkably, we observed deletion of chromosomes 4, 13, 19, and 22 only in kryo biopsy, which suggests parallel and spatially localized evolution of the hypometabolic area of the tumor. Indeed, we observed a marked increase in BAF for chromosomes 7 and 10 in all hypermetabolic lesions, as well as elevated BAF for multiple chromosomes, which varied among patients (Supplementary Figure 8). To visualize these key spatial differences, we mapped the chromosomal abnormalities on the merged transverse MRI and concordant FDG-PET images, which showed intratumoral heterogeneity between different autologous lesions (Figure 2B and Supplementary Figure 3). Overall, these findings indicated that hypermetabolic tumors evolved from hypometabolic regions, as schematically illustrated in the tumor evolution model (Figure 2C).

Next, we sought to outline specialized mutational developments through copy number alteration (CNA) analysis that may influence metabolic transformation to hypermetabolic tumors. Analysis of signature genes from oxidative phosphorylation, gluconeogenesis, and amino acid metabolism pathways revealed that 90% of zesto lesions had amplification, gain, and/or high-level amplifications of *NDUFA4, ATP6V1F, CYCS, DLD, MDH2* (oxidative phosphorylation), 40%–60% of lesions had alterations of *GALM, GCKR, HK2, G6PC2, INSR* (gluconeogenesis) and 30%–70% of lesions had alterations in *AHCYL2, MMADHC, MAT2A, AMT, LSM4* (amino acid metabolism; Figure 2D). The statistically significant presence of the most common mutations and CNAs in metabolic pathways in hypermetabolic lesions suggests that these mutations may have been acquired later in the tumor’s evolution to facilitate its higher metabolic demands, whereas a low frequency of these CNAs in some hypometabolic lesions may indicate their future transition to a hypermetabolic tumor.

### Known Driver Mutations are More Frequent in Hypermetabolic Lesions

Driver genes play a critical role in glioblastoma evolution, and frequent mutations in *TP53, EGFR, PTEN,* and *TERT* genes have been well-defined.^9,18,19^ We analyzed SNVs to observe the frequency and alterations of recognized glioblastoma drivers in hypermetabolic lesions. Intriguingly, *TERT*, *TP53*, *FLG*, *SYNE1*, and *TTN* were the most frequently mutated genes in zesto lesions at 90%, 60%, 30%, 40%, and 40%, respectively (Figure 2E). In contrast, only 1 to 2 out of 12 lesions were found to have alterations in kryo and akri lesions for the majority of driver genes. Additionally, the frequency of *TERT* and *TP53* alterations in our cohort was per earlier studies that observed mutations in approximately 30%–50% of GBM samples. However, the frequency of *EGFR* alterations was lower,^20,21^ whereas *FLG* and *SYNE1* frequencies were higher than those in previous reports.^21^ Surprisingly, we observed only *EGFR* mutations in one kryo and akri lesion. These analyses suggest that hypermetabolic lesions have a significantly greater occurrence of recognized driver mutations than hypometabolic lesions. Such mutations may significantly influence the transformation of hypometabolic lesions into hypermetabolic ones.

### Hypermetabolic Tumor Lesions Harbor Shrewd Focal Amplifications and Deletions

Next, we examined the heterogeneity of autologous lesions in patients with glioblastoma at the sub-chromosomal level. To achieve this, we analyzed chromosomal gains and losses in minimal common regions (MCRs) of chromosomes 4q12 and 19p13.11 using the Titan-CNA tool^22^ (Figure 2, F–G). Alterations identified by Titan-CNA in the 4q12 MCR covered recognized oncogenes and genes with known biological significance in glioma and glioblastoma, including *PDGFRA, REST,* and *CLOCK*^9,23,24^ (Figure 2F box). Interestingly, in a previous study, amplification of 4q12 was detected in 15% of GBMs cases.^25^ In addition, in the zesto biopsy, we detected amplified genes (*NMU*, *CHIC2*, *IGFBP7*, *TMEM165*, *PPAT*, *CEP135*, and *POLR2B*; Figure 2F). Moreover, 19p13.11 MCR contained a region with a low copy number of *BABAM1, USE1, NWD1, DDA1, ANO8, OCEL1*, and *ABHD8*. To analyze the impact of these CNVs, we performed gene expression analysis of the 4q12 and 19p13.11 MCRs, which confirmed that these focal genomic alterations led to an altered mRNA expression for most of these genes throughout all the biopsies (Figure 2, F and G heatmaps). These findings suggest that zesto tumors contain strategic, localized amplifications and deletions of genes that cause alterations in transcription, potentially affecting the metabolism and facilitating tumor evolution.

### The Genomic Landscape of Hypermetabolic Lesions Predict Potential Pathways for Metabolism

We identified 1120 genes in metabolic critical pathways of hypermetabolic lesions through global CNV analysis, revealing shared and unique features across patients (Supplementary Figure 5A–D). Protein-protein interaction (PPi) analysis highlighted pathways, including transcription initiation, proto-oncogenes, oxidative phosphorylation, and circadian rhythm (Supplementary Figure 3B). Moreover, hypermetabolic biopsy-specific CNVs exhibited amplified mRNA expression in tumor cells, correlating with poor survival in LGG and GBM patients (Supplementary Figure 4, A–B, C–D). Our analysis revealed critical pathways and gene sets essential for hypermetabolism and increased tumor aggressiveness.

### Hypermetabolic Lesions Exhibit a High Frequency of Fusion, Omikli, Chromothripsis, and Chromoplexy Events

**Figure 3.**
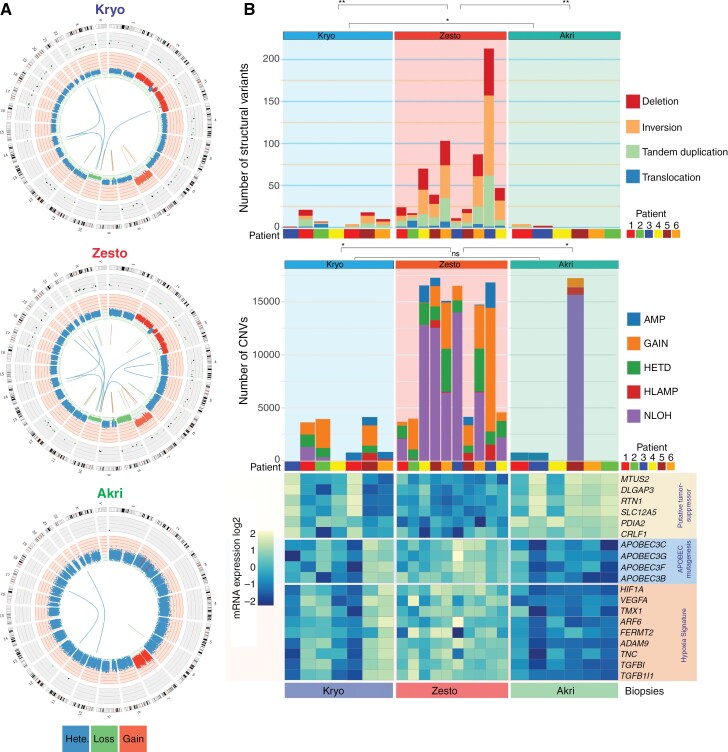
Occurrence of genomic rearrangements and chromothripsis events in the hypermetabolic lesions. **(A)** Representative scaled circos plots of the whole genome of autologous lesions from a glioblastoma patient#5 showing genomic rearrangements. The outer ring shows the SNVs from the whole exome sequencing analysis, and the middle ring indicates the copy number variation from the whole exome sequencing, blue color represents heterozygosity, the red color indicates the gain, and the green indicates the loss of chromosomes. The innermost circle shows the structural variant analyzed by whole genome sequencing. **(B)** multi-omics global overview of structural variants in glioblastoma kryo (light blue background), zesto (light red background), and akri (light green background) lesions. The top bar plot shows the deletion, inversion, tandem duplication, and translocation in individual lesions. The second bar plot shows the tumor content in respective lesions, numbers represent the percentage of tumor content based on the whole exome sequencing estimation. The third bar plot indicates copy number variation such as (AMP) amplification, gain, (HETD) heterozygous deletion, (HLAMP) high-level amplification, (NLOH) neutral loss of heterozygosity, and (LOH) Loss of heterozygosity. The bottom heatmap shows the mRNA expression of putative tumor suppressor genes (*MTUS2, DLGAP3, RTN1, SLC12A5, PDIA2,* and *CRLF1*), APOBEC mutagenesis-associated genes, and hypoxia gene signature in the representative kryo, zesto, and akri lesions.

Gene fusions are known to favor tumorigenesis, play an oncogenic role, and provide an opportunity for therapeutic targeting of glioblastoma and other cancer.^26,27^ We detected 182 fusion transcripts, including 26 deletions, 45 duplications, 31 inversions, and 5 translocations. Zesto lesions had significantly more fusion events than kryo and akri lesions (Figure 3A, Supplementary Figure 6–7 and Table S4). Notable alterations in zesto lesions included *PDGFRA-FIP1L1*, *PTPRZ1-MET*, *MTMR2-CEP57*, and *PDGFRA-TMEM165*.

To assess genomic phenomena like omikli (Diffuse hypermutations), kataegis (localized hypermutations), chromothripsis (clustered chromosomal rearrangements), and chromoplexy(complex DNA rearrangements), we analyzed WES and WGS data. Zesto lesions had significantly more translocations, duplications, tandem inversions, and deletions than kryo and akri lesions (Figure 3B, top panel). Zesto lesions also had more frequent genomic events like amplification, gain, heterozygous deletion, high-level amplification (HLAMP), and copy-neutral loss of heterozygosity (NLOH). High genomic alterations in zesto lesions were inversely correlated with putative tumor suppressor genes (*MTUS2, DLGAP3, RTN1, CRLF1, SLC12A5*, and *PDIA2*) and positively correlated with APOBEC mutagenesis (*APOBEC3C, APOBEC3G, APOBEC3B*, and *APOBEC3F*) and hypoxia-related gene signatures (Figure 3B, bottom panel). Chromothripsis (40%) and chromoplexy (20%) were detected in zesto lesions, with dominant C→T and T→C mutations in hypermetabolic lesions, contrasting frequent omikli in hypometabolic lesions. Despite absent kataegis, elevated gene fusions, chromothripsis, and chromoplexy likely drive glioblastoma hypermetabolism, mediating transitions from hypometabolic to aggressive hypermetabolic states (Supplementary Figure S6, S7B).

### Regional Transcriptomic Landscape of the Developing Metabolic Lesion in Glioblastoma

**Figure 4.**
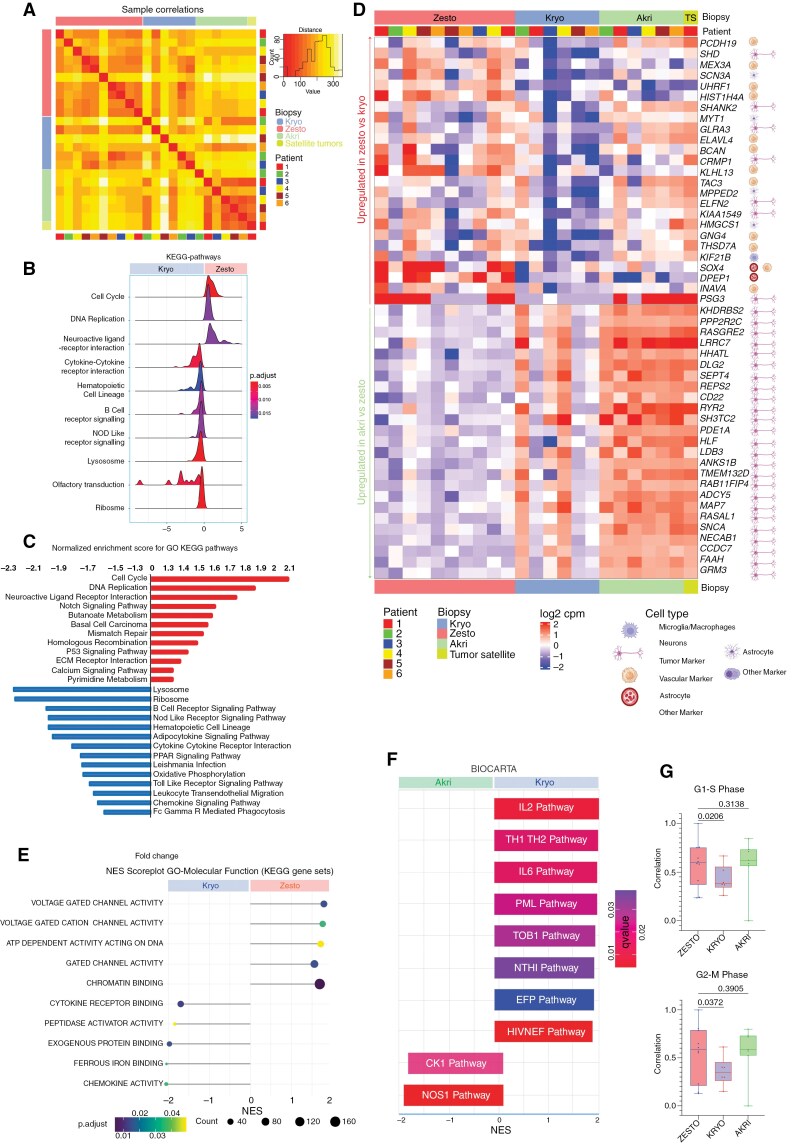
Spatial bulk transcriptomic analysis reveals metabolic and immunological relevance in hypermetabolic lesions.Bottom of Form. **(A)** Heat map of the transcriptome correlation matrix displaying transcript expression across lesions with varying metabolic activity (23 samples). The cluster dendrogram and Spearman correlation coefficient heatmap are generated using normalized TPM (transcripts per million mapped reads) values of expressed transcripts. A higher correlation is represented in dark, while a lower correlation is depicted in light. The legend for transcriptomic distance, lesion type, and patient is included on the right side. **(B)** The ridge plot illustrates the fold change in expression difference of core-enriched genes within major Kyoto Encyclopedia of Genes and Genomes (KEGG) pathways between Zesto and kryo lesions. The color gradient represents the adjusted *P*-value. **(C)** The bar plot illustrates the normalized enrichment scores for KEGG pathways in Zesto lesions (depicted in red) and Kryo lesions (depicted in blue). Apart from the observed upregulation in the cell cycle and DNA replication pathways, Zesto lesions also exhibit increased activity in the butanoate and pyrimidine metabolism pathways. **(D)** Heatmap depicts the expression of top-upregulated and downregulated genes in each metabolic lesion. The scale bar represents average gene expression (log2 cpm). Cellular icons denote the highest expression observed in respective cell types based on single-cell mRNA analysis. **(E)** Score plot illustrates normalized enrichment scores (NES) of KEGG gene sets (molecular functions), indicating significantly altered pathways between Zesto and akri lesions. The circle color corresponds to the p-value of NES, while circle size represents the number of genes. **(F)** NES score plot illustrates top-ranked BIOCARTA pathways, revealing significant pathway differences between kryo and akri lesions. Bar color corresponds to the *q*-value. **(G)** Bar plot depicts a higher correlation (Pearson correlation r) of cell cycle pathways (G1-S and G2-M phase) in zesto lesions compared to kryo lesions. Gene set sourced from Neftel et al.

To understand the functional complexity of lesions, we conducted bulk transcriptomic analysis on 23 glioblastoma lesions. Our analysis revealed distinct clustering among zesto, kryo, and akri lesions (Figure 4A). Zesto lesions showed significant upregulation of pathways related to the cell cycle, DNA replication, and neuroactive ligand-receptor interaction, indicating heightened cellular proliferation and metabolic activity (Figure 4B). In contrast, kryo lesions had increased enrichment scores for lysosome, ribosome, and immune cell-related pathways, suggesting involvement in immune response, lysosomal function, and protein synthesis (Figure 4C).

Single-cell expression analysis revealed that most upregulated genes in zesto lesions were expressed within the tumor, with few in astrocyte and vascular cell clusters (Figure 4D). dipeptidase 1 (*DPEP1)* and *SOX4* were primarily expressed in vascular cells and were notably upregulated in hypermetabolic lesions. Conversely, genes overexpressed in akri regions were mainly in neuronal, OPC, and astrocyte cell types (Figure 4D). Further molecular function analysis showed higher enrichment scores for various voltage-gated channels and DNA-binding activities in zesto lesions compared to kryo lesions (Figure 4, E–G). Kryo lesions also exhibited increased immunological activity, including pathways involving IL-2, TH1-TH2 cytokines, IL-6, PML, and HIVNEF, compared to akri lesions (Figure 4F). Bulk analysis (CGGA cohort) showed that *DPEP1* was significantly upregulated in chemotherapy-treated patients, while *BCAN* and *SHD* were downregulated. No significant differences in gene expression for any of these genes were found for patients who were treated with radiotherapy (Supplementary Figure S12).

### *DPEP1* as a Novel Marker for Hypermetabolic Lesion of Glioblastoma

**Figure 5.**
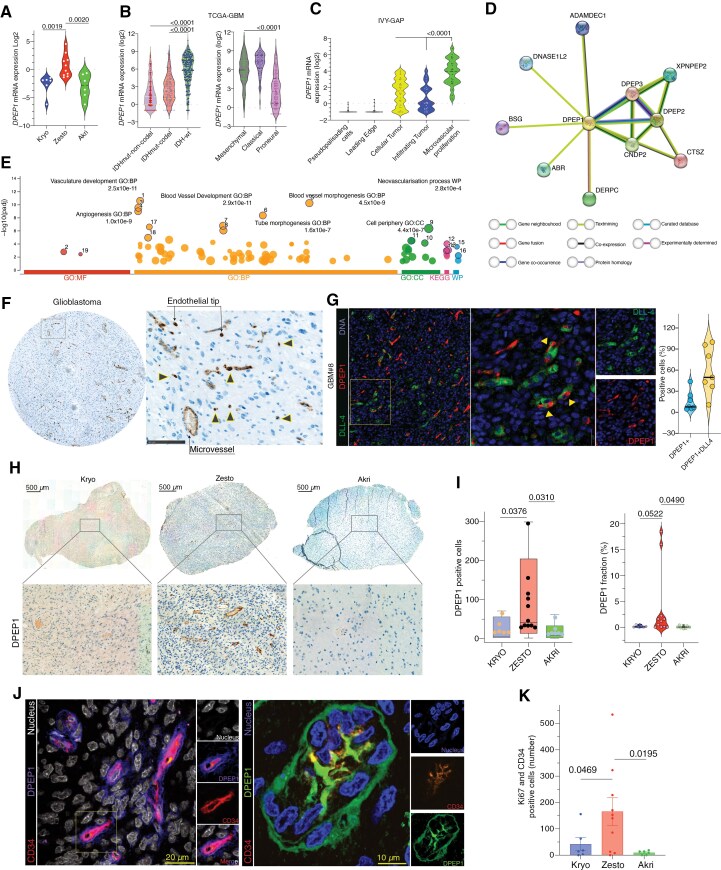
Dipeptidase 1 is a novel marker for vascular tip endothelial cells that is upregulated in hypermetabolic lesions. **(A)** mRNA expression (log2) of the dipeptidase 1 gene (*DPEP1*) was examined in the kryo, zesto, and akri lesions. Statistical differences in mRNA expression were evaluated using a 2-tailed *t*-test. **(B)** mRNA expression (log2) of the *DPEP1* in the TCGA-GBM-LGG database is depicted, contrasting IDH-wt (glioblastoma) with IDH-mt (low-grade glioma; shown in the left violin plot) and in various glioblastoma subtypes is illustrated in the right violin plot. Statistical significance (*P* < .01) was analyzed by one-way analysis of variance (ANOVA) and is indicated with an asterisk. **(C)** mRNA expression of *DPEP1* in the IVY-GAP dataset shows higher expression in microvascular proliferation and cellular tumor region of glioblastoma. **(D)** A connectivity map illustrating the significant proteins that interact with the dipeptidase 1 enzyme. The colors of the nodes are depicted in the key provided below. **(E)** Gene ontology analysis of the top 50 positively correlated genes with DPEP1 reveals pathways related to tube morphogenesis across molecular function, biological process, cellular components, and KEGG pathways. The analysis was conducted using g:Profiler. **(F)** Representative immunohistochemistry images of dipeptidase 1 in human glioblastoma reveal its localization around vascular structures. Scale bar of 100 µm and 250 µm. Additional images from the positive control and glioblastoma patients are provided in Supplementary Figure S10 A–B. Black triangles represents the endothelial sprouts structures. **(G)** Immunofluorescence staining of DLL-4 and DPEP1 in a glioblastoma section. Triangles indicate the colocalization of the endothelial tip cell marker DLL-4 with DPEP1. The quantification of DPEP1 colocalization with DLL-4 is shown to the right of the figure (full GBM section, *n* = 7) **(H)** Representative IHC images and quantification of dipeptidase 1 in zesto, kryo, and akri micro lesions. **(I)** Bar and violone plots indicate the DPEP1 positive cell/fraction per lesion. The indicated *P*-value is derived from a paired *t*-test. **(J)** Representative confocal microscopy images depict the colocalization of dipeptidase 1-positive cells and CD34-positive cells (in red) in the glioblastoma tissue section. Additional images are available in Supplementary Figure 9 G. **(K)** Plot showing quantification of CD34 and Ki67 positive cells from immunofluorescence image analysis in zesto, kryo and akri lesion of glioblastoma, exact *P* values are indicated from *t*-test.

Our analysis revealed *DPEP1* as one of the most significantly upregulated genes in hypermetabolic glioblastoma lesions compared to both kryo and akri lesions (Figure 5A). To further validate the clinical relevance of *DPEP1*, we analyzed the The Cancer Genome Atlas-Glioblastoma-Low Grade Glioma (TCGA-GBM-LGG) dataset, revealing significantly higher expression of *DPEP1* in isocitrate dehydrogenase (IDH)-wild type tumors compared to lower-grade gliomas with *IDH* mutation. Subsequent analysis unveiled that the classical and mesenchymal glioblastoma subtype exhibited markedly elevated expression of *DPEP1* relative to the proneural subtype (Figure 5B, Supplementary Figure 9A–B). Further histological transcriptomics analysis from the IVY-GAP dataset revealed the highest expression of *DPEP1* within the microvascular proliferation region of the glioblastoma tumor (Figure 5C).

### Dipeptidase 1 Expression is Localized to Endothelial Tips of Hypermetabolic Lesions

To gain deeper biological insights and understand the direct interactions of *DPEP1* with other key proteins, we conducted a STRING analysis. This analysis revealed a network involving *DPEP1, DPEP2, DPEP3, ADAMDEC1, DNASE1L2, AGR, DERPC, CTSZ,* and *CNDP2* genes, linked to various cellular processes including peptide metabolism, immune response, cell adhesion, and protein/DNA degradation (Figure 5D). We further dissected the functional pathways associated with *DPEP1* using gene ontology analysis on the top of 50 significantly positively correlated genes from the glioblastoma dataset (TCGA), revealing enrichment in pathways related to vascular development, neovascularization, and tube morphogenesis (Figure 5E).

IHC was employed to validate the spatial distribution of DPEP1 within the glioblastoma microenvironment, revealing localization predominantly at vascular tips and sprouting endothelial structure (Figure 5F, Supplementary Figure S10A-B). Subsequent analysis of stereotactic biopsies demonstrated significantly higher cell numbers of DPEP1-positive cells in zesto lesions compared to akri and kryo lesions (Figure 5H-I). Markedly, DPEP1 expression was absent in normal brain regions at the IHC level (Supplementary Figure S9G, S10B). Notably, we observed strong DPEP1 staining on microvascular tips, marked by DLL-4 co-staining and spatial mRNA expression (Figure 5G, and Supplementary Figure S11A-Top panel, S11B) on vascular sprouts, in contrast to the weaker staining observed in mature vascular structures in glioblastoma (Supplementary Figure 10D). This suggests the crucial role of dipeptidase 1 in the endothelial sprouting. Additionally, confocal imaging of human glioblastoma sections confirmed the vascular localization of dipeptidase 1, exhibiting colocalization with the endothelial cell marker CD34 (Figure 5J, Supplementary Figure 9G, 10C, Figure 9 E–F^28^). To assess the total microvascular proliferative cells, we quantified the CD34 and Ki67-positive cells in various metabolic lesions. Our analysis revealed a significantly higher number of Ki67 and CD34 positive cells in the zesto lesions compared to the akri and kryo lesions (Figure 5K).

### Mechanistic Insights of DPEP1 and its Role in Hypermetabolic Lesions

**Figure 6.**
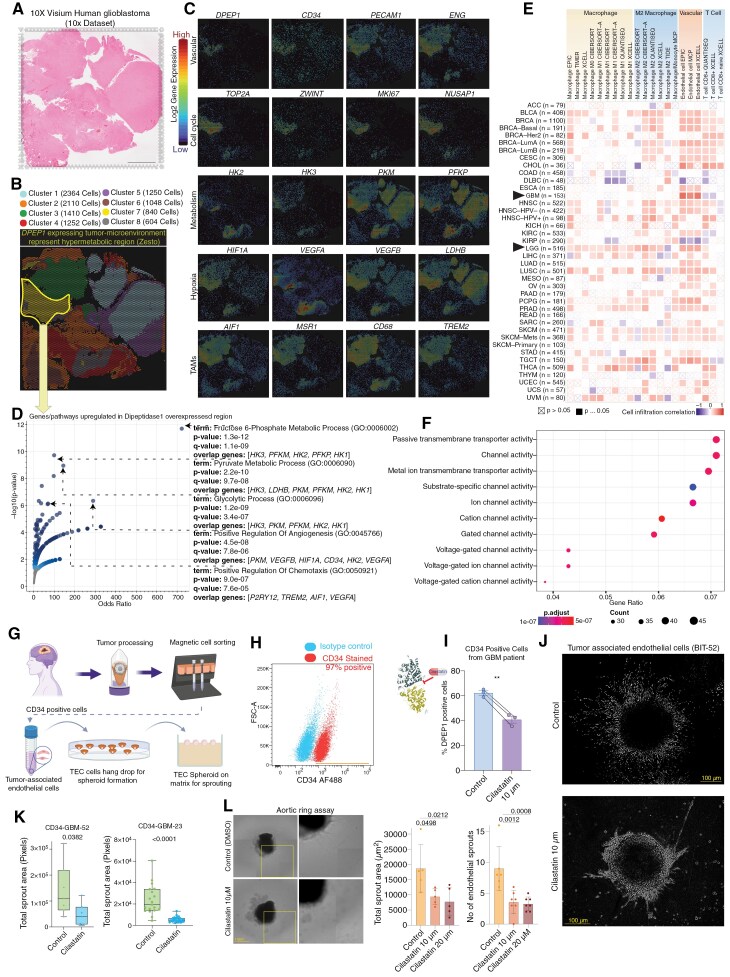
Mechanistic insights of dipeptidase 1 and its role in hypermetabolic lesion. **(A)** Morphological regions were annotated via H&E staining of a human glioblastoma tumor fixed onto the Visium Spatial Transcriptomic slide (10X Genomics Dataset), with a scale bar of 2.5 mm. **(B)** The figure indicates histological clusters based on k-means clustering of the transcriptome. Cluster 7, comprising 840 cells, exhibits the highest expression of dipeptidase 1 (DPEP1), indicating a hypermetabolic lesion-like cluster in the human glioblastoma tissue section. The total barcode count is 10,878. **(C)** The mRNA expression (log2) of genes, displaying the spatial distribution from various pathways (metabolism, cell cycle, and angiogenesis), illustrates the colocalization and enrichment with DPEP1-positive lesions. **(D)** Differential Gene ontology analysis of DPEP1 expressing regions with surrounding DPEP1 negative regions from 3 glioblastoma 10X dataset, the volcano plot illustrates terms from the GO Biological Process 2023 gene set. Each point represents a single term, plotted by the corresponding odds ratio (x-position) and log10(*P*-value) (y-position) from the enrichment results of the input query gene set. The larger and darker-colored the point, the more significantly enriched the input gene set is for the term. The odds ratio and the enrichment analysis *P*-value are mentioned next to the top 5 pathways. **(E)** The plots indicate a systematic analysis of immune infiltrates across diverse cancer types in DPEP1 overexpressing samples, with data analysis performed by Timer 2.0. A positive correlation of endothelial cells and a negative correlation of T cells were significantly enriched in GBM patients. **(F)** Gene ontology enrichment analysis of dipeptidase 1 for molecular function, conducted using the TCGA glioblastoma dataset, reveals its involvement in the regulation of transporters and ion channel activity. The color of the circle represents a level of significance, while the size indicates the number of genes in the pathways. **(G)** Schematic representation of the setup for purifying CD34-positive endothelial cells from human glioblastoma tumors and establishing the endothelial sprouting assay. Created with Biorender. **(H)** Representative FACS dot plot showing CD34 expression in purified endothelial cells isolated from human glioblastoma. **(I)** Bar plot showing expression of DPEP1 expression after cilastatin treatment for 72 hours, analyzed by flow cytometry. Actual *P* value from *t*-test. The structure of dipeptidase 1 was analyzed using the Litemol tool from the EBI Enzyme Portal. Cilastatin is identified as an inhibitor for dipeptidase 1. **(J)** Representative image showing sprout of glioblastoma derived endothelial spheroids with and without cilastatin, stained with Hoechst 33342. The images were captured at 20× magnification, with a scale bar of 100 µm. **(K)** Bar plot illustrating the effect of a dipeptidase 1 inhibitor on angiogenic sprout formation, measured by total sprout area in 2 different primary glioblastoma-derived endothelial cell lines. The displayed *P*-value was obtained from a paired *t*-test. **(L)** Phase-contrast images of aortic rings embedded in type I collagen and Geltrex™, respectively, showing micro vessel outgrowth and inhibition after cilastatin treatment. Scale bar 1mm. Right panel, bar plot illustrating the effect of a dipeptidase 1 inhibitor on angiogenic sprout formation, measured by total sprout area in mouse-derived aortic ring assay. Statistical analysis was performed by one-way analysis of variance (ANOVA) followed by Tukey’s post hoc tests. Adjusted *P*-values are indicated.

To delve into the downstream pathways and gain mechanistic insights into DPEP1, we employed spatial transcriptomics datasets (Visium, Glioblastoma, FFPE fixed, 10X Genomics; Figure 6A). Within this dataset, comprising 10,878 spots detected under tissue, the median UMI counts per spot were 8339, with a median of 4600 genes per spot. Clustering analysis identified 8 distinct clusters, with cluster 7 demonstrating the highest expression levels of *DPEP1* within the glioblastoma tissue section (Figure 6B). *DPEP1* overexpression was localized to specific regions of glioblastoma lesions (Figure 6C). Within cluster 7, *DPEP1* expression coexisted with *CD34*, *PECAM1*, and *ENG* genes, indicating a microvessel-rich region. Interestingly, not all *CD34* and *PECAM1*-positive domains exhibited *DPEP1* gene expression, confirming selective expression within vascular structures. Co-expression analysis revealed correlations between *DPEP1*-expressing regions and cell cycle (*MKI67*, *TOP2A*, *ZWINT*, and *NUSAP1*) and metabolism-related (*HK2*, *HK3*, *PKM*, and *PFKP*) genes in cluster 7 of the GBM section (Figure 6C). Gene signatures associated with GBM stem cells and pericytes were highly expressed in hypermetabolic lesions, which are characterized by elevated *DPEP1* mRNA expression, as compared to hypometabolic lesions. However, the immune cells gene signature displayed heterogeneity, with variations observed from region to region, consistent with our immunohistochemistry and bulk mRNA analysis (Supplementary Figure S11A).

To evaluate the abundance of total myeloid cells (*IBA1* and *AIF1* genes), endothelial cells (*CD34*), and neutrophils across various metabolic lesions, we analyzed mRNA sequencing data and conducted immunohistochemistry for key markers. Our analysis showed no significant differences in the expression of *AIF1*, *CD34*, or neutrophil gene signatures among these lesions (Supplementary Figure 10 E–H). However, we observed an increased expression of the *ELANE* (Elastase, Neutrophil Expressed) in hypermetabolic lesions (Supplementary Figure S10H).

We conducted a differential gene expression analysis across 3 spatial transcriptomics datasets (Visium, Glioblastoma, FFPE fixed, 10X Genomics), analyzing various spatial clusters and performing gene ontology. Significant upregulation was observed in terms including fructose 6-phosphate metabolic process, pyruvate metabolic process, glycolytic process, positive regulation of angiogenesis, and positive regulation of chemotaxis in DPEP1-expressing lesions (Figure 6D), confirming *DPEP1*-rich microenvironments as a hypermetabolic region in the glioblastoma microenvironment. Deconvolution analysis on bulk mRNA sequencing data from TCGA revealed positive endothelial cell infiltration in *DPEP1*-overexpressing samples and predominantly negative T cell infiltration across cancers (Figure 6E). Downstream mechanistic analysis on the TCGA-GBM dataset revealed upregulation of voltage-gated ion channels and transporter activities in *DPEP1*-overexpressing tumors (Figure 6F), possibly enhancing glucose influx and amino acid metabolism in zesto lesions.

To elucidate the clinical significance of DPEP1, we assessed the impact of cilastatin, a renal dipeptidase inhibitor, on vascular structure formation in vitro and ex vivo. Using CD34-positive cells from human glioblastoma, we conducted an endothelial sprouting assay (Figure 6G–H) alongside a mouse aortic ring sprouting assay to evaluate the angiogenic capacity of DPEP1 in the presence of cilastatin. Targeting DPEP1 with cilastatin significantly suppressed DPEP1 expression (Figure 6I) and reduced the angiogenic sprout area in both glioblastoma-derived vascular sprouts (Figure 6J-K) and mouse aortic ring assays without inducing toxicity (Figure 6L, Supplementary Figure S10I), highlighting its potential as a therapeutic target. Notably, high DPEP1 expression correlates with poor survival in glioblastoma patients across multiple cohorts (Supplementary Figure 9D), while CD34 expression showed no impact on patient survival (Supplementary Figure 9H). These findings suggest that DPEP1 may play an independent and critical role in angiogenesis within hypermetabolic lesions, establishing it as a promising diagnostic and prognostic marker.

## Discussion

Although many studies have reported complex tumor heterogeneity in glioblastoma, profound intratumoral, spatial, and genomic heterogeneity and its link to tumor metabolism used for glioma imaging in the clinical setting have been loosely elaborated.^10,20,29–32^ We demonstrated that hypermetabolic lesions identified by MRI/PET imaging have a higher frequency of genomic alterations than hypometabolic lesions, and that these alterations are associated with increased aggressiveness. These findings are supported by the following novel discoveries: (1) hypermetabolic lesions genetically evolve from hypometabolic lesions; (2) hypermetabolic lesions are spatially and genomically more diverse than hypometabolic lesions and associated with a higher frequency of fusion, omikli, chromothripsis, and chromoplexy events; (3) DPEP1 is a novel diagnostic and prognostic vascular tip marker for hypermetabolic lesion (Supplementary Figure S13).

Tumor evolution involves an intricate interplay between genetic and epigenetic factors as well as spatially diverse physiological microenvironments. Pre-mutational factors, specifically hypoxia and altered intracellular pH, are crucial for creating a conducive microenvironment for transformational changes, leading to the accumulation of genetic alterations. Importantly, a correlation between higher levels of hypoxia, pH, and a higher number of driver mutations has been observed in various types of cancer.^33–35^ We showed that the high expression of hypoxia genes in hypermetabolic lesions positively correlates with the *APOBEC* gene family. APOBEC proteins, which cause the deamination of cytosine residues in both DNA and RNA, resulting in somatic mutations, RNA modifications, DNA breaks, or DNA demethylation^36–38^ show a positive correlation with hypoxia genes and tumor mutational burden in hypermetabolic lesions.

Previous studies have identified *TERT* promoter and *TP53* mutations as early events in glioblastomas and other cancers.^39–41^ Our analysis reveals that high metabolic activity in glioblastoma lesions often correlates with key driver mutations in genes like *TERT*, *NF1*, *TP53*, *FLG*, *SYNE1*, and *HMCN1*. *TERT* promoter mutations may arise during later tumorigenesis stages.^19,42^ Our findings indicate that there is a strong likelihood that driver mutations play a role in initiating transformation into hypermetabolic lesions. Clinically, detecting a *TERT* promoter mutation in an IDH-wild type astrocytic glioma is crucial for diagnosing glioblastoma.^2,43^ For diagnostic biopsies, sampling hypermetabolic lesions using PET imaging guidance is advisable, emphasizing the significance of our findings in clinical practice.

Glioblastomas are characterized by significant alterations in cellular metabolism, which serves as a prominent hallmark of these tumors.^44,45^ In glioblastomas, the Warburg effect plays a significant role in tumor metabolism. Glioblastomas exhibit a high rate of glucose uptake and preferentially utilize glycolysis as their primary energy source despite the availability of oxygen. However, in the absence of oxygen, the HIF-dependent hypoxic response regulates various cellular processes including metabolism, angiogenesis, and differentiation.^46^ Our findings demonstrated that hypermetabolic glioblastoma lesions display significantly high number of CNVs in genes involved in oxidative phosphorylation, gluconeogenesis, and amino acid metabolism pathways to meet the energy requirements of tumor cells. The markedly low CNAs observed in kryo and akri lesions may result from the presence of less infiltrative, lower-metabolic tumor clones and reduced expression of the APOBEC3 and hypoxia gene signature, which likely plays a crucial role in these genomic alterations. In addition, our results affirm the findings of a study by Wu et al.,^47^ which suggested that hypoxia is involved in inducing the natural evolutionary signature of the tumor by HIF1A.

Glioblastoma exhibits significant transcriptional heterogeneity, with distinct characteristics across lesion types. Here, we identify that hypermetabolic glioblastoma lesions are marked by high tumor mutational burden, DPEP1-positive endothelial tip cells, and abundant proliferating tumor and endothelial cells. Conversely, kryo lesions show reduced proliferative activity in tumor and endothelial cells but maintain a comparable proportion of endothelial and macrophage populations relative to zesto lesions. This suggests that zesto lesions represent mutationally and metabolically advanced tumor regions, integrating both proliferative tumor cells and vascular components. In contrast, akri lesions are enriched with neuronal cells and exhibit a sparse presence of a migrating tumor and myeloid cells, which is in line with their unique transcriptomic profiles.

Specialized endothelial cells and their complex functions in forming microvascular structures are known to promote the growth of glioblastoma.^48,49^ Our functional and mechanistic analysis revealed *DPEP1* to be a novel hypermetabolic lesion-specific vascular tip cell marker. Notably, *DPEP1* demonstrated significant upregulation in hypermetabolic lesions compared to kryo and akri lesion types at mRNA and protein levels. Spatial localization within GBM microvascular proliferation underscores DPEP1’s role in angiogenesis. Importantly, we show in vitro DPEP1 inhibition impaired angiogenesis in glioblastoma-derived endothelial sprouts, revealing the therapeutic potential of dipeptidase 1 inhibition. Thus, DPEP1 offers a promising target for drugs like cilastatin, which inhibit vascular sprouts without crossing the blood-brain barrier, potentially reducing tumor metabolic support. *DPEP1* expression correlates with pathways related to vascular development, neovascularization, and metabolic adaptation in glioblastomas. Other studies focused on dipeptidase 1, demonstrated that DPEP1 mediates neutrophil and monocyte influx in the lungs and liver by serving as a physical adhesion receptor.^50,51^ We did not observe any significant differences in the infiltration of neutrophiles and macrophages in zesto and kryo lesions, this may be due to immature endothelial sprouts in Zesto lesions, which retain extravasation barriers, hindering myeloid cell infiltration into the tumor microenvironment.

Our multi-omics analysis revealed glioblastoma’s metabolic lesion heterogeneity and evolution, with hypermetabolic lesions exhibiting advanced molecular alterations complicating therapy. DPEP1’s role in angiogenesis, metabolism, and tumor progression highlights its potential as a biomarker and therapeutic target. Stereotactic biopsies from hypermetabolic regions identified via PET/MRI and multi-omics profiling are essential for uncovering mutational traits, advancing personalized glioblastoma treatment strategies.

## Limitations of the Study

Human glioblastoma exhibit high diversity at microvasculature levels and we show that high expression of DPEP1 in endothelial sprouts of hypermetabolic lesions of glioblastoma present diversely at endothelial sprouts. However, rodent xenograft glioblastoma models do not accurately replicate the complex endothelial structures and microvascular proliferation observed in human glioblastoma. Due to these limitations, more complex in vivo models must be developed to perform in vivo assays for this specific analysis. Moreover, in our study, we used a 2 mm stereotactic needle, which allowed for precise targeting of tissue with a limited sample size. While this small sample was sufficient for transcriptomics, whole-genome, whole-exome sequencing as well as histological investigations, it restricted further omics analyses.

## Supplementary material

Supplementary material is available online at *Neuro-Oncology* (https://academic.oup.com/neuro-oncology).

noaf071_Supplementary_Tables_S1-S4_Figures_1-S13
