# Corrigendum to: Multiomic profiling of glioblastoma metabolic lesions reveals complex intratumoral genomic evolution and dipeptidase-1-driven vascular proliferation

**DOI:** 10.1093/neuonc/noag091

**Published:** 2026-05-11

This is a corrigendum to: Atul Anand, Jeanette Krogh Petersen, Lars van Brakel Andersen, Mark Burton, Clara Rosa Levina Oudenaarden, Martin Jakob Larsen, Philip Ahle Erichsen, Christian Bonde Pedersen, Frantz Rom Poulsen, Peter Grupe, Torben A Kruse, Mads Thomassen, Bjarne Winther Kristensen, Multiomic profiling of glioblastoma metabolic lesions reveals complex intratumoral genomic evolution and dipeptidase-1-driven vascular proliferation, *Neuro-Oncology*, Volume 27, Issue 10, October 2025, Pages 2547–2563, https://doi.org/10.1093/neuonc/noaf071

The following change has been made to the originally published manuscript.

A statement has been added to the ‘Funding’ section that reads:

Clara Oudenaarden is funded by the European Union’s Horizon 2020 research and innovation program under the Marie Sklodowska-Curie grant agreement No. 945322 (LEAD).

