# Supplementary material for: Multiomic profiling of glioblastoma metabolic lesions reveals complex intratumoral genomic evolution and dipeptidase-1-driven vascular proliferation
Source: Neuro Oncol. 2025 May 4;27(10):2547–63. doi: 10.1093/neuonc/noaf071 (PMC12833548; doi:10.1093/neuonc/noaf071)
Supplement: noaf071_Supplementary_Tables_S1-S4_Figures_1-S13 [file noaf071_supplementary_tables_s1-s4_figures_1-s13.zip › N-O-D-24-00556R1 Supplementary figure legend 1 to 13.docx]

# Supplementary Figure legends:

**Supplementary Fig.S1:**

**(A)** Tumor mutational allele frequency analysis by whole-exome sequencing in autologous kryo (hypometabolic tumor lesion) on the x-axis and zesto (hypermetabolic tumor lesion) on the y-axis from two glioblastoma patients with low tumor content in kryo. Zesto and kryo-specific variants are marked respectively with red and blue. Data points represent mutations, and the red dotted line represents perfect uniformity.

**Table 1.** Shows the demographics of glioblastoma multiforme (GBM) patients involved in the study.

**(B)** Total number of cells (measured by DAPI staining) in different metabolic lesions.

**Supplementary Fig.S2:** MRI, [^18^F] FDG-PET, [^11^C] MET-PET, and co-registration images from all glioblastoma patients showing the location of the hypometabolic (blue circle), hypermetabolic (red circle), and akri (green circle) lesions. Blue intensity represents the lowest, and red represents the highest accumulation of radiotracers. The metabolic lesion regions indicated in this image are represented in two dimensions. However, please note that these lesions are distributed in three-dimensional space and may not reside within the same plane. The circled areas represent only an approximate two-dimensional projection.

# Supplementary Fig.S3: Spatial genomic heterogeneity in autologous lesion in glioblastoma

1. Merged transverse MRI, concordant FDG-PET, and MET-PET images exhibiting different geo locations of lesions and textboxes represent key lesion-specific chromosomal alterations in the patients.
2. A connectivity map of the significant protein-protein interaction involving genes from zesto lesion-specific CNVs from all six glioblastoma patients. 9 distinct functional clusters (illustrated in a discrete color) were detected, and disconnected nodes were excluded from the map.

# Supplementary Fig.S4: Single-cell transcriptomic profiling highlights proliferating tumor cell clusters and co-expression of hypermetabolic lesion-specific genes involved in CNVs events.

1. Uniform Manifold Approximation and Projection (UMAP) plot of the location-averaged transcriptome for all cells (5604 cells) from a glioblastoma patient (male 57 years) obtained from 10x Genomics. Different cell types are colored by cluster (top left), the proliferative tumor cluster (based on MKI67 expression) is marked in the UMAP plot, and the top 10 highly expressed genes in the proliferative tumor cluster.
2. The expression of genes (*DLGPA3, NMU, PAICS, CEP135, PPAT,* among other genes) involved in zesto lesion-specific CNV events shows a cellular colocalization with proliferative tumor cell gene signature.
3. mRNA expression and copy number alteration of zesto lesion-specific cancer driver genes in the glioblastoma and low-grade glioma samples from the TCGA dataset (Homdel: Homozygous deletion, HetLoss: Loss of heterozygosity).
4. Overall patient survival analysis of glioblastoma (163 patients) and low-grade glioma (518 patients) based on the high and low expression of zesto lesion-specific signature genes. Signature genes are mentioned in the lower left corner of plots, data analyzed by the GEPIA-2 web tool.

**Supplementary Fig.S5:** The hypermetabolic (zesto) lesion exhibits micro- amplification and deletion of the chromosomal region that could potentially enhance tumor aggressiveness.

1. Global copy number variation in autologous lesions from glioblastoma patients (2,3,4, 5 and 6) as identified using the whole exome sequencing workflows by the Varseq analysis tool. The x-axis depicts the genomic location, and the Y-axis indicates the log ratio of copy number. Arrows indicating lesion-specific copy number alterations.
2. mRNA expression of genes located in the amplicon of patient-2, Chromosome-9p, marked with a red arrow.
3. mRNA expression of genes located in the deleted region of the zesto lesion (Patient#2, Chromosome#9p, marked with red arrow), genes are arranged in the chromosomal order to show the pattern of CNV and gene expression difference between autologous zesto, kryo, and akri lesions.
4. mRNA expression of *CDKN2A*, *CDKN2B*, and *MTAP* gene located in the zesto's deleted region (Patient#5, Chromosome-9p, marked with red arrow).

**Supplementary Fig.S6:** Hypermetabolic tumor lesions have higher genomic instabilities: Circos plots of the genome of autologous lesions from different glioblastoma patients showing genomic rearrangements and chromothripsis (marked with a red arrow in zesto lesions from patients 3 and 6). The outer ring depicts the SNVs from the whole exome sequencing analysis. The middle ring indicates the copy number variation from whole exome sequencing, the red color indicates the gain, the green indicates the loss of chromosomes, and the blue shows heterozygosity. The innermost circle shows the structural variation analyzed by whole genome sequencing. The red arrow marks chromothripsis events, and chromoplexy events are marked by a dark grey arrow.

# Supplementary Fig.S7: Hypermetabolic tumor lesions have higher genomic instabilities:

1. Circos plots of the genome from the zesto, kryo, and akri from patient 6. The outer ring depicts the SNVs from the whole genome sequencing analysis.
2. Substitution plots in the respective lesions, indicating the highest substitution events were found in zesto compared to kryo and akri, C to T substitution was the most dominant and followed by T to C substitutions.
3. Bar plots showing the quantification of deletion, insertions, and global rearrangements in different lesions from whole genome sequencing analysis.

# Supplementary Fig.S8: Hypermetabolic tumor lesions exhibit a greater degree of genomic instability.

A global overview of B- allele frequency analysis on all 29 samples to highlight the major occurrence of a CNV, such as a deletion, duplication, or loss of heterozygosity (LOH), in the whole genome region.

# Supplementary Fig.S9: *DPEP1* a novel marker for hypermetabolic lesion of glioblastoma

1. mRNA expression of *DPEP1* in the CGGA-GBM-LGG dataset shows higher expression in glioblastoma compared to other low-grade gliomas and in IDH mutant vs wild type gliomas**.**
2. mRNA expression (log2) of the Dipeptidase 1 gene (*DPEP1*) in the TCGA-GBM database is depicted, contrasting non-tumour brain tissue. Statistical significance (P<0.01) was analyzed by one-way analysis of variance (ANOVA) and is indicated with an asterisk.
3. A connectivity map illustrating the significant proteins from the glutathione metabolism pathway that interact with the dipeptidase 1 enzyme. The colours of the nodes are depicted in the key provided below.
4. Kaplan-Meier plot illustrates overall glioblastoma survival distributions of patients, stratified and colored by high and low expression of *DPEP1* from CGGA (n=220), Rembrandt dataset (n=88), and Korean SMC (n=57) datasets. Data were analyzed using the GlioVis web tool on glioblastoma patients.
5. **and (F)** UMAP of 143,793 nuclei shows the expression pattern of DPEP1 in normal brain cells, venous cell clusters show the highest expression of *DPEP1* gene. (https://twc- stanford.shinyapps.io/human_bbb).
6. Representative confocal microscopy images depict the colocalization of dipeptidase 1-positive cells (in blue to orange) and CD34-positive cells (in red) in the glioblastoma tissue section and normal brain.
7. Kaplan-Meier plot illustrates overall glioblastoma survival distributions of patients, stratified and colored by high and low expression of *CD34* from CGGA (n=220), Rembrandt dataset (n=88) datasets. Data were analyzed using the GlioVis web tool.
8. brightfield image depicting the endothelial sprout assay with human glioblastoma derived endothelial cells treated with DMSO and cilastatin, The images were captured at 10x magnification, with a scale bar of 50µm

**(J)** Immunofluorescence images (in inverted color) depicting the angiogenesis assay with human umbilical vein endothelial cells (HUVECs) treated with DMSO and cilastatin, and stained with Hoechst 33342. The images were captured at 20x magnification, with a scale bar of 100µm.

**(K)** Bar plot illustrating the effect of dipeptidase 1 inhibitor on angiogenesis assay, measured by the number of angiogenic nodes, tubes, and meshes, respectively. Control HUVECs cellline were treated with DMSO, while 10µM of cilastatin was utilized. The indicated p-value is derived from a paired t-test.**Bottom of Form**

# Supplementary Fig.S10: Representative immunohistochemistry images show dipeptidase 1 expression in human tissues:

1. Representative image showing DPEP1 staining in positive control tissues, including kidney, testis, and pancreas.
2. Representative image showing DPEP1 staining in samples of human glioblastoma, normal brain, and intestine. Magnified areas are displayed in the bottom panel with a 50 µm scale bar, while the top panel has a 500 µm scale bar.
3. Representative CD34 staining illustrates vascular structure in a normal human brain and glioblastoma.
4. Immunohistochemistry images depicting a longitudinal (left) and horizontal (right) section of a microvessel within a glioblastoma tumor. DPEP1 expression is exclusively localized to the endothelial tip and vascular sprouts of hypermetabolic tumor lesions and not in mature vessels.
5. Quantification of CD34 in zesto, kryo, and akri micro lesions, the violin plot indicates the positive fraction per lesion. The indicated p-value is derived from a paired t-test.
6. Representative IHC images and quantification of IBA1 in zesto, kryo, and akri micro lesions.
7. mRNA expression (log2) of the *AIF1* and *CD34* was examined in the kryo, zesto, and akri lesions. Statistical differences in mRNA expression were evaluated using a two-tailed t-test.
8. mRNA expression (log2) of major neutrophils related gene markers was examined in the kryo, zesto, and akri lesions. Statistical differences in mRNA expression were evaluated using a two-tailed t-test.
9. Representative image of CD34-positive glioblastoma-derived cells treated with DMSO or Cilastatin for 96 hours, showing cellular health and absence of toxicity with the DPEP1 inhibitor.

**Supplementary Fig.S11:**

1. The mRNA expression (log₂) of genes associated with vascular tip GBM stem cells, pericytes, and immune cells, highlighting the spatial distribution and enrichment within DPEP1-positive lesions.
2. Immunofluorescence staining of DLL-4 (green) and DPEP1 (red) in a representative glioblastoma sections, depicting the colocalization of the endothelial tip cell marker DLL-4 with DPEP1.

**Supplementary Fig.S12:**

Bar plot showing mRNA expression of key genes (upregulated in Zesto (red) or Kryo (blue) lesions) in glioblastoma patients (CGGA-GBM), comparing treatment status (1 = treated with chemo- and radiotherapy; 0 = untreated). P-values were calculated using a t-test. Data analyzed using Gliovis webportal.

**Supplementary Fig.S13:**

Graphical overview and comparison of the kryo, zesto, and akri lesions of glioblastoma patients:

The innermost circle (in the round) shows single nucleotide variant analysis from whole exome sequencing data representing the top six most frequently mutated genes (*TERT, TP53, FLG, SYN1, TTN,* and *HMCN1*). The second circle (in the square) indicates the mRNA expression heatmap of putative tumor suppressor-like genes (*MTUS2, RTN1, PDIA2,* and *CRLF1*) in different lesion groups. The third circle (in the triangle) indicates the copy number alterations from whole exome sequencing analysis of representative genes (*NDUFA4, CYCS, HK2,* and *MMADHC*) from oxidative phosphorylation, gluconeogenesis, and amino acid metabolism pathways. The fourth circle (rounded rectangle) indicates the patient number. The stacked bar plot shows the number of structural variants based on whole-genome sequencing analysis. Abbreviations: (AMP) amplification, gain, (HETD) heterozygous deletion, (HLAMP) high-level amplifications, (NLOH) Neutral Loss of heterozygosity. Outer petals in blue shades (hypometabolic lesions: kryo), red shades (hypermetabolic lesions: zesto), and green shades (no metabolic activity lesions: akri) indicate the hallmarks of metabolic lesions. Inner petals in light blue, light red, and light green represent the kryo, zesto, and akri lesions sections.
