# Supplementary material for: Multiomic profiling of glioblastoma metabolic lesions reveals complex intratumoral genomic evolution and dipeptidase-1-driven vascular proliferation
Source: Neuro Oncol. 2025 May 4;27(10):2547–63. doi: 10.1093/neuonc/noaf071 (PMC12833548; doi:10.1093/neuonc/noaf071)
Supplement: noaf071_Supplementary_Tables_S1-S4_Figures_1-S13 [file noaf071_supplementary_tables_s1-s4_figures_1-s13.zip › N-O-D-24-00556R2 Supplementary Methods Changes saved.docx]

**Supplementary Methods**

**Whole Exome sequencing data analysis**

For exome sequencing, 1000ng of DNA was prepared using the KAPA Target Enrichment Kit (Roche). Raw reads were aligned and duplicate-marked using the DRAGEN Bio-IT Platform (Illumina). Somatic variant calling was performed with VarScan v. 2.3.4, considering only bases with a quality score of Q20 or higher. Variant annotation and filtration were done using VarSeq (Golden Helix), with criteria including genotype quality (QC) >100, read depth >100x, VAF >5% in tumor samples, and VAF <2% with allele count <3 in normal samples. CNV calling, filtering, and visualization were also performed using VarSeq with criteria: number of exons >5 and average coverage >100x. Sequencing was done in a single run, using one NovaSeq S4 lane for RNA-Seq, two lanes for exomes, and one lane for genome sequencing (2*150bp).

**Tumor content analysis**

Tumor content was estimated using CNV tools: TitanCNA (WGS and WES), AscatNGS (WGS and WES), Sequenza (WES), and Battenberg (WGS) with default settings. Additionally, variant allele frequencies (VAFs) from WES data were plotted against VAFs from another sample to identify the center of heterozygous variants, ideally representing half of the tumor content in each sample.

**Hematoxylin and Eosin, DPEP1, DLL4, CD34 staining**

A portion of the biopsies was formaldehyde-fixed, paraffin-embedded, and cut into 3µm sections. Hematoxylin and Eosin staining visualized cellularity. For immunohistochemistry, Dipeptidase 1 antibody (Sigma #HPA012783) or CD34 (Invitrogen QBEND/10), DLL-4 (R&D Systems MAB1389-SP) was diluted at 1:100, incubated with tissue sections for 2 hours, followed by washing and secondary antibody application. Detection used a chromogen substrate, with optional hematoxylin or DAPI counterstaining for cellular/nuclear visualization. Slides were dehydrated, mounted, and examined microscopically. The stained sections were scanned with a NanoZoomer 2.0-HT slide scanner and analyzed using Visiopharm software. **Note on the choice of CD34 as an endothelial marker:** We selected CD34 for our colocalization study of microvascular proliferation in glioblastoma immunohistochemistry analysis due to its well-established role in highlighting endothelial progenitor cells and newly forming microvessels. Additionally, CD34 is widely used in automated pathological processing, ensuring consistency, reproducibility, and clear visualization of vascular structures in our analysis.

**RNA Sequencing**

mRNA libraries were created from 10 ng of RNA per sample using TruSeq® RNA Library Prep and Enrichment kits. Sequencing on an Illumina NovaSeq 6000 System (2x150bp) with one S4 flow cell lane for 23 biopsies yielded 2,568,575,326 reads. STAR aligned reads to the human genome (GRCh38), with duplicates removed and sorted by SamTools. Gene expression counts were generated with featureCounts, normalized to CPM, and TMM normalization was applied with edgeR. Low CPM genes were filtered, and about 75% of reads were uniquely mapped. Fusion events were cross-validated using the STAR-Fusion tool with RNA-seq data.

**Mutation burden analysis**

SNV and indel burden analyses were performed using the formula for the number of SNVs identified in the WES data by the Varscan tool and divided by the total area of the exome analyzed.

**Endothelial cell tube formation assay**

20,000 HUVEC cells were cultured on geltrex-coated coverslips in a 24-well plate with control (DMSO) and without cilastatin. Cells were stained with Hoechst 33342 and imaged at 20X magnification. Fiji software analyzed angiogenesis parameters like tube formation, nodes, and branching points. Multiple fields of view were analyzed per sample, and data were compiled for statistical analysis.

**Aortic ring assay**

Thoracic aortae were harvested from 8-week-old C57BL/6 mice, cleaned, and sectioned into rings following a previously established protocol (Baker et al., Nature Protocols, 2012, DOI: 10.1038/nprot.2011.435). The rings were plated in Geltrex-coated 48-well plates and serum-starved overnight. Subsequently, the rings were treated every two days for seven days with either DMSO or cilastatin. Sprouting was assessed using brightfield microscopy, and the sprout area was measured using the Celigo image cytometer. Microvessel sprouts were analyzed for their number and total area.

**Glioblastoma tumor processing and purification of endothelial cells for sprout assay**

Whole-tumor specimens were collected in saline, washed twice in sterile phosphate-buffered saline (PBS) containing penicillin and streptomycin, and minced into fragments smaller than 5 mm. Tissue homogenization was performed following the Brain Tumor Dissociation Kit (P) protocol (Miltenyi Biotech, 130-095-942). Enzymatically digested and mechanically dissociated tissues (using the gentleMACS™ Dissociator, 130-093-235) were filtered through a 70-μm strainer (Miltenyi, 130-098-463) to remove debris. Red blood cells were lysed (Santa Cruz, SC296258), and additional debris was removed using a density gradient (Miltenyi Biotech, 130-109-398).

Samples were washed again in sterile PBS, and endothelial cells were magnetically sorted using a CD34 antibody (Invitrogen QBEND/10) for 15 minutes, followed by incubation with microbead-conjugated secondery antibody for 10 minutes. The cell suspensions were washed and purified by passing through LS columns (Miltenyi Biotech, 130-042-401). Purified endothelial cells were counted, stained with CD34 monoclonal antibody, and analyzed by FACS to confirm purity. 30,000 cells were then used for droplet or spheroid formation and sprouting experiments using Endothelial Cell Growth Medium (Cat. No. C-22010).

Endothelial spheroids or droplets were plated in Geltrex™-coated 48-well plates, with the coating polymerized at 37°C for 30 minutes. Endothelial cell growth medium supplemented with angiogenic factors cocktail, including VEGF, was used. The plates were incubated at 37°C, and sprouting was monitored over 7 days. DMSO or cilastatin was added every 48 hours, with the medium refreshed every 2 days. Sprout area were assessed using brightfield microscopy or analyzed with Fiji software for quantitative measurements.

**Data availability**

Whole-genome and exome sequencing data are identity-sensitive and not publicly available per Danish legislation. Researchers can request access to raw genomic data by contacting at Bjarne.winther.kristensen.01@regionh.dk, under an agreement to protect individual confidentiality.
