# Supplementary material for: Multiomic profiling of glioblastoma metabolic lesions reveals complex intratumoral genomic evolution and dipeptidase-1-driven vascular proliferation
Source: Neuro Oncol. 2025 May 4;27(10):2547–63. doi: 10.1093/neuonc/noaf071 (PMC12833548; doi:10.1093/neuonc/noaf071)
Supplement: noaf071_Supplementary_Tables_S1-S4_Figures_1-S13 [file noaf071_supplementary_tables_s1-s4_figures_1-s13.zip › Table S1.docx]

**Table S1. Demographics of GBM patients**

| **Patient number** | **Age** | **Sex** | **Diagnosis** | **IDH** | **MGMT**  **Methylation** | **WGS samples** | **WES samples** | **Transcriptome samples** | **Prior Treatments** |
| --- | --- | --- | --- | --- | --- | --- | --- | --- | --- |
| 1 | 68 | M | GBM | WT | unmethylated | 4 | 5 | 4 | NO |
| 2 | 74 | M | GBM | WT | methylated | 3 | 4 | 3 | NO |
| 3 | 48 | M | GBM | WT | methylated | 4 | 5 | 4 | NO |
| 4 | 76 | M | GBM | WT | unmethylated | 4 | 5 | 4 | NO |
| 5 | 72 | F | GBM | WT | unmethylated | 4 | 5 | 4 | NO |
| 6 | 72 | M | GBM | WT | methylated | 4 | 5 | 4 | NO |
