# Supplementary material for: Multiomic profiling of glioblastoma metabolic lesions reveals complex intratumoral genomic evolution and dipeptidase-1-driven vascular proliferation
Source: Neuro Oncol. 2025 May 4;27(10):2547–63. doi: 10.1093/neuonc/noaf071 (PMC12833548; doi:10.1093/neuonc/noaf071)
Supplement: noaf071_Supplementary_Tables_S1-S4_Figures_1-S13 [file noaf071_supplementary_tables_s1-s4_figures_1-s13.zip › Table S2.docx]

**CNV all samples**

Blood GBM-1

| **Region** | **# Targets** | **# Samples** | **CNV State** | **Gene Names** | **# Genes** |
| --- | --- | --- | --- | --- | --- |
| [1:12952747-13745175](genomebrowse://api/zoom?locus=1:12952747-13745175) | 41 | 4 | Duplicate | HNRNPCL2,PRAMEF5,PRAMEF6,PRAMEF7,PRAMEF8,PRAMEF10,PRAMEF13,PRAMEF14,PRAMEF15,PRAMEF17,PRAMEF18,PRAMEF19,PRAMEF20,PRAMEF25,PRAMEF33P | 15 |
| [1:145303910-145315899](genomebrowse://api/zoom?locus=1:145303910-145315899) | 11 | 1 | Duplicate | NBPF10 | 1 |
| [2:32639837-33246273](genomebrowse://api/zoom?locus=2:32639837-33246273) | 88 | 3 | Duplicate | BIRC6,LTBP1,TTC27 | 3 |
| [2:96561147-96574322](genomebrowse://api/zoom?locus=2:96561147-96574322) | 15 | 3 | Duplicate | ANKRD36C | 1 |
| [6:31994229-31997575](genomebrowse://api/zoom?locus=6:31994229-31997575) | 12 | 1 | Duplicate | C4B | 1 |
| [6:32009126-32012493](genomebrowse://api/zoom?locus=6:32009126-32012493) | 12 | 5 | Duplicate | CYP21A2,TNXB | 2 |
| [7:142459625-142494975](genomebrowse://api/zoom?locus=7:142459625-142494975) | 11 | 1 | Duplicate | PRSS1 | 1 |
| [8:11967409-12293852](genomebrowse://api/zoom?locus=8:11967409-12293852) | 23 | 3 | Duplicate | DEFB130,FAM86B1,FAM86B2,USP17L2,USP17L7,ZNF705D | 6 |
| [15:30375256-30696552](genomebrowse://api/zoom?locus=15:30375256-30696552) | 30 | 1 | Duplicate | CHRFAM7A,GOLGA8J,GOLGA8R | 3 |
| [15:74363251-74373076](genomebrowse://api/zoom?locus=15:74363251-74373076) | 17 | 3 | Duplicate | GOLGA6A | 1 |
| [15:75555689-75586816](genomebrowse://api/zoom?locus=15:75555689-75586816) | 33 | 2 | Duplicate | GOLGA6C,GOLGA6D | 2 |
| [16:14782022-15044112](genomebrowse://api/zoom?locus=16:14782022-15044112) | 57 | 1 | Duplicate | NOMO1,NPIPA1,NPIPA2,NPIPA3,PLA2G10 | 5 |
| [16:18414874-18794424](genomebrowse://api/zoom?locus=16:18414874-18794424) | 38 | 2 | Duplicate | NOMO2,NPIPA8,RPS15A | 3 |
| [16:29397116-29473271](genomebrowse://api/zoom?locus=16:29397116-29473271) | 20 | 1 | Duplicate | BOLA2,BOLA2-SMG1P6,NPIPB11,SLX1B,SULT1A4 | 5 |
| [X:134873998-134967496](genomebrowse://api/zoom?locus=X:134873998-134967496) | 17 | 5 | Duplicate | CT45A3,CT45A5,CT45A8,CT45A9,CT45A10 | 5 |

Lesion: A1

| **Region** | **# Targets** | **# Samples** | **CNV State** | **Gene Names** | **# Genes** |
| --- | --- | --- | --- | --- | --- |
| [1:12952747-13745175](genomebrowse://api/zoom?locus=1:12952747-13745175) | 41 | 4 | Duplicate | HNRNPCL2,PRAMEF5,PRAMEF6,PRAMEF7,PRAMEF8,PRAMEF10,PRAMEF13,PRAMEF14,PRAMEF15,PRAMEF17,PRAMEF18,PRAMEF19,PRAMEF20,PRAMEF25,PRAMEF33P | 15 |
| [1:145303910-145315899](genomebrowse://api/zoom?locus=1:145303910-145315899) | 11 | 1 | Duplicate | NBPF10 | 1 |
| [2:32639837-33246273](genomebrowse://api/zoom?locus=2:32639837-33246273) | 88 | 3 | Duplicate | BIRC6,LTBP1,TTC27 | 3 |
| [2:96561147-96574322](genomebrowse://api/zoom?locus=2:96561147-96574322) | 15 | 3 | Duplicate | ANKRD36C | 1 |
| [6:31994229-31997575](genomebrowse://api/zoom?locus=6:31994229-31997575) | 12 | 1 | Duplicate | C4B | 1 |
| [6:32009126-32012493](genomebrowse://api/zoom?locus=6:32009126-32012493) | 12 | 5 | Duplicate | CYP21A2,TNXB | 2 |
| [7:142459625-142494975](genomebrowse://api/zoom?locus=7:142459625-142494975) | 11 | 1 | Duplicate | PRSS1 | 1 |
| [8:11967409-12293852](genomebrowse://api/zoom?locus=8:11967409-12293852) | 23 | 3 | Duplicate | DEFB130,FAM86B1,FAM86B2,USP17L2,USP17L7,ZNF705D | 6 |
| [15:30375256-30696552](genomebrowse://api/zoom?locus=15:30375256-30696552) | 30 | 1 | Duplicate | CHRFAM7A,GOLGA8J,GOLGA8R | 3 |
| [15:74363251-74373076](genomebrowse://api/zoom?locus=15:74363251-74373076) | 17 | 3 | Duplicate | GOLGA6A | 1 |
| [15:75555689-75586816](genomebrowse://api/zoom?locus=15:75555689-75586816) | 33 | 2 | Duplicate | GOLGA6C,GOLGA6D | 2 |
| [16:14782022-15044112](genomebrowse://api/zoom?locus=16:14782022-15044112) | 57 | 1 | Duplicate | NOMO1,NPIPA1,NPIPA2,NPIPA3,PLA2G10 | 5 |
| [16:18414874-18794424](genomebrowse://api/zoom?locus=16:18414874-18794424) | 38 | 2 | Duplicate | NOMO2,NPIPA8,RPS15A | 3 |
| [16:29397116-29473271](genomebrowse://api/zoom?locus=16:29397116-29473271) | 20 | 1 | Duplicate | BOLA2,BOLA2-SMG1P6,NPIPB11,SLX1B,SULT1A4 | 5 |
| [X:134873998-134967496](genomebrowse://api/zoom?locus=X:134873998-134967496) | 17 | 5 | Duplicate | CT45A3,CT45A5,CT45A8,CT45A9,CT45A10 | 5 |

Lesion: B1

| **Region** | **# Targets** | **# Samples** | **CNV State** | **genes** | **Flags** |
| --- | --- | --- | --- | --- | --- |
| [2:152437997-152465190](genomebrowse://api/zoom?locus=2:152437997-152465190) | 22 | 4 | Duplicate | NEB | 1 |
| [4:54280782-55987357](genomebrowse://api/zoom?locus=4:54280782-55987357) | 103 | 1 | Duplicate | CHIC2,FIP1L1,GSX2,KDR,KIT,LNX1,PDGFRA | 7 |
| [5:147774340-149003703](genomebrowse://api/zoom?locus=5:147774340-149003703) | 127 | 2 | Het Deletion | ABLIM3,ADRB2,AFAP1L1,ARHGEF37,CSNK1A1,FBXO38,GRPEL2,HTR4,IL17B,PCYOX1L,SH3TC2 | 11 |
| [5:176830482-176895900](genomebrowse://api/zoom?locus=5:176830482-176895900) | 43 | 2 | Duplicate | DBN1,F12,GRK6,PRR7 | 4 |
| [9:67926873-70919121](genomebrowse://api/zoom?locus=9:67926873-70919121) | 79 | 2 | Duplicate | ANKRD20A1,ANKRD20A4,CBWD3,CBWD5,CBWD6,FOXD4L3,FOXD4L4,FOXD4L5,FOXD4L6 | 9 |
| [10:92997-135379033](genomebrowse://api/zoom?locus=10:92997-135379033) | 8110 | 1 | Het Deletion | A1CF,ABCC2,ABI1,ABLIM1,ACADSB,ACBD5,ACBD7,ACSL5,ACSM6,ACTA2,ACTR1A,ADAM8,ADAM12,ADAMTS14,ADARB2,ADD3,ADGRA1,ADIRF,ADK,ADO,ADRA2A,ADRB1,AFAP1L2,AGAP4,AGAP5,AGAP6,AGAP9,AGAP11,AIFM2,AKR1C1,AKR1C2,AKR1C3,AKR1C4,AKR1E2,ALDH18A1,ALOX5,ANAPC16,ANK3,ANKRD1,ANKRD2,ANKRD16,ANKRD22,ANKRD26,ANKRD30A,ANTXRL,ANXA7,ANXA8,ANXA8L1,ANXA11,AP3M1,APBB1IP,ARHGAP12,ARHGAP19,ARHGAP21,ARHGAP22,ARID5B,ARL3,ARL5B,ARMC3,ARMC4,ARMS2,AS3MT,ASAH2,ASAH2B,ASB13,ASCC1,ATAD1,ATE1,ATOH7,ATP5C1,ATRNL1,AVPI1,BAG3,BAMBI,BBIP1,BCCIP,BEND7,BICC1,BLNK,BLOC1S2,BMI1,BMPR1A,BMS1,BNIP3,BORCS7,BTAF1,BTBD16,BTRC,BUB3,C1QL3,C10orf10,C10orf11,C10orf25,C10orf35,C10orf53,C10orf55,C10orf62,C10orf67,C10orf71,C10orf76,C10orf82,C10orf88,C10orf90,C10orf91,C10orf95,C10orf99,C10orf105,C10orf107,C10orf111,C10orf113,C10orf120,C10orf126,C10orf128,C10orf131,C10orf142,CACNB2,CACUL1,CALHM1,CALHM2,CALHM3,CALML3,CALML5,CALY,CAMK1D,CAMK2G,CASC10,CASP7,CC2D2B,CCAR1,CCDC3,CCDC6,CCDC7,CCDC172,CCDC186,CCNJ,CCNY,CCSER2,CDC123,CDH23,CDHR1,CDK1,CDNF,CELF2,CEP55,CFAP43,CFAP46,CFAP58,CFAP70,CH25H,CHAT,CHCHD1,CHST3,CHST15,CHUK,CISD1,CLRN3,CNNM1,CNNM2,COL13A1,COL17A1,COMMD3,COMMD3-BMI1,COMTD1,COX15,CPEB3,CPN1,CPXM2,CREM,CRTAC1,CSGALNACT2,CSTF2T,CTBP2,CTNNA3,CUBN,CUEDC2,CUL2,CUTC,CUZD1,CWF19L1,CXCL12,CYP2C8,CYP2C9,CYP2C18,CYP2C19,CYP2E1,CYP17A1,CYP26A1,CYP26C1,DCLRE1A,DCLRE1C,DDIT4,DDX21,DDX50,DHTKD1,DHX32,DIP2C,DKK1,DLG5,DMBT1,DNA2,DNAJB12,DNAJC1,DNAJC9,DNAJC12,DNMBP,DNTT,DOCK1,DPCD,DPYSL4,DRGX,DUPD1,DUSP5,DUSP13,DYDC1,DYDC2,EBF3,EBLN1,ECD,ECHDC3,ECHS1,EDRF1,EGR2,EIF3A,EIF4EBP2,EIF5AL1,ELOVL3,EMX2,ENKUR,ENO4,ENTPD1,ENTPD7,EPC1,ERCC6,ERLIN1,EXOC6,EXOSC1,FAM13C,FAM24A,FAM24B,FAM25A,FAM25C,FAM25G,FAM35A,FAM45A,FAM53B,FAM107B,FAM149B1,FAM160B1,FAM170B,FAM171A1,FAM175B,FAM188A,FAM196A,FAM204A,FAM208B,FAM213A,FANK1,FAS,FBXL15,FBXO18,FBXW4,FFAR4,FGF8,FGFBP3,FGFR2,FOXI2,FRA10AC1,FRAT1,FRAT2,FRMD4A,FRMPD2,FUOM,FUT11,FXYD4,FZD8,GAD2,GATA3,GBF1,GDF2,GDF10,GDI2,GFRA1,GHITM,GJD4,GLRX3,GLUD1,GOLGA7B,GOT1,GPAM,GPR26,GPR158,GPRIN2,GRID1,GRK5,GSTO1,GSTO2,GTPBP4,H2AFY2,HABP2,HACD1,HECTD2,HELLS,HERC4,HHEX,HIF1AN,HK1,HKDC1,HMX2,HMX3,HNRNPF,HNRNPH3,HOGA1,HPS1,HPS6,HPSE2,HSPA12A,HSPA14,HTR7,HTRA1,IDE,IDI1,IDI2,IFIT1,IFIT1B,IFIT2,IFIT3,IFIT5,IKZF5,IL2RA,IL15RA,INA,INPP5A,INPP5F,IPMK,ITGA8,ITGB1,ITIH2,ITIH5,ITPRIP,JAKMIP3,JMJD1C,KAT6B,KAZALD1,KCNIP2,KCNK18,KCNMA1,KIAA1217,KIAA1462,KIF1BP,KIF5B,KIF11,KIF20B,KIN,KLF6,KLLN,KNDC1,LARP4B,LBX1,LCOR,LDB1,LDB3,LGI1,LHPP,LIPA,LIPF,LIPJ,LIPK,LIPM,LIPN,LOXL4,LRIT1,LRIT2,LRRC18,LRRC20,LRRC27,LRRTM3,LYZL1,LYZL2,LZTS2,MALRD1,MAP3K8,MAPK8,MARCH5,MARCH8,MARVELD1,MASTL,MAT1A,MBL2,MCM10,MCMBP,MCU,MEIG1,METTL10,MFSD13A,MGEA5,MGMT,MICU1,MINPP1,MKI67,MKX,MLLT10,MMP21,MMRN2,MMS19,MORN4,MPP7,MRC1,MRLN,MRPL43,MRPS16,MSMB,MSRB2,MSS51,MTG1,MTPAP,MTRNR2L5,MTRNR2L7,MXI1,MYO3A,MYOF,MYOZ1,MYPN,NANOS1,NCOA4,NDST2,NDUFB8,NEBL,NET1,NEURL1,NEUROG3,NFKB2,NHLRC2,NKX1-2,NKX2-3,NKX6-2,NMT2,NOC3L,NODAL,NOLC1,NPFFR1,NPM3,NPS,NPY4R,NRAP,NRBF2,NRG3,NRP1,NSMCE4A,NSUN6,NT5C2,NUDT5,NUDT13,NUTM2A,NUTM2B,OAT,OGDHL,OIT3,OLAH,OPALIN,OPN4,OPTN,OR13A1,OTUD1,P4HA1,PALD1,PANK1,PAOX,PAPSS2,PARD3,PARG,PAX2,PBLD,PCBD1,PCDH15,PCGF5,PCGF6,PDCD4,PDCD11,PDE6C,PDLIM1,PDSS1,PDZD7,PDZD8,PFKFB3,PFKP,PGAM1,PGBD3,PHYH,PHYHIPL,PI4K2A,PIK3AP1,PIP4K2A,PITRM1,PITX3,PKD2L1,PLA2G12B,PLAC9,PLAU,PLCE1,PLEKHA1,PLEKHS1,PLPP4,PLXDC2,PNLIP,PNLIPRP1,PNLIPRP2,PNLIPRP3,POLL,POLR3A,PPA1,PPIF,PPP1R3C,PPP2R2D,PPP3CB,PPRC1,PRAP1,PRDX3,PRF1,PRKCQ,PRKG1,PRLHR,PROSER2,PRPF18,PRTFDC1,PSAP,PSD,PSTK,PTCHD3,PTEN,PTER,PTF1A,PTPN20,PTPRE,PWWP2B,PYROXD2,R3HCC1L,RAB11FIP2,RAB18,RASGEF1A,RASSF4,RBM17,RBM20,RBP3,RBP4,REEP3,RET,RGR,RGS10,RHOBTB1,RNLS,RPEL1,RPP30,RPP38,RPS24,RRP12,RSU1,RTKN2,RUFY2,SAMD8,SAR1A,SCD,SEC23IP,SEC24C,SEC31B,SEC61A2,SEMA4G,SEPHS1,SFMBT2,SFR1,SFRP5,SFTPA1,SFTPA2,SFTPD,SFXN2,SFXN3,SFXN4,SGMS1,SGPL1,SH2D4B,SH3PXD2A,SHOC2,SHTN1,SIRT1,SKIDA1,SLC16A9,SLC16A12,SLC18A2,SLC18A3,SLC25A16,SLC25A28,SLC29A3,SLC35G1,SLC39A12,SLF2,SLIT1,SLK,SMC3,SMNDC1,SNCG,SORBS1,SORCS1,SORCS3,SPAG6,SPOCK2,SPRN,SRGN,ST8SIA6,STAM,STAMBPL1,STK32C,STN1,STOX1,SUFU,SUPV3L1,SUV39H2,SVIL,SYCE1,SYNPO2L,SYT15,TACC2,TACR2,TAF3,TAF5,TBATA,TBC1D12,TCERG1L,TCF7L2,TCTN3,TDRD1,TECTB,TET1,TEX36,TFAM,THNSL1,TIAL1,TIMM23,TIMM23B,TLL2,TLX1,TM9SF3,TMEM26,TMEM72,TMEM236,TMEM254,TNKS2,TRDMT1,TRIM8,TRUB1,TSPAN14,TSPAN15,TUBAL3,TUBB8,TUBGCP2,TWNK,TYSND1,UBE2D1,UBTD1,UCMA,UCN3,UNC5B,UPF2,UROS,USMG5,USP6NL,USP54,UTF1,VAX1,VCL,VDAC2,VENTX,VIM,VPS26A,VSIR,VSTM4,VTI1A,VWA2,WAC,WAPL,WASHC2A,WASHC2C,WBP1L,WDFY4,WDR11,WDR37,WNT8B,XPNPEP1,YME1L1,ZCCHC24,ZDHHC6,ZDHHC16,ZEB1,ZFAND4,ZFYVE27,ZMIZ1,ZMYND11,ZNF22,ZNF25,ZNF32,ZNF33A,ZNF33B,ZNF37A,ZNF239,ZNF248,ZNF365,ZNF438,ZNF485,ZNF488,ZNF503,ZNF511,ZNF518A,ZRANB1,ZSWIM8,ZWINT | 713 |
| [14:20443678-105996131](genomebrowse://api/zoom?locus=14:20443678-105996131) | 6013 | 1 | Het Deletion | ABCD4,ABHD4,ABHD12B,ACIN1,ACOT1,ACOT2,ACOT4,ACOT6,ACTN1,ACTR10,ACYP1,ADAM20,ADAM21,ADCK1,ADCY4,ADSSL1,AHNAK2,AHSA1,AJUBA,AK7,AKAP5,AKAP6,AKT1,ALDH6A1,ALKBH1,AMN,ANG,ANGEL1,ANKRD9,AP1G2,AP4S1,AP5M1,APEX1,APOPT1,AREL1,ARF6,ARG2,ARHGAP5,ARHGEF40,ARID4A,ASB2,ASPG,ATG2B,ATG14,ATL1,ATP5S,ATP6V1D,ATXN3,BAG5,BATF,BAZ1A,BBOF1,BCL2L2,BCL2L2-PABPN1,BCL11B,BDKRB1,BDKRB2,BEGAIN,BMP4,BRF1,BRMS1L,BTBD6,BTBD7,C14orf1,C14orf2,C14orf28,C14orf37,C14orf39,C14orf79,C14orf80,C14orf93,C14orf105,C14orf119,C14orf132,C14orf159,C14orf166,C14orf177,C14orf178,C14orf180,CALM1,CARMIL3,CATSPERB,CBLN3,CCDC85C,CCDC88C,CCDC175,CCDC177,CCNB1IP1,CCNK,CDC42BPB,CDCA4,CDH24,CDKL1,CDKN3,CEBPE,CEP128,CEP170B,CFL2,CGRRF1,CHD8,CHGA,CHMP4A,CHURC1,CHURC1-FNTB,CIDEB,CINP,CIPC,CKB,CLEC14A,CLMN,CMA1,CMTM5,CNIH1,COCH,COQ6,COX8C,COX16,CPNE6,CPSF2,CRIP1,CRIP2,CTSG,CYP46A1,DAAM1,DACT1,DAD1,DCAF4,DCAF5,DCAF11,DDHD1,DDX24,DEGS2,DHRS1,DHRS2,DHRS4,DHRS4L1,DHRS4L2,DHRS7,DICER1,DIO2,DIO3,DLGAP5,DLK1,DLST,DNAAF2,DNAL1,DPF3,DTD2,DYNC1H1,EAPP,EDDM3A,EDDM3B,EFCAB11,EFS,EGLN3,EIF2B2,EIF2S1,EIF5,ELMSAN1,EMC9,EML1,EML5,ENTPD5,ERH,ERO1A,ESR2,ESRRB,EVL,EXD2,EXOC3L4,EXOC5,FAM71D,FAM161B,FAM177A1,FAM179B,FAM181A,FANCM,FBLN5,FBXO33,FBXO34,FCF1,FERMT2,FITM1,FKBP3,FLRT2,FLVCR2,FNTB,FOS,FOXA1,FOXG1,FOXN3,FRMD6,FSCB,FUT8,G2E3,GALC,GALNT16,GCH1,GEMIN2,GLRX5,GMFB,GMPR2,GNG2,GNPNAT1,GOLGA5,GON7,GPATCH2L,GPHB5,GPHN,GPR33,GPR65,GPR68,GPR132,GPR135,GPR137C,GPX2,GSC,GSKIP,GSTZ1,GTF2A1,GZMB,GZMH,HAUS4,HEATR4,HEATR5A,HECTD1,HHIPL1,HIF1A,HNRNPC,HOMEZ,HSP90AA1,HSPA2,IFI27,IFI27L1,IFI27L2,IFT43,IL25,INF2,INSM2,IPO4,IRF2BPL,IRF9,ISCA2,ISM2,ITPK1,JAG2,JDP2,JKAMP,JPH4,KCNH5,KCNK10,KCNK13,KHNYN,KIAA0391,KIAA0586,KIF26A,KLC1,KLHDC1,KLHDC2,KLHL28,KLHL33,KTN1,L2HGDH,L3HYPDH,LGALS3,LGMN,LIN52,LOC107984640,LRFN5,LRP10,LRR1,LRRC74A,LTB4R,LTB4R2,LTBP2,MAP3K9,MAP4K5,MAPK1IP1L,MARK3,MAX,MBIP,MDGA2,MDP1,MED6,METTL3,METTL17,MGAT2,MIA2,MIPOL1,MIS18BP1,MLH3,MMP14,MNAT1,MOAP1,MOK,MPP5,MRPL52,MTA1,MTHFD1,MYH6,MYH7,NAA30,NDRG2,NDUFB1,NEDD8,NEDD8-MDP1,NEK9,NEMF,NFATC4,NFKBIA,NGB,NGDN,NID2,NIN,NKX2-1,NKX2-8,NOP9,NOVA1,NOXRED1,NPAS3,NPC2,NRDE2,NRL,NRXN3,NUBPL,NUDT14,NUMB,NYNRIN,OR4E1,OR4E2,OR4K13,OR4K14,OR4K15,OR4K17,OR4L1,OR4N5,OR5AU1,OR6S1,OR10G2,OR10G3,OR11G2,OR11H4,OR11H6,OSGEP,OTUB2,OTX2,OXA1L,PABPN1,PACS2,PAPLN,PAPOLA,PARP2,PAX9,PCK2,PCNX1,PCNX4,PELI2,PGF,PIGH,PLD4,PLEK2,PLEKHD1,PLEKHG3,PLEKHH1,PNMA1,PNN,PNP,POLE2,POMT2,PPM1A,PPP1R3E,PPP1R13B,PPP1R36,PPP2R3C,PPP2R5C,PPP2R5E,PPP4R3A,PPP4R4,PRIMA1,PRKCH,PRKD1,PRMT5,PROX2,PRPF39,PSEN1,PSMA3,PSMA6,PSMB5,PSMB11,PSMC1,PSMC6,PSME1,PSME2,PTGDR,PTGER2,PTGR2,PTPN21,PYGL,RAB2B,RAB15,RABGGTA,RAD51B,RALGAPA1,RBM23,RBM25,RCOR1,RD3L,RDH11,RDH12,REC8,REM2,RGS6,RHOJ,RIN3,RIOX1,RIPK3,RNASE1,RNASE2,RNASE3,RNASE4,RNASE6,RNASE7,RNASE8,RNASE9,RNASE10,RNASE11,RNASE12,RNASE13,RNF31,RNF212B,RPGRIP1,RPL10L,RPL36AL,RPS6KA5,RPS6KL1,RPS29,RTL1,RTN1,SALL2,SAMD4A,SAMD15,SAV1,SCFD1,SDR39U1,SEC23A,SEL1L,SERPINA1,SERPINA3,SERPINA4,SERPINA5,SERPINA6,SERPINA9,SERPINA10,SERPINA11,SERPINA12,SETD3,SFTA3,SGPP1,SIPA1L1,SIVA1,SIX1,SIX4,SIX6,SLC7A7,SLC7A8,SLC8A3,SLC10A1,SLC22A17,SLC24A4,SLC25A21,SLC25A29,SLC25A47,SLC35F4,SLC38A6,SLC39A2,SLC39A9,SLIRP,SMOC1,SNAPC1,SNW1,SNX6,SOCS4,SOS2,SPATA7,SPTB,SPTLC2,SPTSSA,SRP54,SRSF5,SSTR1,STON2,STRN3,STXBP6,STYX,SUPT16H,SUSD6,SYNDIG1L,SYNE2,SYNE3,SYNJ2BP,SYNJ2BP-COX16,SYT16,TBPL2,TC2N,TCL1A,TCL1B,TDP1,TDRD9,TECPR2,TEP1,TEX22,TGFB3,TGM1,THTPA,TIMM9,TINF2,TM9SF1,TMED8,TMED10,TMEM30B,TMEM55B,TMEM63C,TMEM121,TMEM179,TMEM229B,TMEM251,TMEM253,TMEM260,TMX1,TNFAIP2,TOMM20L,TOX4,TPPP2,TRAF3,TRAPPC6B,TRIM9,TRIP11,TRMT5,TRMT61A,TSHR,TSSK4,TTC5,TTC6,TTC7B,TTC8,TTC9,TTLL5,TXNDC16,UBR7,UNC79,VASH1,VCPKMT,VIPAS39,VRK1,VRTN,VSX2,VTI1B,WARS,WDHD1,WDR20,WDR25,WDR89,XRCC3,YLPM1,YY1,ZBTB1,ZBTB25,ZBTB42,ZC2HC1C,ZC3H14,ZDHHC22,ZFHX2,ZFP36L1,ZFYVE1,ZFYVE21,ZFYVE26,ZNF219,ZNF410,ZNF839 | 579 |
| [15:43864967-43892880](genomebrowse://api/zoom?locus=15:43864967-43892880) | 34 | 2 | Duplicate | CKMT1B,PPIP5K1,STRC | 3 |
| [15:63667857-64040057](genomebrowse://api/zoom?locus=15:63667857-64040057) | 87 | 2 | Het Deletion | CA12,FBXL22,HERC1,USP3 | 4 |
| [16:14782022-15091683](genomebrowse://api/zoom?locus=16:14782022-15091683) | 60 | 3 | Duplicate | NOMO1,NPIPA1,NPIPA2,NPIPA3,PDXDC1,PLA2G10 | 6 |
| [19:16847445-16973370](genomebrowse://api/zoom?locus=19:16847445-16973370) | 27 | 1 | Het Deletion | NWD1,SIN3B | 2 |
| [19:16977184-17559271](genomebrowse://api/zoom?locus=19:16977184-17559271) | 209 | 1 | Duplicate | ABHD8,ANKLE1,ANO8,BABAM1,BST2,CPAMD8,DDA1,F2RL3,GTPBP3,HAUS8,MRPL34,MVB12A,MYO9B,NR2F6,OCEL1,PLVAP,SIN3B,TMEM221,USE1,USHBP1 | 20 |
| [Y:9175119-9368097](genomebrowse://api/zoom?locus=Y:9175119-9368097) | 30 | 3 | Duplicate | TSPY1,TSPY3,TSPY4,TSPY8,TSPY10 | 5 |

Lesion: C1

| **Region** | **# Targets** | **# Samples** | **CNV State** | **CNV State** | **Flags** |
| --- | --- | --- | --- | --- | --- |
| [1:12954417-13745175](genomebrowse://api/zoom?locus=1:12954417-13745175) | 40 | 1 | Duplicate | HNRNPCL2,PRAMEF5,PRAMEF6,PRAMEF7,PRAMEF8,PRAMEF10,PRAMEF13,PRAMEF14,PRAMEF15,PRAMEF17,PRAMEF18,PRAMEF19,PRAMEF20,PRAMEF25,PRAMEF33P | 15 |
| [1:145303910-145320619](genomebrowse://api/zoom?locus=1:145303910-145320619) | 17 | 1 | Duplicate | NBPF10 | 1 |
| [2:107042450-107075788](genomebrowse://api/zoom?locus=2:107042450-107075788) | 18 | 2 | Duplicate | RGPD3 | 1 |
| [2:131350431-131415450](genomebrowse://api/zoom?locus=2:131350431-131415450) | 21 | 3 | Duplicate | CFC1,POTEJ | 2 |
| [4:52709409-55161439](genomebrowse://api/zoom?locus=4:52709409-55161439) | 122 | 2 | Duplicate | CHIC2,DCUN1D4,ERVMER34-1,FIP1L1,GSX2,LNX1,LRRC66,PDGFRA,RASL11B,SCFD2,SGCB,SPATA18,USP46 | 13 |
| [6:31994229-31997115](genomebrowse://api/zoom?locus=6:31994229-31997115) | 11 | 4 | Duplicate | C4B | 1 |
| [8:11967409-12580763](genomebrowse://api/zoom?locus=8:11967409-12580763) | 24 | 1 | Duplicate | DEFB130,FAM86B1,FAM86B2,LONRF1,USP17L2,USP17L7,ZNF705D | 7 |
| [15:43904989-43922930](genomebrowse://api/zoom?locus=15:43904989-43922930) | 10 | 1 | Duplicate | CATSPER2,STRC | 2 |
| [16:14782022-15091683](genomebrowse://api/zoom?locus=16:14782022-15091683) | 60 | 3 | Duplicate | NOMO1,NPIPA1,NPIPA2,NPIPA3,PDXDC1,PLA2G10 | 6 |
| [16:18414874-18573362](genomebrowse://api/zoom?locus=16:18414874-18573362) | 37 | 3 | Duplicate | NOMO2,NPIPA8 | 2 |
| [16:29392767-29473271](genomebrowse://api/zoom?locus=16:29392767-29473271) | 23 | 4 | Duplicate | BOLA2,BOLA2-SMG1P6,NPIPB11,SLX1B,SULT1A4 | 5 |

Lesion D1

| **Region** | **# Targets** | **# Samples** | **CNV State** | **CNV State** | **Flags** |
| --- | --- | --- | --- | --- | --- |
| [1:145303910-145318226](genomebrowse://api/zoom?locus=1:145303910-145318226) | 14 | 1 | Duplicate | NBPF10 | 1 |
| [1:147410828-149291038](genomebrowse://api/zoom?locus=1:147410828-149291038) | 53 | 3 | Duplicate | GPR89B,LOC388692,NBPF11,NBPF15,PPIAL4D,PPIAL4E,PPIAL4F,PPIAL4G | 8 |
| [2:32631567-33246273](genomebrowse://api/zoom?locus=2:32631567-33246273) | 89 | 2 | Duplicate | BIRC6,LTBP1,TTC27 | 3 |
| [2:131350431-131415450](genomebrowse://api/zoom?locus=2:131350431-131415450) | 21 | 3 | Duplicate | CFC1,POTEJ | 2 |
| [4:52709409-55161439](genomebrowse://api/zoom?locus=4:52709409-55161439) | 122 | 2 | Duplicate | CHIC2,DCUN1D4,ERVMER34-1,FIP1L1,GSX2,LNX1,LRRC66,PDGFRA,RASL11B,SCFD2,SGCB,SPATA18,USP46 | 13 |
| [6:30569326-30572871](genomebrowse://api/zoom?locus=6:30569326-30572871) | 10 | 1 | Duplicate | PPP1R10 | 1 |
| [6:31994229-31997115](genomebrowse://api/zoom?locus=6:31994229-31997115) | 11 | 4 | Duplicate | C4B | 1 |
| [8:7286506-7740090](genomebrowse://api/zoom?locus=8:7286506-7740090) | 41 | 1 | Duplicate | DEFB103A,DEFB103B,DEFB104A,DEFB104B,DEFB105A,DEFB105B,DEFB106A,DEFB106B,DEFB107A,DEFB107B,PRR23D1,PRR23D2,SPAG11A,SPAG11B | 14 |
| [8:11921898-12293852](genomebrowse://api/zoom?locus=8:11921898-12293852) | 26 | 1 | Duplicate | DEFB130,FAM86B1,FAM86B2,LOC100133267,USP17L2,USP17L7,ZNF705D | 7 |
| [9:42386747-70919121](genomebrowse://api/zoom?locus=9:42386747-70919121) | 135 | 1 | Duplicate | ANKRD20A1,ANKRD20A2,ANKRD20A3,ANKRD20A4,CBWD3,CBWD5,CBWD6,CNTNAP3B,FOXD4L3,FOXD4L4,FOXD4L5,FOXD4L6,SPATA31A6,SPATA31A7 | 14 |
| [16:14813684-15091683](genomebrowse://api/zoom?locus=16:14813684-15091683) | 55 | 1 | Duplicate | NOMO1,NPIPA1,NPIPA2,NPIPA3,PDXDC1 | 5 |
| [16:18414874-18573362](genomebrowse://api/zoom?locus=16:18414874-18573362) | 37 | 3 | Duplicate | NOMO2,NPIPA8 | 2 |
| [16:29392767-29473271](genomebrowse://api/zoom?locus=16:29392767-29473271) | 23 | 4 | Duplicate | BOLA2,BOLA2-SMG1P6,NPIPB11,SLX1B,SULT1A4 | 5 |
| [X:49161883-49368396](genomebrowse://api/zoom?locus=X:49161883-49368396) | 62 | 1 | Duplicate | GAGE1,GAGE2A,GAGE2B,GAGE2C,GAGE2D,GAGE10,GAGE12B,GAGE12C,GAGE12D,GAGE12E,GAGE12F,GAGE12G,GAGE12H,GAGE12I,GAGE12J,GAGE13 | 16 |
| [Y:9175119-9368097](genomebrowse://api/zoom?locus=Y:9175119-9368097) | 30 | 3 | Duplicate | TSPY1,TSPY3,TSPY4,TSPY8,TSPY10 | 5 |

GBM-2

Blood

| **Region** | **# Targets** | **# Samples** | **CNV State** | **Gene Names** | **# Genes** |
| --- | --- | --- | --- | --- | --- |
| [1:12954417-13745175](genomebrowse://api/zoom?locus=1:12954417-13745175) | 40 | 4 | Duplicate | HNRNPCL2,PRAMEF5,PRAMEF6,PRAMEF7,PRAMEF8,PRAMEF10,PRAMEF13,PRAMEF14,PRAMEF15,PRAMEF17,PRAMEF18,PRAMEF19,PRAMEF20,PRAMEF25,PRAMEF33P | 15 |
| [1:145304447-145318226](genomebrowse://api/zoom?locus=1:145304447-145318226) | 13 | 1 | Duplicate | NBPF10 | 1 |
| [6:31948781-31998278](genomebrowse://api/zoom?locus=6:31948781-31998278) | 73 | 2 | Duplicate | C4A,C4B,STK19 | 3 |
| [6:32008646-32013103](genomebrowse://api/zoom?locus=6:32008646-32013103) | 14 | 2 | Duplicate | CYP21A2,TNXB | 2 |
| [8:7194637-7754132](genomebrowse://api/zoom?locus=8:7194637-7754132) | 51 | 2 | Duplicate | DEFB4A,DEFB4B,DEFB103A,DEFB103B,DEFB104A,DEFB104B,DEFB105A,DEFB105B,DEFB106A,DEFB106B,DEFB107A,DEFB107B,PRR23D1,PRR23D2,SPAG11A,SPAG11B,USP17L4,ZNF705G | 18 |
| [15:20739497-22835969](genomebrowse://api/zoom?locus=15:20739497-22835969) | 45 | 1 | Duplicate | GOLGA6L1,GOLGA6L6,OR4M2,OR4N4,POTEB,POTEB2,TUBGCP5 | 7 |
| [15:72948853-72958683](genomebrowse://api/zoom?locus=15:72948853-72958683) | 17 | 1 | Duplicate | GOLGA6B | 1 |
| [15:74363251-74373076](genomebrowse://api/zoom?locus=15:74363251-74373076) | 17 | 1 | Duplicate | GOLGA6A | 1 |
| [16:18414874-18573362](genomebrowse://api/zoom?locus=16:18414874-18573362) | 37 | 4 | Duplicate | NOMO2,NPIPA8 | 2 |
| [16:28659456-28834879](genomebrowse://api/zoom?locus=16:28659456-28834879) | 35 | 2 | Duplicate | ATXN2L,EIF3C,NPIPB8,NPIPB9 | 4 |

Lesion:

| **Region** | **# Targets** | **# Samples** | **CNV State** | **CNV State** | **Flags** |  |
| --- | --- | --- | --- | --- | --- | --- |
| [2:107051093-107075788](genomebrowse://api/zoom?locus=2:107051093-107075788) | 13 | 1 | Duplicate | RGPD3 | 1 |  |
| [2:112551810-112580177](genomebrowse://api/zoom?locus=2:112551810-112580177) | 12 | 2 | Duplicate | ANAPC1 | 1 |  |
| [2:113156800-113180236](genomebrowse://api/zoom?locus=2:113156800-113180236) | 13 | 1 | Duplicate | RGPD8 | 1 |  |
| [2:128322808-128391864](genomebrowse://api/zoom?locus=2:128322808-128391864) | 37 | 1 | Het Deletion | | MYO7B | 1 |
| [2:152437311-152450832](genomebrowse://api/zoom?locus=2:152437311-152450832) | 12 | 1 | Duplicate | NEB | 1 |  |
| [6:31936463-31940288](genomebrowse://api/zoom?locus=6:31936463-31940288) | 12 | 1 | Duplicate | DXO,SKIV2L,STK19 | 3 |  |
| [6:31965482-31994899](genomebrowse://api/zoom?locus=6:31965482-31994899) | 31 | 1 | Duplicate | C4A,C4B | 2 |  |
| [6:32009126-32013103](genomebrowse://api/zoom?locus=6:32009126-32013103) | 13 | 1 | Duplicate | CYP21A2,TNXB | 2 |  |
| [6:33166946-33171590](genomebrowse://api/zoom?locus=6:33166946-33171590) | 10 | 1 | Duplicate | RXRB,SLC39A7 | 2 |  |
| [6:33282789-33359774](genomebrowse://api/zoom?locus=6:33282789-33359774) | 10 | 1 | Duplicate | DAXX,KIFC1,ZBTB22 | 3 |  |
| [9:14088196-19577020](genomebrowse://api/zoom?locus=9:14088196-19577020) | 287 | 1 | Duplicate | ACER2,ADAMTSL1,BNC2,C9orf92,CCDC171,CER1,CNTLN,DENND4C,FREM1,HAUS6,NFIB,PLIN2,PSIP1,RPS6,RRAGA,SAXO1,SH3GL2,SLC24A2,SNAPC3,TTC39B,ZDHHC21 | 21 |  |
| [12:133501959-133698504](genomebrowse://api/zoom?locus=12:133501959-133698504) | 18 | 1 | Duplicate | ZNF26,ZNF84,ZNF140,ZNF605,ZNF891 | 5 |  |
| [14:32902700-34247778](genomebrowse://api/zoom?locus=14:32902700-34247778) | 22 | 1 | Het Deletion | | AKAP6,NPAS3 | 2 |
| [15:32885755-32908541](genomebrowse://api/zoom?locus=15:32885755-32908541) | 20 | 1 | Duplicate | ARHGAP11A,GOLGA8N | 2 |  |
| [15:82824389-83213452](genomebrowse://api/zoom?locus=15:82824389-83213452) | 13 | 2 | Duplicate | CPEB1,GOLGA6L10,RPS17 | 3 |  |
| [16:28659456-28784076](genomebrowse://api/zoom?locus=16:28659456-28784076) | 34 | 2 | Duplicate | EIF3C,NPIPB8,NPIPB9 | 3 |  |
| [16:29883508-29910340](genomebrowse://api/zoom?locus=16:29883508-29910340) | 17 | 1 | Het Deletion | | SEZ6L2 | 1 |
| [16:70852245-70883850](genomebrowse://api/zoom?locus=16:70852245-70883850) | 10 | 1 | Duplicate | HYDIN | 1 |  |
| [19:2037738-2046690](genomebrowse://api/zoom?locus=19:2037738-2046690) | 13 | 1 | Duplicate | MKNK2 | 1 |  |
| [19:2986981-3005966](genomebrowse://api/zoom?locus=19:2986981-3005966) | 16 | 1 | Duplicate | TLE2,TLE6 | 2 |  |
| [19:3747792-3778420](genomebrowse://api/zoom?locus=19:3747792-3778420) | 19 | 1 | Duplicate | APBA3,MATK,MRPL54,RAX2,TJP3 | 5 |  |
| [19:4325213-4343597](genomebrowse://api/zoom?locus=19:4325213-4343597) | 12 | 1 | Duplicate | MPND,STAP2 | 2 |  |
| [19:51010831-51126012](genomebrowse://api/zoom?locus=19:51010831-51126012) | 13 | 2 | Duplicate | ASPDH,JOSD2,LRRC4B,SYT3 | 4 |  |
| [19:51883759-51955887](genomebrowse://api/zoom?locus=19:51883759-51955887) | 17 | 1 | Duplicate | C19orf84,LIM2,LOC100129083,SIGLEC8,SIGLEC10 | 5 |  |
| [19:54684490-54743827](genomebrowse://api/zoom?locus=19:54684490-54743827) | 23 | 1 | Duplicate | LILRB3,MBOAT7,RPS9,TSEN34 | 4 |  |
| [19:54756229-54781793](genomebrowse://api/zoom?locus=19:54756229-54781793) | 18 | 1 | Duplicate | LILRB2,LILRB5 | 2 |  |
| [19:55597209-55607703](genomebrowse://api/zoom?locus=19:55597209-55607703) | 22 | 1 | Duplicate | EPS8L1,PPP1R12C | 2 |  |
| [19:55869513-55888394](genomebrowse://api/zoom?locus=19:55869513-55888394) | 16 | 1 | Duplicate | FAM71E2,IL11,TMEM190 | 3 |  |

Lesion:

| **Region** | **# Targets** | **# Samples** | **CNV State** | **CNV State** | **Flags** |  |
| --- | --- | --- | --- | --- | --- | --- |
| [1:9770514-9782414](genomebrowse://api/zoom?locus=1:9770514-9782414) | 16 | 1 | Het Deletion | | PIK3CD | 1 |
| [1:145304447-145320619](genomebrowse://api/zoom?locus=1:145304447-145320619) | 16 | 1 | Duplicate | NBPF10 | 1 |  |
| [1:145748338-145816740](genomebrowse://api/zoom?locus=1:145748338-145816740) | 19 | 2 | Duplicate | GPR89A,PDZK1 | 2 |  |
| [2:113135630-113180236](genomebrowse://api/zoom?locus=2:113135630-113180236) | 20 | 1 | Duplicate | RGPD8 | 1 |  |
| [2:131350431-131415450](genomebrowse://api/zoom?locus=2:131350431-131415450) | 21 | 2 | Duplicate | CFC1,POTEJ | 2 |  |
| [6:31964921-31994899](genomebrowse://api/zoom?locus=6:31964921-31994899) | 32 | 1 | Duplicate | C4A,C4B | 2 |  |
| [7:2579178-2587112](genomebrowse://api/zoom?locus=7:2579178-2587112) | 10 | 1 | Duplicate | BRAT1 | 1 |  |
| [8:11921898-12047397](genomebrowse://api/zoom?locus=8:11921898-12047397) | 14 | 1 | Duplicate | FAM86B1,LOC100133267,USP17L2,USP17L7,ZNF705D | 5 |  |
| [11:60997391-61026784](genomebrowse://api/zoom?locus=11:60997391-61026784) | 13 | 1 | Duplicate | PGA4,PGA5,VWCE | 3 |  |
| [12:6687575-6702394](genomebrowse://api/zoom?locus=12:6687575-6702394) | 19 | 1 | Duplicate | CHD4 | 1 |  |
| [15:30697833-30706365](genomebrowse://api/zoom?locus=15:30697833-30706365) | 14 | 1 | Duplicate | GOLGA8R | 1 |  |
| [15:75576991-75586602](genomebrowse://api/zoom?locus=15:75576991-75586602) | 16 | 1 | Duplicate | GOLGA6D | 1 |  |
| [16:28659456-28784076](genomebrowse://api/zoom?locus=16:28659456-28784076) | 34 | 2 | Duplicate | EIF3C,NPIPB8,NPIPB9 | 3 |  |
| [19:43858005-43965999](genomebrowse://api/zoom?locus=19:43858005-43965999) | 15 | 1 | Duplicate | CD177,LYPD3,TEX101 | 3 |  |
| [19:43979562-44001431](genomebrowse://api/zoom?locus=19:43979562-44001431) | 11 | 1 | Duplicate | PHLDB3 | 1 |  |
| [19:44039181-44058956](genomebrowse://api/zoom?locus=19:44039181-44058956) | 15 | 1 | Duplicate | XRCC1,ZNF575 | 2 |  |
| [19:44237010-44252204](genomebrowse://api/zoom?locus=19:44237010-44252204) | 11 | 1 | Duplicate | SMG9 | 1 |  |
| [19:49136701-49147826](genomebrowse://api/zoom?locus=19:49136701-49147826) | 10 | 1 | Duplicate | CA11,DBP | 2 |  |
| [19:49173613-49220000](genomebrowse://api/zoom?locus=19:49173613-49220000) | 10 | 1 | Duplicate | FUT2,MAMSTR,NTN5 | 3 |  |
| [19:49714751-49840290](genomebrowse://api/zoom?locus=19:49714751-49840290) | 15 | 1 | Duplicate | CD37,SLC6A16,TRPM4 | 3 |  |
| [19:51010831-51126012](genomebrowse://api/zoom?locus=19:51010831-51126012) | 13 | 2 | Duplicate | ASPDH,JOSD2,LRRC4B,SYT3 | 4 |  |
| [19:51893732-51920719](genomebrowse://api/zoom?locus=19:51893732-51920719) | 11 | 1 | Duplicate | C19orf84,LOC100129083,SIGLEC10 | 3 |  |
| [19:54757881-54781793](genomebrowse://api/zoom?locus=19:54757881-54781793) | 15 | 1 | Duplicate | LILRB2,LILRB5 | 2 |  |
| [19:55603990-55607703](genomebrowse://api/zoom?locus=19:55603990-55607703) | 11 | 1 | Duplicate | PPP1R12C | 1 |  |
| [19:59063016-59068524](genomebrowse://api/zoom?locus=19:59063016-59068524) | 10 | 1 | Duplicate | CHMP2A,UBE2M | 2 |  |
| [Y:9175119-9367192](genomebrowse://api/zoom?locus=Y:9175119-9367192) | 28 | 1 | Duplicate | TSPY1,TSPY3,TSPY4,TSPY8,TSPY10 | 5 |  |

Lesion:

| **Region** | **# Targets** | **# Samples** | **CNV State** | **CNV State** | **Flags** |
| --- | --- | --- | --- | --- | --- |
| [2:152437997-152450832](genomebrowse://api/zoom?locus=2:152437997-152450832) | 11 | 1 | Duplicate | NEB | 1 |
| [7:142423216-142494975](genomebrowse://api/zoom?locus=7:142423216-142494975) | 16 | 1 | Duplicate | PRSS1 | 1 |
| [8:7309781-7754132](genomebrowse://api/zoom?locus=8:7309781-7754132) | 38 | 1 | Duplicate | DEFB4A,DEFB103A,DEFB104A,DEFB104B,DEFB105A,DEFB105B,DEFB106A,DEFB106B,DEFB107A,DEFB107B,PRR23D1,PRR23D2,SPAG11A,SPAG11B | 14 |
| [X:134873998-134967496](genomebrowse://api/zoom?locus=X:134873998-134967496) | 17 | 2 | Duplicate | CT45A3,CT45A5,CT45A8,CT45A9,CT45A10 | 5 |

Lesion:

| **Region** | **# Targets** | **# Samples** | **CNV State** | **Gene Names** | **# Genes** |
| --- | --- | --- | --- | --- | --- |
| [1:145304447-145325343](genomebrowse://api/zoom?locus=1:145304447-145325343) | 22 | 1 | Duplicate | NBPF10 | 1 |
| [1:145748338-145818826](genomebrowse://api/zoom?locus=1:145748338-145818826) | 20 | 1 | Duplicate | GPR89A,PDZK1 | 2 |
| [1:147410828-148806584](genomebrowse://api/zoom?locus=1:147410828-148806584) | 52 | 4 | Duplicate | GPR89B,NBPF11,NBPF15,PPIAL4D,PPIAL4E,PPIAL4F,PPIAL4G | 7 |
| [1:152636582-153177480](genomebrowse://api/zoom?locus=1:152636582-153177480) | 27 | 1 | Duplicate | C1orf68,IVL,KPRP,LCE1A,LCE1B,LCE1C,LCE1D,LCE1E,LCE1F,LCE2A,LCE2B,LCE2C,LCE2D,LCE4A,LCE6A,LELP1,SMCP,SPRR1A,SPRR1B,SPRR2A,SPRR2B,SPRR2D,SPRR2E,SPRR2F,SPRR2G,SPRR3,SPRR4 | 27 |
| [1:155580040-155631214](genomebrowse://api/zoom?locus=1:155580040-155631214) | 16 | 4 | Duplicate | MSTO1,YY1AP1 | 2 |
| [1:161483685-161643863](genomebrowse://api/zoom?locus=1:161483685-161643863) | 18 | 2 | Duplicate | FCGR2A,FCGR2B,FCGR3A,FCGR3B,HSPA6 | 5 |
| [2:97875418-97914951](genomebrowse://api/zoom?locus=2:97875418-97914951) | 19 | 4 | Duplicate | ANKRD36 | 1 |
| [2:112526868-112580177](genomebrowse://api/zoom?locus=2:112526868-112580177) | 24 | 5 | Duplicate | ANAPC1 | 1 |
| [2:131985817-132022256](genomebrowse://api/zoom?locus=2:131985817-132022256) | 11 | 1 | Duplicate | POTEE | 1 |
| [2:152437997-152450832](genomebrowse://api/zoom?locus=2:152437997-152450832) | 11 | 1 | Duplicate | NEB | 1 |
| [7:101960798-102016769](genomebrowse://api/zoom?locus=7:101960798-102016769) | 12 | 1 | Duplicate | LOC100289561,SH2B2,SPDYE6 | 3 |
| [9:41503037-70919121](genomebrowse://api/zoom?locus=9:41503037-70919121) | 142 | 1 | Duplicate | ANKRD20A1,ANKRD20A2,ANKRD20A3,ANKRD20A4,CBWD3,CBWD5,CBWD6,CNTNAP3B,FOXD4L3,FOXD4L4,FOXD4L5,FOXD4L6,SPATA31A5,SPATA31A6,SPATA31A7 | 15 |
| [10:48739388-49389051](genomebrowse://api/zoom?locus=10:48739388-49389051) | 22 | 1 | Duplicate | FAM25C,FRMPD2,PTPN20 | 3 |
| [15:30375256-30385702](genomebrowse://api/zoom?locus=15:30375256-30385702) | 19 | 1 | Duplicate | GOLGA8J | 1 |
| [15:30696204-30900271](genomebrowse://api/zoom?locus=15:30696204-30900271) | 23 | 1 | Duplicate | GOLGA8H,GOLGA8R | 2 |
| [15:32885755-32912379](genomebrowse://api/zoom?locus=15:32885755-32912379) | 21 | 1 | Duplicate | ARHGAP11A,GOLGA8N | 2 |
| [15:34654397-34825163](genomebrowse://api/zoom?locus=15:34654397-34825163) | 40 | 4 | Duplicate | GOLGA8A,GOLGA8B,LPCAT4 | 3 |
| [15:43864967-43892880](genomebrowse://api/zoom?locus=15:43864967-43892880) | 34 | 1 | Duplicate | CKMT1B,PPIP5K1,STRC | 3 |
| [15:72948853-72958683](genomebrowse://api/zoom?locus=15:72948853-72958683) | 17 | 2 | Duplicate | GOLGA6B | 1 |
| [15:74366834-74373076](genomebrowse://api/zoom?locus=15:74366834-74373076) | 10 | 2 | Duplicate | GOLGA6A | 1 |
| [16:14852804-15044112](genomebrowse://api/zoom?locus=16:14852804-15044112) | 44 | 5 | Duplicate | NOMO1,NPIPA1,NPIPA2 | 3 |
| [16:16313411-16485953](genomebrowse://api/zoom?locus=16:16313411-16485953) | 42 | 2 | Duplicate | ABCC6,NOMO3,NPIPA7 | 3 |
| [16:18414874-18794424](genomebrowse://api/zoom?locus=16:18414874-18794424) | 38 | 1 | Duplicate | NOMO2,NPIPA8,RPS15A | 3 |
| [16:28663278-28834879](genomebrowse://api/zoom?locus=16:28663278-28834879) | 34 | 1 | Duplicate | ATXN2L,EIF3C,NPIPB8,NPIPB9 | 4 |
| [16:70852245-70889178](genomebrowse://api/zoom?locus=16:70852245-70889178) | 12 | 2 | Duplicate | HYDIN | 1 |
| [16:70995862-71209644](genomebrowse://api/zoom?locus=16:70995862-71209644) | 34 | 1 | Duplicate | HYDIN | 1 |
| [X:49161883-49368396](genomebrowse://api/zoom?locus=X:49161883-49368396) | 62 | 1 | Duplicate | GAGE1,GAGE2A,GAGE2B,GAGE2C,GAGE2D,GAGE10,GAGE12B,GAGE12C,GAGE12D,GAGE12E,GAGE12F,GAGE12G,GAGE12H,GAGE12I,GAGE12J,GAGE13 | 16 |
| [Y:9175119-9368097](genomebrowse://api/zoom?locus=Y:9175119-9368097) | 30 | 5 | Duplicate | TSPY1,TSPY3,TSPY4,TSPY8,TSPY10 | 5 |

GBM:3

Blood

| **Region** | **# Targets** | **# Samples** | **CNV State** | **CNV State** | **Flags** |
| --- | --- | --- | --- | --- | --- |
| [1:12977513-13646345](genomebrowse://api/zoom?locus=1:12977513-13646345) | 26 | 2 | Duplicate | HNRNPCL2,PRAMEF5,PRAMEF6,PRAMEF7,PRAMEF8,PRAMEF13,PRAMEF15,PRAMEF18,PRAMEF25,PRAMEF33P | 10 |
| [1:145748338-145816740](genomebrowse://api/zoom?locus=1:145748338-145816740) | 19 | 3 | Duplicate | GPR89A,PDZK1 | 2 |
| [2:87069430-88125248](genomebrowse://api/zoom?locus=2:87069430-88125248) | 58 | 2 | Duplicate | CD8B,PLGLB1,PLGLB2,RGPD1,RGPD2 | 5 |
| [5:68809775-70297970](genomebrowse://api/zoom?locus=5:68809775-70297970) | 58 | 1 | Duplicate | GTF2H2C,NAIP,OCLN,SERF1A,SERF1B,SMN1,SMN2 | 7 |
| [6:32009789-32012493](genomebrowse://api/zoom?locus=6:32009789-32012493) | 10 | 2 | Duplicate | TNXB | 1 |
| [6:33143793-33146129](genomebrowse://api/zoom?locus=6:33143793-33146129) | 10 | 1 | Duplicate | COL11A2 | 1 |
| [7:102125486-102312048](genomebrowse://api/zoom?locus=7:102125486-102312048) | 67 | 1 | Duplicate | POLR2J2,POLR2J3,RASA4,RASA4B,SPDYE2,SPDYE2B,UPK3BL | 7 |
| [10:48739388-49392725](genomebrowse://api/zoom?locus=10:48739388-49392725) | 23 | 1 | Duplicate | FAM25C,FRMPD2,PTPN20 | 3 |
| [12:57919131-57926098](genomebrowse://api/zoom?locus=12:57919131-57926098) | 11 | 1 | Duplicate | DCTN2,MBD6 | 2 |
| [15:30375256-30706365](genomebrowse://api/zoom?locus=15:30375256-30706365) | 46 | 2 | Duplicate | CHRFAM7A,GOLGA8J,GOLGA8R | 3 |
| [15:43870268-43892880](genomebrowse://api/zoom?locus=15:43870268-43892880) | 23 | 2 | Duplicate | CKMT1B,PPIP5K1,STRC | 3 |
| [15:75561138-75586816](genomebrowse://api/zoom?locus=15:75561138-75586816) | 23 | 2 | Duplicate | GOLGA6C,GOLGA6D | 2 |
| [16:18414874-18573362](genomebrowse://api/zoom?locus=16:18414874-18573362) | 37 | 2 | Duplicate | NOMO2,NPIPA8 | 2 |
| [16:28659456-28834879](genomebrowse://api/zoom?locus=16:28659456-28834879) | 35 | 2 | Duplicate | ATXN2L,EIF3C,NPIPB8,NPIPB9 | 4 |
| [16:30205406-30365121](genomebrowse://api/zoom?locus=16:30205406-30365121) | 23 | 1 | Duplicate | BOLA2B,CD2BP2,NPIPB13,SLX1A,SULT1A3 | 5 |
| [X:49180222-49361336](genomebrowse://api/zoom?locus=X:49180222-49361336) | 55 | 4 | Duplicate | GAGE2A,GAGE2B,GAGE2C,GAGE2D,GAGE12B,GAGE12C,GAGE12D,GAGE12E,GAGE12F,GAGE12G,GAGE12H,GAGE12I,GAGE12J,GAGE13 | 14 |

Lesion

| **Region** | **# Targets** | **# Samples** | **CNV State** | **CNV State** | **Flags** |
| --- | --- | --- | --- | --- | --- |
| [2:113127755-113191030](genomebrowse://api/zoom?locus=2:113127755-113191030) | 23 | 1 | Duplicate | RGPD8 | 1 |
| [6:32009789-32012493](genomebrowse://api/zoom?locus=6:32009789-32012493) | 10 | 2 | Duplicate | TNXB | 1 |
| [15:30375256-30706365](genomebrowse://api/zoom?locus=15:30375256-30706365) | 46 | 2 | Duplicate | CHRFAM7A,GOLGA8J,GOLGA8R | 3 |
| [15:32885755-32896179](genomebrowse://api/zoom?locus=15:32885755-32896179) | 19 | 2 | Duplicate | GOLGA8N | 1 |
| [15:74363251-74373076](genomebrowse://api/zoom?locus=15:74363251-74373076) | 17 | 2 | Duplicate | GOLGA6A | 1 |
| [15:75561138-75586816](genomebrowse://api/zoom?locus=15:75561138-75586816) | 23 | 2 | Duplicate | GOLGA6C,GOLGA6D | 2 |
| [16:18414874-18796145](genomebrowse://api/zoom?locus=16:18414874-18796145) | 39 | 1 | Duplicate | NOMO2,NPIPA8,RPS15A | 3 |

Lesion:

| **Region** | **# Targets** | **# Samples** | **CNV State** | **CNV State** | **Flags** |  |
| --- | --- | --- | --- | --- | --- | --- |
| [2:237074557-238728994](genomebrowse://api/zoom?locus=2:237074557-238728994) | 133 | 1 | Het Deletion | | ACKR3,ASB18,COL6A3,COPS8,GBX2,IQCA1,LRRFIP1,MLPH,PRLH,RAB17,RBM44 | 11 |
| [3:37695205-38071030](genomebrowse://api/zoom?locus=3:37695205-38071030) | 55 | 1 | Het Deletion | | CTDSPL,ITGA9,PLCD1,VILL | 4 |
| [3:50645038-51198133](genomebrowse://api/zoom?locus=3:50645038-51198133) | 26 | 1 | Het Deletion | | CISH,DOCK3,MAPKAPK3 | 3 |
| [3:52939152-53003144](genomebrowse://api/zoom?locus=3:52939152-53003144) | 20 | 1 | Het Deletion | | SFMBT1 | 1 |
| [3:53684806-53894255](genomebrowse://api/zoom?locus=3:53684806-53894255) | 64 | 1 | Het Deletion | | CACNA1D,CHDH,IL17RB | 3 |
| [3:65342053-66502057](genomebrowse://api/zoom?locus=3:65342053-66502057) | 52 | 1 | Het Deletion | | LRIG1,MAGI1,SLC25A26 | 3 |
| [3:102153959-102196462](genomebrowse://api/zoom?locus=3:102153959-102196462) | 11 | 1 | Het Deletion | | ZPLD1 | 1 |
| [3:123813685-124281936](genomebrowse://api/zoom?locus=3:123813685-124281936) | 35 | 1 | Het Deletion | | KALRN | 1 |
| [3:185990044-186395672](genomebrowse://api/zoom?locus=3:185990044-186395672) | 51 | 1 | Het Deletion | | AHSG,CRYGS,DGKG,DNAJB11,FETUB,HRG,TBCCD1 | 7 |
| [3:189507590-190930423](genomebrowse://api/zoom?locus=3:189507590-190930423) | 59 | 1 | Het Deletion | | CLDN1,CLDN16,GMNC,IL1RAP,OSTN,P3H2,TMEM207,TP63 | 8 |
| [15:32885755-32896179](genomebrowse://api/zoom?locus=15:32885755-32896179) | 19 | 2 | Duplicate | GOLGA8N | 1 |  |
| [15:74363465-74371030](genomebrowse://api/zoom?locus=15:74363465-74371030) | 15 | 1 | Duplicate | GOLGA6A | 1 |  |
| [16:28659456-28784076](genomebrowse://api/zoom?locus=16:28659456-28784076) | 34 | 2 | Duplicate | EIF3C,NPIPB8,NPIPB9 | 3 |  |
| [17:7452471-8079193](genomebrowse://api/zoom?locus=17:7452471-8079193) | 410 | 1 | Het Deletion | | ALOX12B,ALOX15B,ALOXE3,ATP1B2,CD68,CHD3,CNTROB,CYB5D1,DNAH2,EFNB3,EIF4A1,FXR2,GUCY2D,HES7,KCNAB3,KDM6B,LOC100996842,MPDU1,NAA38,PER1,SAT2,SENP3,SHBG,SOX15,TMEM88,TMEM107,TNFSF12,TNFSF12-TNFSF13,TNFSF13,TP53,TRAPPC1,VAMP2,WRAP53 | 33 |

| **Region** | **# Targets** | **# Samples** | **CNV State** | **CNV State** | **Flags** |
| --- | --- | --- | --- | --- | --- |
| [1:12977513-13717379](genomebrowse://api/zoom?locus=1:12977513-13717379) | 35 | 1 | Duplicate | HNRNPCL2,PRAMEF5,PRAMEF6,PRAMEF7,PRAMEF8,PRAMEF13,PRAMEF14,PRAMEF15,PRAMEF17,PRAMEF18,PRAMEF19,PRAMEF25,PRAMEF33P | 13 |
| [1:145304447-145315899](genomebrowse://api/zoom?locus=1:145304447-145315899) | 10 | 1 | Duplicate | NBPF10 | 1 |
| [1:145748338-145816740](genomebrowse://api/zoom?locus=1:145748338-145816740) | 19 | 3 | Duplicate | GPR89A,PDZK1 | 2 |
| [1:147400650-148806584](genomebrowse://api/zoom?locus=1:147400650-148806584) | 54 | 1 | Duplicate | GPR89B,NBPF11,NBPF15,PPIAL4D,PPIAL4E,PPIAL4F,PPIAL4G | 7 |
| [1:155181918-155209868](genomebrowse://api/zoom?locus=1:155181918-155209868) | 14 | 1 | Duplicate | GBA,MTX1 | 2 |
| [2:152437997-152459205](genomebrowse://api/zoom?locus=2:152437997-152459205) | 17 | 1 | Duplicate | NEB | 1 |
| [9:42406567-70919121](genomebrowse://api/zoom?locus=9:42406567-70919121) | 129 | 2 | Duplicate | ANKRD20A1,ANKRD20A2,ANKRD20A3,ANKRD20A4,CBWD3,CBWD5,CBWD6,CNTNAP3B,FOXD4L3,FOXD4L4,FOXD4L5,FOXD4L6,SPATA31A6,SPATA31A7 | 14 |
| [10:48754798-49389051](genomebrowse://api/zoom?locus=10:48754798-49389051) | 20 | 1 | Duplicate | FAM25C,FRMPD2,PTPN20 | 3 |
| [15:30375256-30704651](genomebrowse://api/zoom?locus=15:30375256-30704651) | 45 | 1 | Duplicate | CHRFAM7A,GOLGA8J,GOLGA8R | 3 |
| [15:43870268-43892880](genomebrowse://api/zoom?locus=15:43870268-43892880) | 23 | 2 | Duplicate | CKMT1B,PPIP5K1,STRC | 3 |
| [15:43902510-43922930](genomebrowse://api/zoom?locus=15:43902510-43922930) | 16 | 1 | Duplicate | CATSPER2,STRC | 2 |
| [15:74363251-74373076](genomebrowse://api/zoom?locus=15:74363251-74373076) | 17 | 2 | Duplicate | GOLGA6A | 1 |
| [16:18414874-18573362](genomebrowse://api/zoom?locus=16:18414874-18573362) | 37 | 2 | Duplicate | NOMO2,NPIPA8 | 2 |
| [16:28659456-28784076](genomebrowse://api/zoom?locus=16:28659456-28784076) | 34 | 2 | Duplicate | EIF3C,NPIPB8,NPIPB9 | 3 |

GBM-4

Blood

| **Region** | **# Targets** | **# Samples** | **CNV State** | **Gene Names** | **# Genes** |
| --- | --- | --- | --- | --- | --- |
| [1:12939365-13745175](genomebrowse://api/zoom?locus=1:12939365-13745175) | 44 | 1 | Duplicate | HNRNPCL2,PRAMEF4,PRAMEF5,PRAMEF6,PRAMEF7,PRAMEF8,PRAMEF10,PRAMEF13,PRAMEF14,PRAMEF15,PRAMEF17,PRAMEF18,PRAMEF19,PRAMEF20,PRAMEF25,PRAMEF33P | 16 |
| [1:104114225-104205633](genomebrowse://api/zoom?locus=1:104114225-104205633) | 29 | 1 | Duplicate | AMY1A,AMY2A,AMY2B | 3 |
| [1:147408741-149291038](genomebrowse://api/zoom?locus=1:147408741-149291038) | 54 | 1 | Duplicate | GPR89B,LOC388692,NBPF11,NBPF15,PPIAL4D,PPIAL4E,PPIAL4F,PPIAL4G | 8 |
| [1:155580040-155631214](genomebrowse://api/zoom?locus=1:155580040-155631214) | 16 | 4 | Duplicate | MSTO1,YY1AP1 | 2 |
| [2:242650792-242738569](genomebrowse://api/zoom?locus=2:242650792-242738569) | 19 | 1 | Duplicate | D2HGDH,GAL3ST2,ING5 | 3 |
| [5:70248266-70358590](genomebrowse://api/zoom?locus=5:70248266-70358590) | 32 | 1 | Duplicate | GTF2H2,NAIP,SMN1 | 3 |
| [7:143268911-143560889](genomebrowse://api/zoom?locus=7:143268911-143560889) | 18 | 2 | Duplicate | CTAGE6,CTAGE15,TCAF1,TCAF2 | 4 |
| [11:89665554-89820299](genomebrowse://api/zoom?locus=11:89665554-89820299) | 14 | 5 | Duplicate | TRIM49C,TRIM49D2,TRIM64,UBTFL1 | 4 |
| [15:43864967-43892880](genomebrowse://api/zoom?locus=15:43864967-43892880) | 34 | 1 | Duplicate | CKMT1B,PPIP5K1,STRC | 3 |
| [15:74363251-74373076](genomebrowse://api/zoom?locus=15:74363251-74373076) | 17 | 4 | Duplicate | GOLGA6A | 1 |
| [16:14782022-15044112](genomebrowse://api/zoom?locus=16:14782022-15044112) | 57 | 3 | Duplicate | NOMO1,NPIPA1,NPIPA2,NPIPA3,PLA2G10 | 5 |
| [16:18511694-18569113](genomebrowse://api/zoom?locus=16:18511694-18569113) | 30 | 3 | Duplicate | NOMO2 | 1 |
| [16:28663278-28784076](genomebrowse://api/zoom?locus=16:28663278-28784076) | 33 | 1 | Duplicate | EIF3C,NPIPB8,NPIPB9 | 3 |
| [22:16258186-17265299](genomebrowse://api/zoom?locus=22:16258186-17265299) | 13 | 3 | Duplicate | CCT8L2,OR11H1,POTEH,XKR3 | 4 |
| [X:134873998-134967496](genomebrowse://api/zoom?locus=X:134873998-134967496) | 17 | 3 | Duplicate | CT45A3,CT45A5,CT45A8,CT45A9,CT45A10 | 5 |

Lesion:

| **Region** | **# Targets** | **# Samples** | **CNV State** | **CNV State** | **Flags** |  |
| --- | --- | --- | --- | --- | --- | --- |
| [1:13000814-13646345](genomebrowse://api/zoom?locus=1:13000814-13646345) | 22 | 2 | Duplicate | HNRNPCL2,PRAMEF5,PRAMEF6,PRAMEF8,PRAMEF13,PRAMEF15,PRAMEF18,PRAMEF25,PRAMEF33P | 9 |  |
| [1:155182176-155209868](genomebrowse://api/zoom?locus=1:155182176-155209868) | 13 | 1 | Duplicate | GBA,MTX1 | 2 |  |
| [2:41608-51255411](genomebrowse://api/zoom?locus=2:41608-51255411) | 3095 | 1 | Het Deletion | | ABCG5,ABCG8,ABHD1,ACP1,ADAM17,ADCY3,ADGRF3,ADI1,AGBL5,ALK,ALLC,APOB,ARHGEF33,ASAP2,ASXL2,ATAD2B,ATL2,ATP6V1C2,ATP6V1E2,ATRAID,BIRC6,BRE,C1GALT1C1L,C2orf16,C2orf48,C2orf50,C2orf61,C2orf70,C2orf71,C2orf91,CAD,CALM2,CAMKMT,CAPN13,CAPN14,CCDC121,CDC42EP3,CDKL4,CEBPZ,CEBPZOS,CENPA,CENPO,CGREF1,CIB4,CLIP4,CMPK2,COLEC11,COX7A2L,CPSF3,CRIM1,CRIPT,CYP1B1,CYS1,DCDC2C,DDX1,DHX57,DNAJC5G,DNAJC27,DNMT3A,DPY30,DPYSL5,DRC1,DTNB,DYNC2LI1,E2F6,EFR3B,EHD3,EIF2AK2,EIF2B4,EMILIN1,EML4,EPAS1,EPCAM,FAM49A,FAM84A,FAM98A,FAM110C,FAM150B,FAM179A,FAM228A,FAM228B,FBXO11,FEZ2,FKBP1B,FNDC4,FOSL2,FOXN2,FSHR,GALM,GALNT14,GAREM2,GCKR,GDF7,GEMIN6,GEN1,GPATCH11,GPN1,GREB1,GRHL1,GTF2A1L,GTF3C2,HAAO,HADHA,HADHB,HEATR5B,HNRNPLL,HPCAL1,HS1BP3,IAH1,ID2,IFT172,ITGB1BP1,ITSN2,KCNF1,KCNG3,KCNK3,KCNK12,KCNS3,KHK,KIDINS220,KIF3C,KLF11,KLHL29,KRTCAP3,LAPTM4A,LBH,LCLAT1,LDAH,LHCGR,LPIN1,LRPPRC,LTBP1,MAP4K3,MAPRE3,MATN3,MBOAT2,MCFD2,MEMO1,MFSD2B,MORN2,MPV17,MRPL33,MSGN1,MSH2,MSH6,MTA3,MYCN,MYCNOS,MYT1L,NBAS,NCOA1,NDUFAF7,NLRC4,NOL10,NRBP1,NRXN1,NT5C1B,NT5C1B-RDH14,NTSR2,ODC1,OSR1,OST4,OTOF,OXER1,PDIA6,PFN4,PIGF,PKDCC,PLB1,PLEKHH2,POMC,PPM1B,PPM1G,PPP1CB,PPP1R21,PQLC3,PREB,PREPL,PRKCE,PRKD3,PRR30,PTRHD1,PUM2,PXDN,QPCT,RAB10,RAD51AP2,RASGRP3,RBKS,RDH14,RHOB,RHOQ,RMDN2,RNASEH1,RNF144A,ROCK2,RPS7,RRM2,RSAD2,SDC1,SELENOI,SF3B6,SH3YL1,SIX2,SIX3,SLC3A1,SLC4A1AP,SLC5A6,SLC8A1,SLC30A3,SLC30A6,SLC35F6,SMC6,SNTG2,SNX17,SOCS5,SOS1,SOX11,SPAST,SPDYA,SRBD1,SRD5A2,SRSF7,STON1,STON1-GTF2A1L,STRN,SULT6B1,SUPT7L,TAF1B,TCF23,TDRD15,THADA,THUMPD2,TMEM18,TMEM178A,TMEM214,TMEM247,TP53I3,TPO,TRAPPC12,TRIB2,TRIM54,TRMT61B,TSSC1,TTC7A,TTC27,TTC32,UBXN2A,UCN,VIT,VSNL1,WDCP,WDR35,WDR43,XDH,YIPF4,YPEL5,YWHAQ,ZFP36L2,ZNF512,ZNF513 | 261 |
| [5:68878193-70358590](genomebrowse://api/zoom?locus=5:68878193-70358590) | 62 | 1 | Duplicate | GTF2H2,GTF2H2C,NAIP,SERF1A,SERF1B,SMN1,SMN2 | 7 |  |
| [8:33361266-33455033](genomebrowse://api/zoom?locus=8:33361266-33455033) | 13 | 1 | Het Deletion | | DUSP26,RNF122,TTI2 | 3 |
| [9:21481067-23762232](genomebrowse://api/zoom?locus=9:21481067-23762232) | 24 | 1 | Het Deletion | | CDKN2A,CDKN2B,DMRTA1,ELAVL2,IFNE,MTAP | 6 |
| [9:26842299-27284970](genomebrowse://api/zoom?locus=9:26842299-27284970) | 68 | 1 | Het Deletion | | CAAP1,EQTN,IFT74,LRRC19,PLAA,TEK | 6 |
| [10:92997-135379033](genomebrowse://api/zoom?locus=10:92997-135379033) | 8110 | 1 | Het Deletion | | A1CF,ABCC2,ABI1,ABLIM1,ACADSB,ACBD5,ACBD7,ACSL5,ACSM6,ACTA2,ACTR1A,ADAM8,ADAM12,ADAMTS14,ADARB2,ADD3,ADGRA1,ADIRF,ADK,ADO,ADRA2A,ADRB1,AFAP1L2,AGAP4,AGAP5,AGAP6,AGAP9,AGAP11,AIFM2,AKR1C1,AKR1C2,AKR1C3,AKR1C4,AKR1E2,ALDH18A1,ALOX5,ANAPC16,ANK3,ANKRD1,ANKRD2,ANKRD16,ANKRD22,ANKRD26,ANKRD30A,ANTXRL,ANXA7,ANXA8,ANXA8L1,ANXA11,AP3M1,APBB1IP,ARHGAP12,ARHGAP19,ARHGAP21,ARHGAP22,ARID5B,ARL3,ARL5B,ARMC3,ARMC4,ARMS2,AS3MT,ASAH2,ASAH2B,ASB13,ASCC1,ATAD1,ATE1,ATOH7,ATP5C1,ATRNL1,AVPI1,BAG3,BAMBI,BBIP1,BCCIP,BEND7,BICC1,BLNK,BLOC1S2,BMI1,BMPR1A,BMS1,BNIP3,BORCS7,BTAF1,BTBD16,BTRC,BUB3,C1QL3,C10orf10,C10orf11,C10orf25,C10orf35,C10orf53,C10orf55,C10orf62,C10orf67,C10orf71,C10orf76,C10orf82,C10orf88,C10orf90,C10orf91,C10orf95,C10orf99,C10orf105,C10orf107,C10orf111,C10orf113,C10orf120,C10orf126,C10orf128,C10orf131,C10orf142,CACNB2,CACUL1,CALHM1,CALHM2,CALHM3,CALML3,CALML5,CALY,CAMK1D,CAMK2G,CASC10,CASP7,CC2D2B,CCAR1,CCDC3,CCDC6,CCDC7,CCDC172,CCDC186,CCNJ,CCNY,CCSER2,CDC123,CDH23,CDHR1,CDK1,CDNF,CELF2,CEP55,CFAP43,CFAP46,CFAP58,CFAP70,CH25H,CHAT,CHCHD1,CHST3,CHST15,CHUK,CISD1,CLRN3,CNNM1,CNNM2,COL13A1,COL17A1,COMMD3,COMMD3-BMI1,COMTD1,COX15,CPEB3,CPN1,CPXM2,CREM,CRTAC1,CSGALNACT2,CSTF2T,CTBP2,CTNNA3,CUBN,CUEDC2,CUL2,CUTC,CUZD1,CWF19L1,CXCL12,CYP2C8,CYP2C9,CYP2C18,CYP2C19,CYP2E1,CYP17A1,CYP26A1,CYP26C1,DCLRE1A,DCLRE1C,DDIT4,DDX21,DDX50,DHTKD1,DHX32,DIP2C,DKK1,DLG5,DMBT1,DNA2,DNAJB12,DNAJC1,DNAJC9,DNAJC12,DNMBP,DNTT,DOCK1,DPCD,DPYSL4,DRGX,DUPD1,DUSP5,DUSP13,DYDC1,DYDC2,EBF3,EBLN1,ECD,ECHDC3,ECHS1,EDRF1,EGR2,EIF3A,EIF4EBP2,EIF5AL1,ELOVL3,EMX2,ENKUR,ENO4,ENTPD1,ENTPD7,EPC1,ERCC6,ERLIN1,EXOC6,EXOSC1,FAM13C,FAM24A,FAM24B,FAM25A,FAM25C,FAM25G,FAM35A,FAM45A,FAM53B,FAM107B,FAM149B1,FAM160B1,FAM170B,FAM171A1,FAM175B,FAM188A,FAM196A,FAM204A,FAM208B,FAM213A,FANK1,FAS,FBXL15,FBXO18,FBXW4,FFAR4,FGF8,FGFBP3,FGFR2,FOXI2,FRA10AC1,FRAT1,FRAT2,FRMD4A,FRMPD2,FUOM,FUT11,FXYD4,FZD8,GAD2,GATA3,GBF1,GDF2,GDF10,GDI2,GFRA1,GHITM,GJD4,GLRX3,GLUD1,GOLGA7B,GOT1,GPAM,GPR26,GPR158,GPRIN2,GRID1,GRK5,GSTO1,GSTO2,GTPBP4,H2AFY2,HABP2,HACD1,HECTD2,HELLS,HERC4,HHEX,HIF1AN,HK1,HKDC1,HMX2,HMX3,HNRNPF,HNRNPH3,HOGA1,HPS1,HPS6,HPSE2,HSPA12A,HSPA14,HTR7,HTRA1,IDE,IDI1,IDI2,IFIT1,IFIT1B,IFIT2,IFIT3,IFIT5,IKZF5,IL2RA,IL15RA,INA,INPP5A,INPP5F,IPMK,ITGA8,ITGB1,ITIH2,ITIH5,ITPRIP,JAKMIP3,JMJD1C,KAT6B,KAZALD1,KCNIP2,KCNK18,KCNMA1,KIAA1217,KIAA1462,KIF1BP,KIF5B,KIF11,KIF20B,KIN,KLF6,KLLN,KNDC1,LARP4B,LBX1,LCOR,LDB1,LDB3,LGI1,LHPP,LIPA,LIPF,LIPJ,LIPK,LIPM,LIPN,LOXL4,LRIT1,LRIT2,LRRC18,LRRC20,LRRC27,LRRTM3,LYZL1,LYZL2,LZTS2,MALRD1,MAP3K8,MAPK8,MARCH5,MARCH8,MARVELD1,MASTL,MAT1A,MBL2,MCM10,MCMBP,MCU,MEIG1,METTL10,MFSD13A,MGEA5,MGMT,MICU1,MINPP1,MKI67,MKX,MLLT10,MMP21,MMRN2,MMS19,MORN4,MPP7,MRC1,MRLN,MRPL43,MRPS16,MSMB,MSRB2,MSS51,MTG1,MTPAP,MTRNR2L5,MTRNR2L7,MXI1,MYO3A,MYOF,MYOZ1,MYPN,NANOS1,NCOA4,NDST2,NDUFB8,NEBL,NET1,NEURL1,NEUROG3,NFKB2,NHLRC2,NKX1-2,NKX2-3,NKX6-2,NMT2,NOC3L,NODAL,NOLC1,NPFFR1,NPM3,NPS,NPY4R,NRAP,NRBF2,NRG3,NRP1,NSMCE4A,NSUN6,NT5C2,NUDT5,NUDT13,NUTM2A,NUTM2B,OAT,OGDHL,OIT3,OLAH,OPALIN,OPN4,OPTN,OR13A1,OTUD1,P4HA1,PALD1,PANK1,PAOX,PAPSS2,PARD3,PARG,PAX2,PBLD,PCBD1,PCDH15,PCGF5,PCGF6,PDCD4,PDCD11,PDE6C,PDLIM1,PDSS1,PDZD7,PDZD8,PFKFB3,PFKP,PGAM1,PGBD3,PHYH,PHYHIPL,PI4K2A,PIK3AP1,PIP4K2A,PITRM1,PITX3,PKD2L1,PLA2G12B,PLAC9,PLAU,PLCE1,PLEKHA1,PLEKHS1,PLPP4,PLXDC2,PNLIP,PNLIPRP1,PNLIPRP2,PNLIPRP3,POLL,POLR3A,PPA1,PPIF,PPP1R3C,PPP2R2D,PPP3CB,PPRC1,PRAP1,PRDX3,PRF1,PRKCQ,PRKG1,PRLHR,PROSER2,PRPF18,PRTFDC1,PSAP,PSD,PSTK,PTCHD3,PTEN,PTER,PTF1A,PTPN20,PTPRE,PWWP2B,PYROXD2,R3HCC1L,RAB11FIP2,RAB18,RASGEF1A,RASSF4,RBM17,RBM20,RBP3,RBP4,REEP3,RET,RGR,RGS10,RHOBTB1,RNLS,RPEL1,RPP30,RPP38,RPS24,RRP12,RSU1,RTKN2,RUFY2,SAMD8,SAR1A,SCD,SEC23IP,SEC24C,SEC31B,SEC61A2,SEMA4G,SEPHS1,SFMBT2,SFR1,SFRP5,SFTPA1,SFTPA2,SFTPD,SFXN2,SFXN3,SFXN4,SGMS1,SGPL1,SH2D4B,SH3PXD2A,SHOC2,SHTN1,SIRT1,SKIDA1,SLC16A9,SLC16A12,SLC18A2,SLC18A3,SLC25A16,SLC25A28,SLC29A3,SLC35G1,SLC39A12,SLF2,SLIT1,SLK,SMC3,SMNDC1,SNCG,SORBS1,SORCS1,SORCS3,SPAG6,SPOCK2,SPRN,SRGN,ST8SIA6,STAM,STAMBPL1,STK32C,STN1,STOX1,SUFU,SUPV3L1,SUV39H2,SVIL,SYCE1,SYNPO2L,SYT15,TACC2,TACR2,TAF3,TAF5,TBATA,TBC1D12,TCERG1L,TCF7L2,TCTN3,TDRD1,TECTB,TET1,TEX36,TFAM,THNSL1,TIAL1,TIMM23,TIMM23B,TLL2,TLX1,TM9SF3,TMEM26,TMEM72,TMEM236,TMEM254,TNKS2,TRDMT1,TRIM8,TRUB1,TSPAN14,TSPAN15,TUBAL3,TUBB8,TUBGCP2,TWNK,TYSND1,UBE2D1,UBTD1,UCMA,UCN3,UNC5B,UPF2,UROS,USMG5,USP6NL,USP54,UTF1,VAX1,VCL,VDAC2,VENTX,VIM,VPS26A,VSIR,VSTM4,VTI1A,VWA2,WAC,WAPL,WASHC2A,WASHC2C,WBP1L,WDFY4,WDR11,WDR37,WNT8B,XPNPEP1,YME1L1,ZCCHC24,ZDHHC6,ZDHHC16,ZEB1,ZFAND4,ZFYVE27,ZMIZ1,ZMYND11,ZNF22,ZNF25,ZNF32,ZNF33A,ZNF33B,ZNF37A,ZNF239,ZNF248,ZNF365,ZNF438,ZNF485,ZNF488,ZNF503,ZNF511,ZNF518A,ZRANB1,ZSWIM8,ZWINT | 713 |
| [12:58174038-58204334](genomebrowse://api/zoom?locus=12:58174038-58204334) | 23 | 2 | Het Deletion | | AVIL,METTL21B,TSFM | 3 |
| [12:58240155-59314016](genomebrowse://api/zoom?locus=12:58240155-59314016) | 28 | 1 | Het Deletion | | ATP23,CTDSP2,LRIG3 | 3 |
| [13:19748003-115091756](genomebrowse://api/zoom?locus=13:19748003-115091756) | 3474 | 2 | Het Deletion | | ABCC4,ABHD13,ACOD1,ADPRHL1,AKAP11,ALG5,ALG11,ALOX5AP,AMER2,ANKRD10,ARGLU1,ARHGEF7,ARL11,ATP4B,ATP7B,ATP8A2,ATP11A,ATP11AUN,ATP12A,B3GLCT,BIVM,BIVM-ERCC5,BORA,BRCA2,C1QTNF9,C1QTNF9B,C1QTNF9B-AS1,CAB39L,CARS2,CCDC70,CCDC122,CCDC168,CCDC169,CCDC169-SOHLH2,CCNA1,CDADC1,CDC16,CDK8,CDX2,CENPJ,CHAMP1,CKAP2,CLDN10,CLN5,CLYBL,CNMD,COG3,COG6,COL4A1,COL4A2,COL4A2-AS2,COMMD6,CPB2,CRYL1,CSNK1A1L,CUL4A,CYSLTR2,DACH1,DAOA,DCLK1,DCT,DCUN1D2,DGKH,DHRS12,DIAPH3,DIS3,DLEU7,DNAJC3,DNAJC15,DOCK9,DZIP1,EBPL,EDNRB,EEF1AKMT1,EFNB2,ELF1,ENOX1,EPSTI1,ERCC5,ERICH6B,ESD,EXOSC8,F7,F10,FAM124A,FAM155A,FAM216B,FARP1,FBXL3,FGF9,FGF14,FLT1,FLT3,FNDC3A,FOXO1,FREM2,FRY,GAS6,GGACT,GJA3,GJB2,GJB6,GPALPP1,GPC5,GPC6,GPR12,GPR18,GPR180,GPR183,GRK1,GRTP1,GSX1,GTF2F2,GTF3A,HMGB1,HNRNPA1L2,HS6ST3,HSPH1,HTR2A,IFT88,IL17D,ING1,INTS6,IPO5,IRS2,ITGBL1,ITM2B,KATNAL1,KBTBD6,KBTBD7,KCNRG,KCTD4,KCTD12,KDELC1,KL,KLF5,KLF12,KLHL1,KPNA3,LACC1,LAMP1,LATS2,LCP1,LHFP,LIG4,LINC00452,LMO7,LMO7DN,LNX2,LOC100129307,LOC101928841,LPAR6,LRCH1,LRRC63,MAB21L1,MBNL2,MCF2L,MED4,MEDAG,METTL21C,MICU2,MIPEP,MLNR,MPHOSPH8,MRPL57,MRPS31,MTIF3,MTMR6,MTRF1,MTUS2,MYCBP2,MYO16,MZT1,N4BP2L1,N4BP2L2,NAA16,NALCN,NAXD,NBEA,NDFIP2,NEK3,NEK5,NHLRC3,NUDT15,NUFIP1,NUP58,OLFM4,OXGR1,PABPC3,PAN3,PARP4,PCCA,PCDH8,PCDH9,PCDH17,PCDH20,PCID2,PDS5B,PDX1,PHF11,PIBF1,POLR1D,POMP,POSTN,POU4F1,PROSER1,PROZ,PRR20A,PRR20B,PRR20C,PRR20D,PRR20E,PSPC1,RAB20,RAP2A,RASA3,RASL11A,RB1,RBM26,RCBTB1,RCBTB2,RFC3,RFXAP,RGCC,RNASEH2B,RNF6,RNF17,RNF113B,RNF219,RPL21,RUBCNL,RXFP2,SACS,SAP18,SCEL,SERP2,SERPINE3,SERTM1,SETDB2,SETDB2-PHF11,SGCG,SHISA2,SIAH3,SKA3,SLAIN1,SLC7A1,SLC10A2,SLC15A1,SLC25A15,SLC25A30,SLC46A3,SLITRK1,SLITRK5,SLITRK6,SMAD9,SMIM2,SOHLH2,SOX1,SOX21,SPACA7,SPATA13,SPERT,SPG20,SPRY2,SPRYD7,STARD13,STK24,STOML3,SUCLA2,SUGT1,SUPT20H,TBC1D4,TDRD3,TEX26,TEX29,TEX30,TFDP1,TGDS,THSD1,TM9SF2,TMCO3,TMEM255B,TMTC4,TNFRSF19,TNFSF11,TNFSF13B,TPP2,TPT1,TPTE2,TRIM13,TRPC4,TSC22D1,TUBA3C,TUBGCP3,UBAC2,UBL3,UCHL3,UFM1,UGGT2,UPF3A,URAD,USP12,USPL1,UTP14C,VPS36,VWA8,WASF3,WBP4,WDFY2,XPO4,ZAR1L,ZC3H13,ZDHHC20,ZIC2,ZIC5,ZMYM2,ZMYM5 | 317 |
| [14:20443678-25444024](genomebrowse://api/zoom?locus=14:20443678-25444024) | 1219 | 1 | Het Deletion | | ABHD4,ACIN1,ADCY4,AJUBA,ANG,AP1G2,APEX1,ARHGEF40,BCL2L2,BCL2L2-PABPN1,C14orf93,C14orf119,CARMIL3,CBLN3,CCNB1IP1,CDH24,CEBPE,CHD8,CHMP4A,CIDEB,CMA1,CMTM5,CPNE6,CTSG,DAD1,DCAF11,DHRS1,DHRS2,DHRS4,DHRS4L1,DHRS4L2,EDDM3A,EDDM3B,EFS,EMC9,FITM1,GMPR2,GZMB,GZMH,HAUS4,HNRNPC,HOMEZ,IL25,IPO4,IRF9,JPH4,KHNYN,KLHL33,LRP10,LTB4R,LTB4R2,MDP1,METTL3,METTL17,MMP14,MRPL52,MYH6,MYH7,NDRG2,NEDD8,NEDD8-MDP1,NFATC4,NGDN,NOP9,NRL,NYNRIN,OR4E1,OR4E2,OR4K13,OR4K14,OR4K15,OR4K17,OR4L1,OR4N5,OR5AU1,OR6S1,OR10G2,OR10G3,OR11G2,OR11H4,OR11H6,OSGEP,OXA1L,PABPN1,PARP2,PCK2,PNP,PPP1R3E,PRMT5,PSMB5,PSMB11,PSME1,PSME2,RAB2B,RABGGTA,RBM23,REC8,REM2,RIPK3,RNASE1,RNASE2,RNASE3,RNASE4,RNASE6,RNASE7,RNASE8,RNASE9,RNASE10,RNASE11,RNASE12,RNASE13,RNF31,RNF212B,RPGRIP1,SALL2,SDR39U1,SLC7A7,SLC7A8,SLC22A17,SLC39A2,STXBP6,SUPT16H,TEP1,TGM1,THTPA,TINF2,TM9SF1,TMEM55B,TMEM253,TOX4,TPPP2,TSSK4,TTC5,ZFHX2,ZNF219 | 135 |
| [16:14782022-15112822](genomebrowse://api/zoom?locus=16:14782022-15112822) | 70 | 1 | Duplicate | NOMO1,NPIPA1,NPIPA2,NPIPA3,PDXDC1,PLA2G10 | 6 |  |
| [17:6328780-18389449](genomebrowse://api/zoom?locus=17:6328780-18389449) | 2028 | 1 | Het Deletion | | ACADVL,ACAP1,ADORA2B,ADPRM,AIPL1,ALKBH5,ALOX12,ALOX12B,ALOX15B,ALOXE3,ARHGAP44,ARHGEF15,ASGR1,ASGR2,ATP1B2,ATPAF2,AURKB,BCL6B,BORCS6,C17orf49,C17orf74,C17orf100,CCDC42,CCDC144A,CD68,CDRT1,CDRT4,CDRT15,CENPV,CFAP52,CHD3,CHRNB1,CLDN7,CLEC10A,CNTROB,COPS3,COX10,CTC1,CTDNEP1,CYB5D1,DHRS7C,DLG4,DNAH2,DNAH9,DRC3,DRG2,DVL2,EFNB3,EIF4A1,EIF5A,ELAC2,ELP5,EVPLL,FAM64A,FAM106CP,FBXO39,FGF11,FLCN,FLII,FXR2,GABARAP,GAS7,GID4,GLP2R,GPS2,GSG1L2,GUCY2D,HES7,HS3ST3A1,HS3ST3B1,KCNAB3,KCTD11,KDM6B,KIAA0753,KRBA2,LGALS9C,LLGL1,LOC100996842,LRRC75A,MAP2K4,MED9,MED31,MFSD6L,MIEF2,MPDU1,MPRIP,MYH1,MYH2,MYH3,MYH4,MYH8,MYH10,MYH13,MYO15A,MYOCD,NAA38,NCOR1,NDEL1,NEURL4,NLGN2,NT5M,NTN1,ODF4,PEMT,PER1,PFAS,PHF23,PIGL,PIK3R5,PIK3R6,PIRT,PITPNM3,PLD6,PLSCR3,PMP22,POLR2A,RAI1,RANGRF,RASD1,RCVRN,RNASEK,RNF222,RPL26,SAT2,SCO1,SENP3,SHBG,SHISA6,SHMT1,SLC2A4,SLC13A5,SLC16A11,SLC16A13,SLC25A35,SLC35G6,SMCR8,SOX15,SPDYE4,SPEM1,SREBF1,STX8,TBC1D26,TEKT1,TEKT3,TMEM88,TMEM95,TMEM102,TMEM107,TMEM220,TMEM256,TNFRSF13B,TNFSF12,TNFSF12-TNFSF13,TNFSF13,TNK1,TOM1L2,TOP3A,TP53,TRAPPC1,TRIM16,TRPV2,TTC19,TVP23C,TVP23C-CDRT4,TXNDC17,UBB,USP43,VAMP2,WRAP53,XAF1,YBX2,ZBTB4,ZNF18,ZNF286A,ZNF287,ZNF624,ZSWIM7 | 177 |
| [17:76991100-77111797](genomebrowse://api/zoom?locus=17:76991100-77111797) | 30 | 1 | Duplicate | C1QTNF1,CANT1,ENGASE,RBFOX3 | 4 |  |
| [17:79257209-79358931](genomebrowse://api/zoom?locus=17:79257209-79358931) | 10 | 1 | Duplicate | LOC100130370,SLC38A10,TMEM105 | 3 |  |
| [17:79405395-79419877](genomebrowse://api/zoom?locus=17:79405395-79419877) | 12 | 1 | Duplicate | BAHCC1 | 1 |  |
| [20:20552102-20624193](genomebrowse://api/zoom?locus=20:20552102-20624193) | 19 | 2 | Het Deletion | | RALGAPA2 | 1 |

Lesion

| [1:12939365-13717379](genomebrowse://api/zoom?locus=1:12939365-13717379) | 41 | 2 | Duplicate | HNRNPCL2,PRAMEF4,PRAMEF5,PRAMEF6,PRAMEF7,PRAMEF8,PRAMEF10,PRAMEF13,PRAMEF14,PRAMEF15,PRAMEF17,PRAMEF18,PRAMEF19,PRAMEF25,PRAMEF33P | 15 |
| --- | --- | --- | --- | --- | --- |
| [1:104094343-104205397](genomebrowse://api/zoom?locus=1:104094343-104205397) | 29 | 1 | Duplicate | AMY1A,AMY2A,AMY2B,RNPC3 | 4 |
| [1:145748338-145816740](genomebrowse://api/zoom?locus=1:145748338-145816740) | 19 | 3 | Duplicate | GPR89A,PDZK1 | 2 |
| [2:89553027-89891301](genomebrowse://api/zoom?locus=2:89553027-89891301) | 10 | 1 | Duplicate |  |  |
| [2:97879156-97914951](genomebrowse://api/zoom?locus=2:97879156-97914951) | 15 | 3 | Duplicate | ANKRD36 | 1 |
| [2:242650792-242716399](genomebrowse://api/zoom?locus=2:242650792-242716399) | 18 | 1 | Duplicate | D2HGDH,GAL3ST2,ING5 | 3 |
| [7:142419212-142494975](genomebrowse://api/zoom?locus=7:142419212-142494975) | 17 | 1 | Duplicate | PRSS1 | 1 |
| [15:22051872-22743598](genomebrowse://api/zoom?locus=15:22051872-22743598) | 21 | 1 | Duplicate | GOLGA6L1,OR4M2,OR4N4,POTEB | 4 |
| [15:30654496-30706365](genomebrowse://api/zoom?locus=15:30654496-30706365) | 27 | 1 | Duplicate | CHRFAM7A,GOLGA8R | 2 |
| [15:32739198-32908541](genomebrowse://api/zoom?locus=15:32739198-32908541) | 34 | 1 | Duplicate | ARHGAP11A,GOLGA8N,GOLGA8O | 3 |
| [16:18535811-18569113](genomebrowse://api/zoom?locus=16:18535811-18569113) | 16 | 1 | Duplicate | NOMO2 | 1 |
| [16:30200181-30365643](genomebrowse://api/zoom?locus=16:30200181-30365643) | 29 | 1 | Duplicate | BOLA2B,CD2BP2,CORO1A,NPIPB13,SLX1A,SULT1A3 | 6 |

| **Region** | **# Targets** | **# Samples** | **CNV State** | **CNV State** | **Flags** |
| --- | --- | --- | --- | --- | --- |
| [1:13000814-13646345](genomebrowse://api/zoom?locus=1:13000814-13646345) | 22 | 2 | Duplicate | HNRNPCL2,PRAMEF5,PRAMEF6,PRAMEF8,PRAMEF13,PRAMEF15,PRAMEF18,PRAMEF25,PRAMEF33P | 9 |
| [1:145304447-145316684](genomebrowse://api/zoom?locus=1:145304447-145316684) | 11 | 1 | Duplicate | NBPF10 | 1 |
| [5:70248266-70308742](genomebrowse://api/zoom?locus=5:70248266-70308742) | 15 | 1 | Duplicate | NAIP,SMN1 | 2 |
| [7:142423216-142494975](genomebrowse://api/zoom?locus=7:142423216-142494975) | 16 | 1 | Duplicate | PRSS1 | 1 |
| [15:75576991-75586816](genomebrowse://api/zoom?locus=15:75576991-75586816) | 17 | 1 | Duplicate | GOLGA6D | 1 |
| [16:14782022-15091683](genomebrowse://api/zoom?locus=16:14782022-15091683) | 60 | 1 | Duplicate | NOMO1,NPIPA1,NPIPA2,NPIPA3,PDXDC1,PLA2G10 | 6 |
| [16:28659456-28834879](genomebrowse://api/zoom?locus=16:28659456-28834879) | 35 | 1 | Duplicate | ATXN2L,EIF3C,NPIPB8,NPIPB9 | 4 |
| [16:30200181-30365121](genomebrowse://api/zoom?locus=16:30200181-30365121) | 27 | 2 | Duplicate | BOLA2B,CD2BP2,CORO1A,NPIPB13,SLX1A,SULT1A3 | 6 |
| [17:16614993-16843111](genomebrowse://api/zoom?locus=17:16614993-16843111) | 15 | 1 | Duplicate | CCDC144A,FAM106CP,TNFRSF13B | 3 |
| [X:134930172-134967496](genomebrowse://api/zoom?locus=X:134930172-134967496) | 11 | 2 | Duplicate | CT45A8,CT45A9,CT45A10 | 3 |

Lesion:

| **Region** | **# Targets** | **# Samples** | **CNV State** | **CNV State** | **Flags** |  |
| --- | --- | --- | --- | --- | --- | --- |
| [1:12939365-13717379](genomebrowse://api/zoom?locus=1:12939365-13717379) | 41 | 2 | Duplicate | HNRNPCL2,PRAMEF4,PRAMEF5,PRAMEF6,PRAMEF7,PRAMEF8,PRAMEF10,PRAMEF13,PRAMEF14,PRAMEF15,PRAMEF17,PRAMEF18,PRAMEF19,PRAMEF25,PRAMEF33P | 15 |  |
| [1:104114225-104166843](genomebrowse://api/zoom?locus=1:104114225-104166843) | 19 | 1 | Duplicate | AMY2A,AMY2B | 2 |  |
| [2:107050684-107069382](genomebrowse://api/zoom?locus=2:107050684-107069382) | 11 | 1 | Duplicate | RGPD3 | 1 |  |
| [5:68830521-70358590](genomebrowse://api/zoom?locus=5:68830521-70358590) | 77 | 1 | Duplicate | GTF2H2,GTF2H2C,NAIP,OCLN,SERF1A,SERF1B,SMN1,SMN2 | 8 |  |
| [8:33364747-33455033](genomebrowse://api/zoom?locus=8:33364747-33455033) | 12 | 1 | Het Deletion | | DUSP26,RNF122,TTI2 | 3 |
| [8:67380504-67525073](genomebrowse://api/zoom?locus=8:67380504-67525073) | 23 | 1 | Duplicate | ADHFE1,C8orf46,MYBL1 | 3 |  |
| [16:18511694-18573362](genomebrowse://api/zoom?locus=16:18511694-18573362) | 31 | 1 | Duplicate | NOMO2 | 1 |  |
| [16:30200181-30365121](genomebrowse://api/zoom?locus=16:30200181-30365121) | 27 | 2 | Duplicate | BOLA2B,CD2BP2,CORO1A,NPIPB13,SLX1A,SULT1A3 | 6 |  |
| [20:20552102-20624193](genomebrowse://api/zoom?locus=20:20552102-20624193) | 19 | 2 | Het Deletion | | RALGAPA2 | 1 |

GBM-5

Blood

| **Region** | **# Targets** | **# Samples** | **CNV State** | **Gene Names** | **# Genes** |  |
| --- | --- | --- | --- | --- | --- | --- |
| [1:12888357-13745175](genomebrowse://api/zoom?locus=1:12888357-13745175) | 50 | 1 | Duplicate | HNRNPCL1,HNRNPCL2,PRAMEF2,PRAMEF4,PRAMEF5,PRAMEF6,PRAMEF7,PRAMEF8,PRAMEF10,PRAMEF11,PRAMEF13,PRAMEF14,PRAMEF15,PRAMEF17,PRAMEF18,PRAMEF19,PRAMEF20,PRAMEF25,PRAMEF33P | 19 |  |
| [1:45363027-45811666](genomebrowse://api/zoom?locus=1:45363027-45811666) | 76 | 1 | Duplicate | EIF2B3,HECTD3,HPDL,MUTYH,TESK2,TOE1,UROD,ZSWIM5 | 8 |  |
| [1:145304447-145315899](genomebrowse://api/zoom?locus=1:145304447-145315899) | 10 | 2 | Duplicate | NBPF10 | 1 |  |
| [1:152636582-153177480](genomebrowse://api/zoom?locus=1:152636582-153177480) | 27 | 1 | Duplicate | C1orf68,IVL,KPRP,LCE1A,LCE1B,LCE1C,LCE1D,LCE1E,LCE1F,LCE2A,LCE2B,LCE2C,LCE2D,LCE4A,LCE6A,LELP1,SMCP,SPRR1A,SPRR1B,SPRR2A,SPRR2B,SPRR2D,SPRR2E,SPRR2F,SPRR2G,SPRR3,SPRR4 | 27 |  |
| [2:87144754-88118142](genomebrowse://api/zoom?locus=2:87144754-88118142) | 51 | 2 | Duplicate | PLGLB1,PLGLB2,RGPD1,RGPD2 | 4 |  |
| [2:131350431-131415450](genomebrowse://api/zoom?locus=2:131350431-131415450) | 21 | 2 | Duplicate | CFC1,POTEJ | 2 |  |
| [5:68830521-69366578](genomebrowse://api/zoom?locus=5:68830521-69366578) | 31 | 1 | Duplicate | GTF2H2C,OCLN,SERF1B,SMN2 | 4 |  |
| [6:32009126-32012493](genomebrowse://api/zoom?locus=6:32009126-32012493) | 12 | 5 | Duplicate | CYP21A2,TNXB | 2 |  |
| [7:102125486-102307711](genomebrowse://api/zoom?locus=7:102125486-102307711) | 65 | 2 | Duplicate | POLR2J2,POLR2J3,RASA4,RASA4B,SPDYE2,SPDYE2B,UPK3BL | 7 |  |
| [9:42406567-65508193](genomebrowse://api/zoom?locus=9:42406567-65508193) | 49 | 1 | Duplicate | ANKRD20A2,ANKRD20A3,CNTNAP3B,SPATA31A6,SPATA31A7 | 5 |  |
| [10:48739388-49389051](genomebrowse://api/zoom?locus=10:48739388-49389051) | 22 | 2 | Duplicate | FAM25C,FRMPD2,PTPN20 | 3 |  |
| [15:30375256-30706365](genomebrowse://api/zoom?locus=15:30375256-30706365) | 46 | 3 | Duplicate | CHRFAM7A,GOLGA8J,GOLGA8R | 3 |  |
| [15:30902969-30922976](genomebrowse://api/zoom?locus=15:30902969-30922976) | 10 | 2 | Duplicate | ARHGAP11B,GOLGA8H | 2 |  |
| [15:43864967-43892880](genomebrowse://api/zoom?locus=15:43864967-43892880) | 34 | 4 | Duplicate | CKMT1B,PPIP5K1,STRC | 3 |  |
| [15:72948853-72958469](genomebrowse://api/zoom?locus=15:72948853-72958469) | 16 | 3 | Duplicate | GOLGA6B | 1 |  |
| [15:74366834-74373076](genomebrowse://api/zoom?locus=15:74366834-74373076) | 10 | 1 | Duplicate | GOLGA6A | 1 |  |
| [15:75555689-75580320](genomebrowse://api/zoom?locus=15:75555689-75580320) | 20 | 1 | Duplicate | GOLGA6C,GOLGA6D | 2 |  |
| [16:14782022-15044112](genomebrowse://api/zoom?locus=16:14782022-15044112) | 57 | 1 | Duplicate | NOMO1,NPIPA1,NPIPA2,NPIPA3,PLA2G10 | 5 |  |
| [16:18511694-18569113](genomebrowse://api/zoom?locus=16:18511694-18569113) | 30 | 2 | Duplicate | NOMO2 | 1 |  |
| [16:28659456-28784076](genomebrowse://api/zoom?locus=16:28659456-28784076) | 34 | 2 | Duplicate | EIF3C,NPIPB8,NPIPB9 | 3 |  |
| [17:10347921-10357202](genomebrowse://api/zoom?locus=17:10347921-10357202) | 17 | 5 | Het Deletion | | MYH4 | 1 |

Lesion:

| **Region** | **# Targets** | **# Samples** | **CNV State** | **CNV State** | **Flags** |  |
| --- | --- | --- | --- | --- | --- | --- |
| [1:12954417-13646345](genomebrowse://api/zoom?locus=1:12954417-13646345) | 28 | 1 | Duplicate | HNRNPCL2,PRAMEF5,PRAMEF6,PRAMEF7,PRAMEF8,PRAMEF10,PRAMEF13,PRAMEF15,PRAMEF18,PRAMEF25,PRAMEF33P | 11 |  |
| [6:31962729-31996054](genomebrowse://api/zoom?locus=6:31962729-31996054) | 44 | 2 | Duplicate | C4A,C4B | 2 |  |
| [9:21802748-22008952](genomebrowse://api/zoom?locus=9:21802748-22008952) | 15 | 3 | Het Deletion | | CDKN2A,CDKN2B,MTAP | 3 |
| [9:41500708-43920370](genomebrowse://api/zoom?locus=9:41500708-43920370) | 62 | 1 | Duplicate | ANKRD20A2,ANKRD20A3,CNTNAP3B,SPATA31A5,SPATA31A6 | 5 |  |
| [15:43902510-43910920](genomebrowse://api/zoom?locus=15:43902510-43910920) | 15 | 2 | Duplicate | STRC | 1 |  |
| [X:49161883-49361336](genomebrowse://api/zoom?locus=X:49161883-49361336) | 59 | 2 | Duplicate | GAGE2A,GAGE2B,GAGE2C,GAGE2D,GAGE10,GAGE12B,GAGE12C,GAGE12D,GAGE12E,GAGE12F,GAGE12G,GAGE12H,GAGE12I,GAGE12J,GAGE13 | 15 |  |

Lesion:

| **Region** | **# Targets** | **# Samples** | **CNV State** | **CNV State** | **Flags** |  |
| --- | --- | --- | --- | --- | --- | --- |
| [1:145304447-145316684](genomebrowse://api/zoom?locus=1:145304447-145316684) | 11 | 1 | Duplicate | NBPF10 | 1 |  |
| [6:31962729-31996054](genomebrowse://api/zoom?locus=6:31962729-31996054) | 44 | 2 | Duplicate | C4A,C4B | 2 |  |
| [7:208898-158937463](genomebrowse://api/zoom?locus=7:208898-158937463) | 9334 | 1 | Duplicate | AASS,ABCA13,ABCB1,ABCB4,ABCB5,ABCB8,ABCF2,ABHD11,ACHE,ACTB,ACTL6B,ACTR3B,ACTR3C,ADAM22,ADAP1,ADCK2,ADCY1,ADCYAP1R1,AEBP1,AGAP3,AGBL3,AGFG2,AGK,AGMO,AGR2,AGR3,AHCYL2,AHR,AIMP2,AKAP9,AKR1B1,AKR1B10,AKR1B15,AKR1D1,ALKBH4,AMPH,AMZ1,ANKIB1,ANKMY2,ANKRD7,ANKRD61,ANLN,AOAH,AOC1,AP1S1,AP4M1,AP5Z1,AQP1,ARF5,ARHGEF5,ARHGEF35,ARL4A,ARMC10,ARPC1A,ARPC1B,ASB4,ASB10,ASB15,ASIC3,ASL,ASNS,ASZ1,ATG9B,ATP5J2,ATP5J2-PTCD1,ATP6V0A4,ATP6V0E2,ATP6V1F,ATXN7L1,AUTS2,AVL9,AZGP1,BAIAP2L1,BAZ1B,BBS9,BCAP29,BCL7B,BET1,BHLHA15,BLVRA,BMPER,BMT2,BPGM,BRAF,BRAT1,BRI3,BUD31,BZW2,C1GALT1,C7orf25,C7orf26,C7orf31,C7orf33,C7orf34,C7orf43,C7orf49,C7orf50,C7orf55-LUC7L2,C7orf57,C7orf61,C7orf62,C7orf65,C7orf66,C7orf69,C7orf71,C7orf72,C7orf73,C7orf76,C7orf77,CACNA2D1,CADPS2,CALCR,CALD1,CALN1,CALU,CAMK2B,CAPZA2,CARD11,CASD1,CASP2,CAV1,CAV2,CBLL1,CBX3,CCDC71L,CCDC126,CCDC129,CCDC136,CCDC146,CCL24,CCL26,CCM2,CCT6A,CCZ1,CCZ1B,CD36,CDCA7L,CDHR3,CDK5,CDK6,CDK13,CDK14,CEP41,CFAP69,CFTR,CHCHD2,CHCHD3,CHN2,CHPF2,CHRM2,CHST12,CLCN1,CLDN3,CLDN4,CLDN12,CLDN15,CLEC2L,CLEC5A,CLIP2,CNOT4,CNPY1,CNPY4,CNTNAP2,COA1,COBL,COG5,COL1A2,COL26A1,COL28A1,COPG2,COPS6,COX19,CPA1,CPA2,CPA4,CPA5,CPED1,CPSF4,CPVL,CRCP,CREB3L2,CREB5,CRHR2,CROT,CRYGN,CTAGE4,CTAGE6,CTAGE8,CTAGE15,CTTNBP2,CUL1,CUX1,CYCS,CYP2W1,CYP3A4,CYP3A5,CYP3A7,CYP3A7-CYP3A51P,CYP3A43,CYP51A1,CYTH3,DAGLB,DBF4,DBNL,DDC,DDX56,DENND2A,DFNA5,DGKB,DGKI,DLD,DLX5,DLX6,DMTF1,DNAAF5,DNAH11,DNAJB6,DNAJB9,DNAJC2,DNAJC30,DOCK4,DPP6,DPY19L1,DTX2,DUS4L,DYNC1I1,EEPD1,EGFR,EIF2AK1,EIF3B,EIF4H,ELFN1,ELMO1,ELN,EN2,EPDR1,EPHA1,EPHB4,EPHB6,EPO,ERV3-1,ERV3-1-ZNF117,ERVW-1,ESYT2,ETV1,EVX1,EXOC4,EZH2,FAM3C,FAM20C,FAM71F1,FAM71F2,FAM126A,FAM131B,FAM133B,FAM180A,FAM185A,FAM188B,FAM200A,FAM220A,FAM221A,FASTK,FBXL13,FBXL18,FBXO24,FERD3L,FEZF1,FGL2,FIGNL1,FIS1,FKBP6,FKBP9,FKBP14,FLNC,FMC1,FOXK1,FOXP2,FSCN1,FSCN3,FZD1,FZD9,GAL3ST4,GALNT11,GALNTL5,GARS,GATAD1,GATS,GATSL2,GBAS,GBX1,GCC1,GCK,GET4,GGCT,GHRHR,GIGYF1,GIMAP1,GIMAP1-GIMAP5,GIMAP2,GIMAP4,GIMAP5,GIMAP6,GIMAP7,GIMAP8,GJC3,GLCCI1,GLI3,GNA12,GNAI1,GNAT3,GNB2,GNG11,GNGT1,GPC2,GPER1,GPNMB,GPR22,GPR37,GPR85,GPR141,GPR146,GRB10,GRID2IP,GRIFIN,GRM3,GRM8,GS1-259H13.2,GSAP,GSTK1,GTF2I,GTF2IRD1,GTF2IRD2,GTF2IRD2B,GTPBP10,GUSB,H2AFV,HBP1,HDAC9,HECW1,HEPACAM2,HERPUD2,HGF,HIBADH,HILPDA,HIP1,HIPK2,HNRNPA2B1,HOXA1,HOXA2,HOXA3,HOXA4,HOXA5,HOXA6,HOXA7,HOXA9,HOXA10,HOXA11,HOXA13,HSPB1,HTR5A,HUS1,HYAL4,ICA1,IFRD1,IFT22,IGF2BP3,IGFBP1,IGFBP3,IKZF1,IL6,IMMP2L,IMPDH1,ING3,INHBA,INMT,INSIG1,INTS1,IQCA1L,IQCE,IQUB,IRF5,ISPD,ITGB8,JAZF1,KBTBD2,KCND2,KCNH2,KCP,KCTD7,KDELR2,KDM7A,KEL,KIAA0895,KIAA1147,KIAA1324L,KIAA1549,KLF14,KLHDC10,KLHL7,KLRG2,KMT2C,KMT2E,KPNA7,KRBA1,KRIT1,LAMB1,LAMB4,LAMTOR4,LANCL2,LAT2,LEP,LFNG,LHFPL3,LIMK1,LMBR1,LMOD2,LMTK2,LOC389602,LOC100130705,LOC100130880,LOC100289561,LOC100507507,LOC105375396,LRCH4,LRGUK,LRRC4,LRRC17,LRRC61,LRRC72,LRRD1,LRRN3,LRWD1,LSM5,LSM8,LSMEM1,LUC7L2,LUZP6,MACC1,MAD1L1,MAFK,MAGI2,MALSU1,MBLAC1,MCM7,MDFIC,MDH2,MEOX2,MEPCE,MEST,MET,METTL2B,MGAM,MGAM2,MICALL2,MIOS,MKLN1,MKRN1,MLXIPL,MMD2,MNX1,MOGAT3,MOSPD3,MPLKIP,MPP6,MRM2,MRPL32,MRPS17,MRPS24,MRPS33,MTERF1,MTPN,MTRNR2L6,MTURN,MUC3A,MUC12,MUC17,MYL7,MYL10,MYO1G,NACAD,NAMPT,NAPEPLD,NAT16,NCAPG2,NCF1,NDUFA4,NDUFA5,NDUFB2,NEUROD6,NFE2L3,NFE4,NME8,NOBOX,NOD1,NOM1,NOS3,NPC1L1,NPSR1,NPTX2,NPVF,NPY,NRCAM,NRF1,NSUN5,NT5C3A,NUB1,NUDCD3,NUDT1,NUP205,NUPL2,NUPR2,NXPH1,NYAP1,OCM,OCM2,OGDH,OPN1SW,OR2A1,OR2A2,OR2A5,OR2A7,OR2A12,OR2A14,OR2A25,OR2A42,OR2AE1,OR2F1,OR2F2,OR6B1,OR6V1,OR9A2,OR9A4,ORAI2,ORC5,OSBPL3,PAPOLB,PARP12,PAX4,PAXIP1,PCLO,PCOLCE,PDAP1,PDE1C,PDGFA,PDIA4,PDK4,PEG10,PEX1,PGAM2,PHF14,PHKG1,PHTF2,PIK3CG,PILRA,PILRB,PIP,PKD1L1,PLEKHA8,PLOD3,PLXNA4,PMPCB,PMS2,PNPLA8,PODXL,POLD2,POLM,POLR2J,POLR2J2,POLR2J3,POM121,POM121C,POM121L12,POMZP3,PON1,PON2,PON3,POP7,POR,POT1,POU6F2,PPIA,PPP1R3A,PPP1R9A,PPP1R17,PPP1R35,PRKAG2,PRKAR1B,PRKAR2B,PRKRIP1,PRPS1L1,PRR15,PRRT4,PRSS1,PRSS37,PRSS58,PSMA2,PSMC2,PSMG3,PSPH,PTCD1,PTN,PTPN12,PTPRN2,PTPRZ1,PURB,PUS7,PVRIG,RAB19,RABGEF1,RAC1,RADIL,RALA,RAMP3,RAPGEF5,RARRES2,RASA4,RASA4B,RBAK,RBAK-RBAKDN,RBM28,RBM33,RBM48,RCC1L,RELN,REPIN1,RFC2,RHBDD2,RHEB,RINT1,RNF32,RNF133,RNF148,RNF216,RP9,RPA3,RSBN1L,RSPH10B,RSPH10B2,RUNDC3B,SAMD9,SAMD9L,SAP25,SBDS,SCIN,SCRN1,SDHAF3,SDK1,SEC61G,SEM1,SEMA3A,SEMA3C,SEMA3D,SEMA3E,SEPT7,SEPT14,SERPINE1,SFRP4,SGCE,SH2B2,SHH,SKAP2,SLC4A2,SLC12A9,SLC13A1,SLC13A4,SLC25A13,SLC25A40,SLC26A3,SLC26A4,SLC26A5,SLC29A4,SLC35B4,SLC37A3,SMARCD3,SMKR1,SMO,SMURF1,SND1,SNX8,SNX10,SNX13,SOSTDC1,SP4,SP8,SPAM1,SPDYE1,SPDYE2,SPDYE2B,SPDYE3,SPDYE5,SPDYE6,SRI,SRPK2,SRRM3,SRRT,SSBP1,SSC4D,SSMEM1,SSPO,ST7,STAG3,STARD3NL,STEAP1,STEAP1B,STEAP2,STEAP4,STK17A,STK31,STRA8,STRIP2,STX1A,STYXL1,SUGCT,SUMF2,SUN1,SUN3,SVOPL,SYPL1,TAC1,TAF6,TARP,TAS2R3,TAS2R4,TAS2R5,TAS2R16,TAS2R38,TAS2R39,TAS2R40,TAS2R41,TAS2R60,TAX1BP1,TBL2,TBRG4,TBX20,TBXAS1,TCAF1,TCAF2,TECPR1,TES,TFEC,TFPI2,TFR2,THAP5,THSD7A,TMED4,TMEM60,TMEM106B,TMEM120A,TMEM130,TMEM139,TMEM140,TMEM168,TMEM176A,TMEM176B,TMEM178B,TMEM184A,TMEM196,TMEM209,TMEM213,TMEM229A,TMEM243,TMEM248,TMUB1,TNPO3,TNRC18,TNS3,TOMM7,TPK1,TPST1,TRA2A,TRIL,TRIM4,TRIM24,TRIM50,TRIM56,TRIM73,TRIM74,TRIP6,TRPV5,TRPV6,TRRAP,TSC22D4,TSGA13,TSPAN12,TSPAN13,TSPAN33,TTC26,TTYH3,TWIST1,TWISTNB,TYW1,TYW1B,UBE2D4,UBE2H,UBE3C,UBN2,UFSP1,UMAD1,UNCX,UPK3B,UPK3BL,UPP1,URGCP,URGCP-MRPS24,USP42,VGF,VIPR2,VKORC1L1,VOPP1,VPS37D,VPS41,VPS50,VSTM2A,VWC2,VWDE,WASL,WBSCR17,WBSCR22,WBSCR27,WBSCR28,WDR60,WDR86,WDR91,WEE2,WIPF3,WIPI2,WNT2,WNT16,XRCC2,YAE1D1,YKT6,YWHAG,ZAN,ZASP,ZBED6CL,ZC3HAV1,ZC3HAV1L,ZC3HC1,ZCWPW1,ZDHHC4,ZFAND2A,ZKSCAN1,ZKSCAN5,ZMIZ2,ZNF3,ZNF12,ZNF92,ZNF107,ZNF117,ZNF138,ZNF212,ZNF273,ZNF277,ZNF282,ZNF316,ZNF394,ZNF398,ZNF425,ZNF467,ZNF479,ZNF655,ZNF679,ZNF680,ZNF713,ZNF716,ZNF727,ZNF735,ZNF736,ZNF746,ZNF775,ZNF777,ZNF783,ZNF786,ZNF789,ZNF800,ZNF804B,ZNF853,ZNF862,ZNHIT1,ZNRF2,ZP3,ZPBP,ZSCAN21,ZSCAN25,ZYX | 885 |  |
| [9:21802748-22008952](genomebrowse://api/zoom?locus=9:21802748-22008952) | 15 | 3 | Het Deletion | | CDKN2A,CDKN2B,MTAP | 3 |
| [15:74363465-74371030](genomebrowse://api/zoom?locus=15:74363465-74371030) | 15 | 2 | Duplicate | GOLGA6A | 1 |  |
| [16:28663278-28784076](genomebrowse://api/zoom?locus=16:28663278-28784076) | 33 | 2 | Duplicate | EIF3C,NPIPB8,NPIPB9 | 3 |  |
| [X:49161883-49361336](genomebrowse://api/zoom?locus=X:49161883-49361336) | 59 | 2 | Duplicate | GAGE2A,GAGE2B,GAGE2C,GAGE2D,GAGE10,GAGE12B,GAGE12C,GAGE12D,GAGE12E,GAGE12F,GAGE12G,GAGE12H,GAGE12I,GAGE12J,GAGE13 | 15 |  |

Lesion

| **Region** | **# Targets** | **# Samples** | **CNV State** | **CNV State** | **Flags** |
| --- | --- | --- | --- | --- | --- |
| [6:31948781-31996054](genomebrowse://api/zoom?locus=6:31948781-31996054) | 66 | 2 | Duplicate | C4A,C4B,STK19 | 3 |
| [16:28663278-28784076](genomebrowse://api/zoom?locus=16:28663278-28784076) | 33 | 2 | Duplicate | EIF3C,NPIPB8,NPIPB9 | 3 |
| **Region** | **# Targets** | **# Samples** | **CNV State** | **CNV State** | **Flags** |
| [1:12952747-13646345](genomebrowse://api/zoom?locus=1:12952747-13646345) | 29 | 1 | Duplicate | HNRNPCL2,PRAMEF5,PRAMEF6,PRAMEF7,PRAMEF8,PRAMEF10,PRAMEF13,PRAMEF15,PRAMEF18,PRAMEF25,PRAMEF33P | 11 |
| [1:45363027-45812451](genomebrowse://api/zoom?locus=1:45363027-45812451) | 77 | 1 | Duplicate | EIF2B3,HECTD3,HPDL,MUTYH,TESK2,TOE1,UROD,ZSWIM5 | 8 |
| [1:147410828-149291038](genomebrowse://api/zoom?locus=1:147410828-149291038) | 53 | 2 | Duplicate | GPR89B,LOC388692,NBPF11,NBPF15,PPIAL4D,PPIAL4E,PPIAL4F,PPIAL4G | 8 |
| [1:152636582-153234364](genomebrowse://api/zoom?locus=1:152636582-153234364) | 29 | 1 | Duplicate | C1orf68,IVL,KPRP,LCE1A,LCE1B,LCE1C,LCE1D,LCE1E,LCE1F,LCE2A,LCE2B,LCE2C,LCE2D,LCE4A,LCE6A,LELP1,LOR,PRR9,SMCP,SPRR1A,SPRR1B,SPRR2A,SPRR2B,SPRR2D,SPRR2E,SPRR2F,SPRR2G,SPRR3,SPRR4 | 29 |
| [2:97879156-98148847](genomebrowse://api/zoom?locus=2:97879156-98148847) | 26 | 1 | Duplicate | ANKRD36,ANKRD36B | 2 |
| [2:107042450-107075788](genomebrowse://api/zoom?locus=2:107042450-107075788) | 18 | 1 | Duplicate | RGPD3 | 1 |
| [2:108454713-108486433](genomebrowse://api/zoom?locus=2:108454713-108486433) | 17 | 1 | Duplicate | RGPD4 | 1 |
| [2:113081729-113191030](genomebrowse://api/zoom?locus=2:113081729-113191030) | 26 | 1 | Duplicate | RGPD8,ZC3H6 | 2 |
| [2:131981169-132121293](genomebrowse://api/zoom?locus=2:131981169-132121293) | 15 | 1 | Duplicate | POTEE,WTH3DI | 2 |
| [2:152449530-152461385](genomebrowse://api/zoom?locus=2:152449530-152461385) | 10 | 1 | Duplicate | NEB | 1 |
| [7:74143124-74173168](genomebrowse://api/zoom?locus=7:74143124-74173168) | 22 | 1 | Duplicate | GTF2I | 1 |
| [7:99906597-99955989](genomebrowse://api/zoom?locus=7:99906597-99955989) | 10 | 1 | Duplicate | PILRB,SPDYE3 | 2 |
| [7:142423216-142494975](genomebrowse://api/zoom?locus=7:142423216-142494975) | 16 | 1 | Duplicate | PRSS1 | 1 |
| [7:143174966-144072768](genomebrowse://api/zoom?locus=7:143174966-144072768) | 45 | 1 | Duplicate | ARHGEF5,ARHGEF35,CTAGE4,CTAGE6,CTAGE8,CTAGE15,OR2A1,OR2A2,OR2A5,OR2A7,OR2A12,OR2A14,OR2A25,OR2A42,OR2F1,OR2F2,OR6B1,TAS2R41,TCAF1,TCAF2 | 20 |
| [7:151851352-151879679](genomebrowse://api/zoom?locus=7:151851352-151879679) | 12 | 1 | Duplicate | KMT2C | 1 |
| [10:46235521-46964019](genomebrowse://api/zoom?locus=10:46235521-46964019) | 38 | 1 | Duplicate | AGAP4,SYT15,WASHC2C | 3 |
| [16:14852804-15044112](genomebrowse://api/zoom?locus=16:14852804-15044112) | 44 | 1 | Duplicate | NOMO1,NPIPA1,NPIPA2 | 3 |
| [16:18511694-18573362](genomebrowse://api/zoom?locus=16:18511694-18573362) | 31 | 3 | Duplicate | NOMO2 | 1 |
| [16:28659456-28834879](genomebrowse://api/zoom?locus=16:28659456-28834879) | 35 | 1 | Duplicate | ATXN2L,EIF3C,NPIPB8,NPIPB9 | 4 |

| **Region** | **# Targets** | **# Samples** | **CNV State** | **Gene Names** | **# Genes** |
| --- | --- | --- | --- | --- | --- |
| [1:12952747-13745175](genomebrowse://api/zoom?locus=1:12952747-13745175) | 41 | 3 | Duplicate | HNRNPCL2,PRAMEF5,PRAMEF6,PRAMEF7,PRAMEF8,PRAMEF10,PRAMEF13,PRAMEF14,PRAMEF15,PRAMEF17,PRAMEF18,PRAMEF19,PRAMEF20,PRAMEF25,PRAMEF33P | 15 |
| [1:145304447-145315899](genomebrowse://api/zoom?locus=1:145304447-145315899) | 10 | 1 | Duplicate | NBPF10 | 1 |
| [1:145748338-146459670](genomebrowse://api/zoom?locus=1:145748338-146459670) | 50 | 1 | Duplicate | GPR89A,NBPF12,PDZK1 | 3 |
| [1:148201967-149291038](genomebrowse://api/zoom?locus=1:148201967-149291038) | 19 | 1 | Duplicate | LOC388692,NBPF15,PPIAL4D,PPIAL4E,PPIAL4F | 5 |
| [1:155580040-155631214](genomebrowse://api/zoom?locus=1:155580040-155631214) | 16 | 4 | Duplicate | MSTO1,YY1AP1 | 2 |
| [1:207696956-207737579](genomebrowse://api/zoom?locus=1:207696956-207737579) | 19 | 1 | Duplicate | CR1 | 1 |
| [2:97875418-97914951](genomebrowse://api/zoom?locus=2:97875418-97914951) | 19 | 3 | Duplicate | ANKRD36 | 1 |
| [2:107042450-107075788](genomebrowse://api/zoom?locus=2:107042450-107075788) | 18 | 1 | Duplicate | RGPD3 | 1 |
| [2:108464797-108486433](genomebrowse://api/zoom?locus=2:108464797-108486433) | 13 | 2 | Duplicate | RGPD4 | 1 |
| [2:131220389-131415450](genomebrowse://api/zoom?locus=2:131220389-131415450) | 42 | 2 | Duplicate | CFC1,CFC1B,POTEI,POTEJ | 4 |
| [2:152437997-152465190](genomebrowse://api/zoom?locus=2:152437997-152465190) | 22 | 1 | Duplicate | NEB | 1 |
| [2:179516393-179528629](genomebrowse://api/zoom?locus=2:179516393-179528629) | 37 | 1 | Duplicate | TTN | 1 |
| [6:30569326-30574020](genomebrowse://api/zoom?locus=6:30569326-30574020) | 12 | 1 | Duplicate | PPP1R10 | 1 |
| [6:31994229-32013103](genomebrowse://api/zoom?locus=6:31994229-32013103) | 47 | 1 | Duplicate | C4B,CYP21A2,TNXB | 3 |
| [7:101961766-102016769](genomebrowse://api/zoom?locus=7:101961766-102016769) | 11 | 1 | Duplicate | LOC100289561,SH2B2,SPDYE6 | 3 |
| [7:143268911-143560889](genomebrowse://api/zoom?locus=7:143268911-143560889) | 18 | 1 | Duplicate | CTAGE6,CTAGE15,TCAF1,TCAF2 | 4 |
| [8:7272483-7835507](genomebrowse://api/zoom?locus=8:7272483-7835507) | 52 | 3 | Duplicate | DEFB4A,DEFB4B,DEFB103A,DEFB103B,DEFB104A,DEFB104B,DEFB105A,DEFB105B,DEFB106A,DEFB106B,DEFB107A,DEFB107B,PRR23D1,PRR23D2,SPAG11A,SPAG11B,USP17L3,USP17L8,ZNF705B | 19 |
| [8:11967409-12293852](genomebrowse://api/zoom?locus=8:11967409-12293852) | 23 | 1 | Duplicate | DEFB130,FAM86B1,FAM86B2,USP17L2,USP17L7,ZNF705D | 6 |
| [15:30375256-30706365](genomebrowse://api/zoom?locus=15:30375256-30706365) | 46 | 2 | Duplicate | CHRFAM7A,GOLGA8J,GOLGA8R | 3 |
| [15:32885755-32912379](genomebrowse://api/zoom?locus=15:32885755-32912379) | 21 | 2 | Duplicate | ARHGAP11A,GOLGA8N | 2 |
| [15:34659188-35045573](genomebrowse://api/zoom?locus=15:34659188-35045573) | 34 | 1 | Duplicate | GJD2,GOLGA8A,GOLGA8B,LPCAT4 | 4 |
| [15:43864967-43892880](genomebrowse://api/zoom?locus=15:43864967-43892880) | 34 | 2 | Duplicate | CKMT1B,PPIP5K1,STRC | 3 |
| [15:72948853-72958683](genomebrowse://api/zoom?locus=15:72948853-72958683) | 17 | 2 | Duplicate | GOLGA6B | 1 |
| [15:75557958-75586816](genomebrowse://api/zoom?locus=15:75557958-75586816) | 27 | 1 | Duplicate | GOLGA6C,GOLGA6D | 2 |
| [16:14852804-15091683](genomebrowse://api/zoom?locus=16:14852804-15091683) | 47 | 1 | Duplicate | NOMO1,NPIPA1,NPIPA2,PDXDC1 | 4 |
| [16:16302585-16485953](genomebrowse://api/zoom?locus=16:16302585-16485953) | 45 | 3 | Duplicate | ABCC6,NOMO3,NPIPA7 | 3 |
| [16:18414874-18569113](genomebrowse://api/zoom?locus=16:18414874-18569113) | 36 | 2 | Duplicate | NOMO2,NPIPA8 | 2 |
| [16:28659456-28784076](genomebrowse://api/zoom?locus=16:28659456-28784076) | 34 | 3 | Duplicate | EIF3C,NPIPB8,NPIPB9 | 3 |
| [16:70161163-70178436](genomebrowse://api/zoom?locus=16:70161163-70178436) | 12 | 3 | Duplicate | PDPR | 1 |
| [16:74442904-74455168](genomebrowse://api/zoom?locus=16:74442904-74455168) | 13 | 3 | Duplicate | CLEC18B | 1 |
| [19:6374822-6387570](genomebrowse://api/zoom?locus=19:6374822-6387570) | 12 | 3 | Duplicate | ALKBH7,GTF2F1,PSPN | 3 |
| [22:16258186-17265299](genomebrowse://api/zoom?locus=22:16258186-17265299) | 13 | 4 | Duplicate | CCT8L2,OR11H1,POTEH,XKR3 | 4 |
| [X:134873998-134967496](genomebrowse://api/zoom?locus=X:134873998-134967496) | 17 | 4 | Duplicate | CT45A3,CT45A5,CT45A8,CT45A9,CT45A10 | 5 |

GBM:6

Blood

| **Region** | **# Targets** | **# Samples** | **CNV State** | **CNV State** | **Flags** |  |
| --- | --- | --- | --- | --- | --- | --- |
| [6:31964921-31994899](genomebrowse://api/zoom?locus=6:31964921-31994899) | 32 | 2 | Duplicate | C4A,C4B | 2 |  |
| [6:32008646-32013103](genomebrowse://api/zoom?locus=6:32008646-32013103) | 14 | 2 | Duplicate | CYP21A2,TNXB | 2 |  |
| [7:38781585-38869928](genomebrowse://api/zoom?locus=7:38781585-38869928) | 21 | 2 | Duplicate | VPS41 | 1 |  |
| [7:54610424-57529655](genomebrowse://api/zoom?locus=7:54610424-57529655) | 135 | 2 | Duplicate | CCT6A,CHCHD2,EGFR,GBAS,LANCL2,MRPS17,NUPR2,PHKG1,PSPH,SEC61G,SEPT14,SUMF2,VOPP1,VSTM2A,ZNF479,ZNF713,ZNF716 | 17 |  |
| [15:72948853-72958226](genomebrowse://api/zoom?locus=15:72948853-72958226) | 15 | 1 | Duplicate | GOLGA6B | 1 |  |
| [16:18530551-18569113](genomebrowse://api/zoom?locus=16:18530551-18569113) | 21 | 1 | Duplicate | NOMO2 | 1 |  |
| [17:6980052-7011876](genomebrowse://api/zoom?locus=17:6980052-7011876) | 10 | 1 | Het Deletion | | ASGR2,CLEC10A | 2 |
| [17:9408355-11645630](genomebrowse://api/zoom?locus=17:9408355-11645630) | 358 | 1 | Duplicate | ADPRM,CFAP52,DHRS7C,DNAH9,GAS7,GLP2R,GSG1L2,MYH1,MYH2,MYH3,MYH4,MYH8,MYH13,PIRT,RCVRN,SCO1,SHISA6,STX8,TMEM220,USP43 | 20 |  |
| [17:18064631-18137722](genomebrowse://api/zoom?locus=17:18064631-18137722) | 21 | 2 | Het Deletion | | ALKBH5,LLGL1,MYO15A | 3 |
| [17:36285779-36347081](genomebrowse://api/zoom?locus=17:36285779-36347081) | 25 | 2 | Duplicate | TBC1D3,TBC1D3L | 2 |  |

| **Region** | **# Targets** | **# Samples** | **CNV State** | **CNV State** | **Flags** |  |
| --- | --- | --- | --- | --- | --- | --- |
| [6:31964921-31994899](genomebrowse://api/zoom?locus=6:31964921-31994899) | 32 | 2 | Duplicate | C4A,C4B | 2 |  |
| [6:32009126-32013103](genomebrowse://api/zoom?locus=6:32009126-32013103) | 13 | 1 | Duplicate | CYP21A2,TNXB | 2 |  |
| [7:54610424-57529655](genomebrowse://api/zoom?locus=7:54610424-57529655) | 135 | 2 | Duplicate | CCT6A,CHCHD2,EGFR,GBAS,LANCL2,MRPS17,NUPR2,PHKG1,PSPH,SEC61G,SEPT14,SUMF2,VOPP1,VSTM2A,ZNF479,ZNF713,ZNF716 | 17 |  |
| [17:8379122-8409807](genomebrowse://api/zoom?locus=17:8379122-8409807) | 17 | 1 | Het Deletion | | MYH10 | 1 |
| [17:18064631-18137722](genomebrowse://api/zoom?locus=17:18064631-18137722) | 21 | 2 | Het Deletion | | ALKBH5,LLGL1,MYO15A | 3 |
| [17:36285779-36347081](genomebrowse://api/zoom?locus=17:36285779-36347081) | 25 | 2 | Duplicate | TBC1D3,TBC1D3L | 2 |  |

| **Region** | **# Targets** | **# Samples** | **CNV State** | **CNV State** | **Flags** |  |
| --- | --- | --- | --- | --- | --- | --- |
| [1:12954417-13745175](genomebrowse://api/zoom?locus=1:12954417-13745175) | 40 | 2 | Duplicate | HNRNPCL2,PRAMEF5,PRAMEF6,PRAMEF7,PRAMEF8,PRAMEF10,PRAMEF13,PRAMEF14,PRAMEF15,PRAMEF17,PRAMEF18,PRAMEF19,PRAMEF20,PRAMEF25,PRAMEF33P | 15 |  |
| [1:145303910-145320619](genomebrowse://api/zoom?locus=1:145303910-145320619) | 17 | 1 | Duplicate | NBPF10 | 1 |  |
| [1:148201967-149761894](genomebrowse://api/zoom?locus=1:148201967-149761894) | 29 | 2 | Duplicate | FAM72C,FAM231D,FCGR1A,HIST2H2BF,LOC388692,NBPF15,PPIAL4C,PPIAL4D,PPIAL4E,PPIAL4F | 10 |  |
| [2:107032306-107075788](genomebrowse://api/zoom?locus=2:107032306-107075788) | 20 | 1 | Duplicate | RGPD3 | 1 |  |
| [2:112552395-112580177](genomebrowse://api/zoom?locus=2:112552395-112580177) | 11 | 1 | Duplicate | ANAPC1 | 1 |  |
| [3:13663275-14163477](genomebrowse://api/zoom?locus=3:13663275-14163477) | 17 | 1 | Het Deletion | | CHCHD4,FBLN2,WNT7A | 3 |
| [4:54319087-56750094](genomebrowse://api/zoom?locus=4:54319087-56750094) | 153 | 1 | Duplicate | CHIC2,CLOCK,EXOC1,FIP1L1,GSX2,KDR,KIT,LNX1,NMU,PDCL2,PDGFRA,SRD5A3,TMEM165 | 13 |  |
| [6:32008646-32013103](genomebrowse://api/zoom?locus=6:32008646-32013103) | 14 | 2 | Duplicate | CYP21A2,TNXB | 2 |  |
| [7:143268911-144072768](genomebrowse://api/zoom?locus=7:143268911-144072768) | 44 | 1 | Duplicate | ARHGEF5,ARHGEF35,CTAGE4,CTAGE6,CTAGE8,CTAGE15,OR2A1,OR2A2,OR2A5,OR2A7,OR2A12,OR2A14,OR2A25,OR2A42,OR2F1,OR2F2,OR6B1,TCAF1,TCAF2 | 19 |  |
| [16:14766464-14957044](genomebrowse://api/zoom?locus=16:14766464-14957044) | 32 | 1 | Duplicate | NOMO1,NPIPA2,NPIPA3,PLA2G10 | 4 |  |
| [16:14988855-15069077](genomebrowse://api/zoom?locus=16:14988855-15069077) | 11 | 1 | Duplicate | NOMO1,NPIPA1,PDXDC1 | 3 |  |
| [16:18414874-18573362](genomebrowse://api/zoom?locus=16:18414874-18573362) | 37 | 2 | Duplicate | NOMO2,NPIPA8 | 2 |  |
| [17:36285779-36455434](genomebrowse://api/zoom?locus=17:36285779-36455434) | 28 | 1 | Duplicate | MRPL45,TBC1D3,TBC1D3L | 3 |  |
| [19:6374476-6387570](genomebrowse://api/zoom?locus=19:6374476-6387570) | 13 | 1 | Duplicate | ALKBH7,GTF2F1,PSPN | 3 |  |
| [19:39263655-39294420](genomebrowse://api/zoom?locus=19:39263655-39294420) | 11 | 1 | Duplicate | LGALS4,LGALS7,LGALS7B | 3 |  |

| **Region** | **# Targets** | **# Samples** | **CNV State** | **CNV State** | **Flags** |
| --- | --- | --- | --- | --- | --- |
| [1:145304447-145316684](genomebrowse://api/zoom?locus=1:145304447-145316684) | 11 | 1 | Duplicate | NBPF10 | 1 |
| [1:152957707-153177480](genomebrowse://api/zoom?locus=1:152957707-153177480) | 10 | 1 | Duplicate | LELP1,SPRR1A,SPRR1B,SPRR2A,SPRR2B,SPRR2D,SPRR2E,SPRR2F,SPRR2G,SPRR3 | 10 |
| [7:101988542-102016769](genomebrowse://api/zoom?locus=7:101988542-102016769) | 10 | 1 | Duplicate | LOC100289561,SPDYE6 | 2 |
| [7:142458406-142494975](genomebrowse://api/zoom?locus=7:142458406-142494975) | 12 | 1 | Duplicate | PRSS1 | 1 |
| [7:143268911-144074283](genomebrowse://api/zoom?locus=7:143268911-144074283) | 45 | 1 | Duplicate | ARHGEF5,ARHGEF35,CTAGE4,CTAGE6,CTAGE8,CTAGE15,OR2A1,OR2A2,OR2A5,OR2A7,OR2A12,OR2A14,OR2A25,OR2A42,OR2F1,OR2F2,OR6B1,TCAF1,TCAF2 | 19 |
| [15:72950899-72958683](genomebrowse://api/zoom?locus=15:72950899-72958683) | 16 | 1 | Duplicate | GOLGA6B | 1 |
| [16:18414874-18573362](genomebrowse://api/zoom?locus=16:18414874-18573362) | 37 | 2 | Duplicate | NOMO2,NPIPA8 | 2 |
