# Supplementary material for: Multiomic profiling of glioblastoma metabolic lesions reveals complex intratumoral genomic evolution and dipeptidase-1-driven vascular proliferation
Source: Neuro Oncol. 2025 May 4;27(10):2547–63. doi: 10.1093/neuonc/noaf071 (PMC12833548; doi:10.1093/neuonc/noaf071)
Supplement: noaf071_Supplementary_Tables_S1-S4_Figures_1-S13 [file noaf071_supplementary_tables_s1-s4_figures_1-s13.zip › Table S3.docx]

SNV All samples

GBM-1

| **Ref/Alt** | **VAF** | **VAF** | **VAF** | **VAF** | **VAF** | **Gene Names** | **Sequence Ontology (Clinically Relevant)** |
| --- | --- | --- | --- | --- | --- | --- | --- |
| C/A | 0 | 0.0343137 | 0.15 | 0 | 0 | ZNF107 | missense_variant |
| C/T | 0 | 0.0341297 | 0.0874862 | 0.00312012 | 0 | MUC12 | missense_variant |
| G/A | 0 | 0.0527704 | 0.104762 | 0 | 0.002326 | PIK3CG | synonymous_variant |
| G/A | 0.00141443 | 0.0344262 | 0.0856202 | 0.00317965 | 0 | MUC17 | missense_variant |
| T/G | 0.0173913 | 0.0394737 | 0.110092 | 0.0603448 | 0.051724 | NR3C2 | synonymous_variant |
| A/T | 0.0178571 | 0.0571429 | 0.057377 | 0.0636364 | 0.048781 | RBMS1 | synonymous_variant |
| -/T | 0.0160428 | 0.0217391 | 0.0504202 | 0.0473373 | 0.025381 | ATG2B | frameshift_variant |
| G/T | 0 | 0.178862 | 0.0178571 | 0.00342466 | 0 | TBC1D2 | missense_variant |
| C/A | 0 | 0.353448 | 0.349693 | 0.0134228 | 0.019737 | HMCN1 | missense_variant |
| C/A | 0 | 0.132948 | 0.0111111 | 0 | 0 | PHC3 | synonymous_variant |
| C/T | 0 | 0.0769231 | 0.106077 | 0.00324675 | 0 | MUC17 | synonymous_variant |
| G/T | 0 | 0.139276 | 0.0216867 | 0 | 0 | FMN1 | intron_variant |
| G/T | 0 | 0.111814 | 0.0137694 | 0 | 0.001862 | SLC12A5 | missense_variant |
| -/CAGCAA | 0 | 0.137681 | 0.106029 | 0.0149626 | 0.010661 | ATN1 | inframe_insertion |
| GCC/- | 0.0168067 | 0.0175439 | 0.0551724 | 0.0504202 | 0.026316 | KLHL11 | inframe_deletion |
| G/C | 0 | 0.40458 | 0.384615 | 0.0522876 | 0.035714 | CAMK1G | missense_variant |
| C/T | 0.0166667 | 0.0431655 | 0.0714286 | 0.0560748 | 0.03937 | MUC12 | synonymous_variant |
| C/T | 0 | 0.515152 | 0.382609 | 0.0442478 | 0.016393 | SEL1L3 | synonymous_variant |
| C/A | 0 | 0.313514 | 0.330275 | 0.0638298 | 0.017778 | OR4C46 | synonymous_variant |
| G/T | 0 | 0.335227 | 0.045082 | 0 | 0 | MARCH4 | missense_variant |
| C/- | 0 | 0.394737 | 0.375 | 0.0516129 | 0.045161 | SLC25A32 | stop_gained |
| A/G | 0 | 0.388535 | 0.382514 | 0.0235294 | 0.046948 | L1TD1 | missense_variant |
| G/A | 0 | 0.470588 | 0.386905 | 0.044586 | 0.013889 | LRP2 | synonymous_variant |
| G/A | 0 | 0.471429 | 0.435644 | 0.0693642 | 0.048309 | SELP | missense_variant |
| A/G | 0 | 0.481752 | 0.371069 | 0.0441176 | 0.042683 | FRRS1L | missense_variant |
| C/T | 0 | 0.142349 | 0.0131579 | 0.00184843 | 0 | PDIA2 | missense_variant |
| G/A | 0 | 0.403756 | 0.357447 | 0.0218341 | 0.048889 | WDR47 | synonymous_variant |
| G/A | 0 | 0.394904 | 0.393443 | 0.0390805 | 0.034884 | TCHH | synonymous_variant |
| C/T | 0 | 0.351474 | 0.313025 | 0.0535332 | 0.045833 | HRNR | missense_variant |
| G/A | 0.00465116 | 0.381443 | 0.389899 | 0.0348259 | 0.044398 | ACKR1 | synonymous_variant |
| C/A | 0 | 0.453552 | 0.437209 | 0.0555556 | 0.027174 | SDCCAG8 | missense_variant |
| G/A | 0.00414079 | 0.430085 | 0.40795 | 0.0636792 | 0.030369 | TPO | synonymous_variant |
| G/A | 0 | 0.353312 | 0.326648 | 0.0520833 | 0.038462 | TDRD15 | missense_variant |
| C/T | 0 | 0.439834 | 0.420849 | 0.0406504 | 0.02963 | HECW2 | synonymous_variant |
| T/C | 0 | 0.349914 | 0.235702 | 0.0488246 | 0.023689 | BARD1 | missense_variant |
| G/A | 0 | 0.440594 | 0.351282 | 0.0472222 | 0.034965 | NYAP2 | synonymous_variant |
| G/A | 0.00238663 | 0.392157 | 0.432927 | 0.0373134 | 0.045268 | KLHL30 | missense_variant |
| C/A | 0 | 0.523404 | 0.403727 | 0.0357143 | 0.058824 | DRD5 | missense_variant |
| G/A | 0 | 0.458182 | 0.258786 | 0.0547445 | 0.049123 | RBM47 | synonymous_variant |
| C/T | 0 | 0.240602 | 0.214286 | 0.0556793 | 0.039139 | SMR3B | synonymous_variant |
| C/T | 0 | 0.494505 | 0.400881 | 0.0210526 | 0.027027 | HPSE | synonymous_variant |
| C/T | 0 | 0.548485 | 0.336134 | 0.0425532 | 0.035714 | TRIML2 | synonymous_variant |
| C/T | 0 | 0.376443 | 0.364444 | 0.0589744 | 0.019139 | TRPC7 | synonymous_variant |
| G/A | 0 | 0.344498 | 0.0224215 | 0.00233645 | 0 | RREB1 | missense_variant |
| C/T | 0 | 0.460606 | 0.425676 | 0.0592593 | 0.006993 | ANKS1A | missense_variant |
| G/A | 0 | 0.440909 | 0.361386 | 0.0454545 | 0.042553 | MEP1A | missense_variant |
| G/A | 0 | 0.43832 | 0.390995 | 0.0314136 | 0.046083 | OPRM1 | missense_variant |
| G/A | 0 | 0.361765 | 0.376263 | 0.0558824 | 0.049223 | ARID1B | synonymous_variant |
| C/T | 0 | 0.26087 | 0.279412 | 0.0327586 | 0.026563 | NCF1 | missense_variant |
| C/T | 0.00436681 | 0.477157 | 0.424242 | 0.0427807 | 0.080189 | MTMR7 | missense_variant |
| C/T | 0.00645161 | 0.426136 | 0.376404 | 0.057554 | 0.033654 | DOCK5 | missense_variant |
| A/C | 0 | 0.333333 | 0.359184 | 0.0445545 | 0.038136 | TMEM67 | missense_variant |
| C/T | 0.00206186 | 0.422747 | 0.362745 | 0.0535332 | 0.030303 | KCNV1 | missense_variant |
| C/T | 0 | 0.869121 | 0.788703 | 0.119048 | 0.08316 | KCNV2 | missense_variant |
| C/T | 0 | 0.45297 | 0.439506 | 0.0453333 | 0.024938 | ABCA1 | missense_variant |
| C/T | 0 | 0.439759 | 0.432961 | 0.0389222 | 0.051282 | TMEM245 | missense_variant |
| C/T | 0 | 0.769231 | 0.7 | 0.0630631 | 0.044444 | ANTXRL | missense_variant |
| A/G | 0 | 0.334038 | 0.0286225 | 0 | 0 | DCHS1 | synonymous_variant |
| C/T | 0 | 0.361371 | 0.308612 | 0.0320856 | 0.043982 | ZNF215 | stop_gained |
| C/T | 0 | 0.496528 | 0.421348 | 0.0678466 | 0.043011 | NLRP10 | synonymous_variant |
| C/T | 0 | 0.433908 | 0.406316 | 0.0501319 | 0.034483 | TMEM132C | synonymous_variant |
| G/A | 0 | 0.391111 | 0.379699 | 0.0549828 | 0.046099 | AKAP11 | missense_variant |
| C/T | 0 | 0.434555 | 0.285088 | 0.0242718 | 0.02 | CCDC168 | synonymous_variant |
| C/T | 0 | 0.801047 | 0.705645 | 0.0340136 | 0.050132 |  | intergenic_variant |
| C/T | 0 | 0.432065 | 0.31401 | 0.0514286 | 0.02122 | CKMT1A | missense_variant |
| G/T | 0 | 0.302326 | 0.242038 | 0.0501139 | 0.029979 | PIF1 | missense_variant |
| G/A | 0 | 0.41704 | 0.314286 | 0.0384615 | 0.035398 | THSD4 | missense_variant |
| C/T | 0 | 0.421053 | 0.381395 | 0.0582011 | 0.044226 | NR2F2 | missense_variant |
| G/A | 0 | 0.436261 | 0.397849 | 0.0442478 | 0.050667 | CACNG3 | missense_variant |
| C/G | 0 | 0.467033 | 0.404624 | 0.0584416 | 0.012821 | CDH1 | missense_variant |
| A/T | 0 | 0.414013 | 0.414918 | 0.0358127 | 0.02934 | ZNF469 | missense_variant |
| G/C | 0 | 0.429054 | 0.4375 | 0.0253623 | 0.056886 | ACADVL | synonymous_variant |
| C/G | 0.00215054 | 0.867102 | 0.803922 | 0.109865 | 0.08243 | TP53 | missense_variant |
| G/A | 0 | 0.919708 | 0.827815 | 0.121495 | 0.079096 | GID4 | synonymous_variant |
| C/T | 0 | 0.901316 | 0.851695 | 0.133531 | 0.106667 | TMEM94 | missense_variant |
| T/G | 0 | 0.350254 | 0.373418 | 0.0452128 | 0.052381 | ALPK2 | synonymous_variant |
| C/T | 0 | 0.449438 | 0.332326 | 0.0361446 | 0.057895 | ELANE | synonymous_variant |
| C/T | 0 | 0.466334 | 0.480818 | 0.0418848 | 0.035417 | ATP13A1 | missense_variant |
| G/T | 0 | 0.408602 | 0.291469 | 0.0507042 | 0.040094 | KIAA0355 | missense_variant |
| G/A | 0 | 0.437318 | 0.472892 | 0.0481586 | 0.028721 | CEACAM6 | missense_variant |
| CCGCCTGAACCG/- | 0 | 0.33642 | 0.334311 | 0.0397878 | 0.010152 | CPT1C | inframe_deletion |
| C/T | 0 | 0.288503 | 0.0119048 | 0.0021322 | 0 | ZNF473 | synonymous_variant |
| G/A | 0 | 0.392473 | 0.290323 | 0.048 | 0.040665 | ZSCAN5B | synonymous_variant |
| C/A | 0 | 0.147541 | 0.0132013 | 0 | 0 | COL18A1 | stop_gained |
| G/A | 0 | 0 | 0.165049 | 0 | 0 | TCN1 | synonymous_variant |
| G/T | 0.00414938 | 0 | 0.175325 | 0 | 0 | CCNL2 | missense_variant |
| T/G | 0 | 0 | 0.126712 | 0 | 0 | ARAP3 | synonymous_variant |
| T/G | 0 | 0 | 0 | 0.13355 | 0 | APOL1 | synonymous_variant |
| C/A | 0 | 0 | 0.0964912 | 0 | 0 | CYP4A22 | missense_variant |
| G/A | 0 | 0.00213675 | 0.00468384 | 0.0874036 | 0.082774 | ATP8B3 | missense_variant |
| C/T | 0.00438596 | 0 | 0.319219 | 0.0138122 | 0 | LNX1 | synonymous_variant |
| C/T | 0 | 0 | 0.238191 | 0.0197802 | 0 | FIP1L1 | missense_variant |
| G/A | 0 | 0.00138504 | 0.486879 | 0.0101351 | 0 | PDGFRA | missense_variant |
| T/A | 0 | 0 | 0.495318 | 0.0153257 | 0 | PDGFRA | missense_variant |
| T/C | 0 | 0 | 0.127753 | 0.0030349 | 0 | TSPAN10 | missense_variant |
| A/C | 0 | 0 | 0.0530973 | 0.021645 | 0 | CASR | missense_variant |

GBM-2

| **Ref/Alt** | **VAF** | **VAF** | **VAF** | **VAF** | **Gene Names** | **Sequence Ontology (Clinically Relevant)** |
| --- | --- | --- | --- | --- | --- | --- |
| C/G | 0.015625 | 0.00746269 | 0.0327869 | 0.964072 | ALG1L2 | synonymous_variant |
| GAG/- | 0.0192308 | 0.0677966 | 0.0526316 | 0.0431034 | MEX3A | inframe_deletion |
| TGTGCTTA/- | 0 | 0.258333 | 0.229167 | 0.0684932 | CWC25 | frameshift_variant |
| T/G | 0.00212766 | 0.0765661 | 0 | 0 | GCAT | missense_variant |
| C/G | 0.0078125 | 0.204819 | 0.23125 | 0.0666667 | SLC15A4 | synonymous_variant |
| G/A | 0 | 0.286957 | 0.209091 | 0.0619469 | MAZ | synonymous_variant |
| C/T | 0 | 0.317757 | 0.168317 | 0.0806452 | GPSM2 | missense_variant |
| C/T | 0 | 0.229299 | 0.210843 | 0.0877193 | TRPA1 | missense_variant |
| G/A | 0 | 0.356436 | 0.132743 | 0.0964912 | CCDC173 | missense_variant |
| C/T | 0 | 0.267606 | 0.179641 | 0.0818713 | AK7 | synonymous_variant |
| A/G | 0 | 0.28777 | 0.223529 | 0.0621118 | DNAI2 | missense_variant |
| C/T | 0 | 0.283784 | 0.191388 | 0.0653266 | MYH8 | missense_variant |
| C/A | 0 | 0.312057 | 0.185629 | 0.0534351 | IFFO2 | missense_variant |
| C/T | 0 | 0.367521 | 0.275 | 0.111765 | ITIH2 | synonymous_variant |
| C/T | 0.00217865 | 0.115663 | 0.0643863 | 0.0169133 | SLC35G4 | missense_variant |
| C/T | 0 | 0.259668 | 0.154696 | 0.0673077 | MOCS2 | missense_variant |
| C/T | 0 | 0.270115 | 0.202703 | 0.0588235 | OR4N4 | missense_variant |
| C/T | 0 | 0.18797 | 0.00401606 | 0 | ZNF727 | synonymous_variant |
| G/A | 0 | 0.276243 | 0.174312 | 0.0430622 | TRPM6 | missense_variant |
| G/A | 0 | 0.331126 | 0.20603 | 0.0878049 | BRD8 | synonymous_variant |
| C/T | 0 | 0.288889 | 0.176471 | 0.120219 | PRR23C | missense_variant |
| G/A | 0 | 0.233766 | 0.254717 | 0.0696721 | CLCA2 | missense_variant |
| C/T | 0 | 0.19244 | 0.16092 | 0.1 | FOSB | missense_variant |
| G/T | 0 | 0.247788 | 0.213115 | 0.0776256 | TRAPPC11 | missense_variant |
| C/G | 0 | 0.323529 | 0.155 | 0.137143 | CIAO1 | missense_variant |
| T/C | 0 | 0.266055 | 0.177358 | 0.0730769 | TMPRSS11F | synonymous_variant |
| G/T | 0 | 0.213287 | 0.166667 | 0.040146 | OR7A17 | synonymous_variant |
| C/T | 0 | 0.186391 | 0.0108696 | 0.00273224 | ADGRB1 | synonymous_variant |
| C/T | 0 | 0.221429 | 0.128358 | 0.0537975 | CRB2 | synonymous_variant |
| C/T | 0 | 0.386667 | 0.194969 | 0.060241 | CTSE | missense_variant |
| G/A | 0 | 0.227758 | 0.210345 | 0.0695364 | GORASP1 | synonymous_variant |
| G/T | 0 | 0.279476 | 0.161049 | 0.0745098 | SPATA8 | missense_variant |
| T/C | 0 | 0.56 | 0.336842 | 0.0989011 | CXorf66 | missense_variant |
| C/G | 0 | 0.232639 | 0.138728 | 0.0766962 |  | intergenic_variant |
| C/T | 0 | 0.271605 | 0.199301 | 0.0263158 | MYO1B | synonymous_variant |
| C/T | 0 | 0.309524 | 0.220833 | 0.0794224 | GPR61 | synonymous_variant |
| C/T | 0 | 0.21021 | 0.146893 | 0.0301205 | AKAP8 | missense_variant |
| T/C | 0 | 0.273438 | 0.178988 | 0.0413223 | IFI16 | synonymous_variant |
| G/A | 0 | 0.285156 | 0.168498 | 0.0873016 | PTTG1IP | 3_prime_UTR_variant |
| A/T | 0 | 0.313305 | 0.153527 | 0.0569395 | LAMA1 | synonymous_variant |
| C/T | 0 | 0.246951 | 0.00955794 | 0.00151976 | DLGAP3 | missense_variant |
| C/T | 0 | 0.202273 | 0.012024 | 0 | OR2T10 | missense_variant |
| T/C | 0 | 0.301887 | 0.186567 | 0.0466321 | POTEE | missense_variant |
| G/A | 0 | 0.320175 | 0.219048 | 0.0539326 | ERBB4 | missense_variant |
| G/A | 0 | 0.301961 | 0.195122 | 0.0808081 | SCN10A | missense_variant |
| C/T | 0 | 0.341146 | 0.205128 | 0.0637119 | MFSD10 | synonymous_variant |
| -/A | 0 | 0.307692 | 0.191693 | 0.0553846 | VCAN | frameshift_variant |
| C/T | 0.00185874 | 0.270522 | 0.193103 | 0.0654762 | PCDHB5 | synonymous_variant |
| G/A | 0 | 0.928525 | 0.939645 | 0.839343 | EGFR | missense_variant |
| G/C | 0 | 0.217886 | 0.165021 | 0.0637523 | SVOPL | synonymous_variant |
| G/A | 0.00235294 | 0.123776 | 0.0936937 | 0.0411111 |  | intergenic_variant |
| G/A | 0 | 0.214092 | 0.162602 | 0.0465116 | CNTNAP2 | synonymous_variant |
| C/T | 0 | 0.232975 | 0.158385 | 0.0663812 | AGAP3 | missense_variant |
| G/A | 0 | 0.247664 | 0.12931 | 0.0689655 | VIPR2 | missense_variant |
| C/T | 0 | 0.294737 | 0.172414 | 0.0880952 | ZER1 | synonymous_variant |
| G/A | 0.00208768 | 0.340426 | 0.193309 | 0.0659794 | NELFB | missense_variant |
| C/T | 0 | 0.433735 | 0.280591 | 0.092437 | TACC2 | synonymous_variant |
| G/A | 0 | 0.196517 | 0.0585774 | 0.00247525 | PHRF1 | synonymous_variant |
| C/A | 0 | 0.303502 | 0.186508 | 0.0722022 | RIC3 | stop_gained |
| C/T | 0 | 0.26076 | 0.196653 | 0.0772443 | SHANK2 | missense_variant |
| G/A | 0 | 0.420561 | 0.330097 | 0.0963303 | ESPL1 | missense_variant |
| G/T | 0 | 0.455882 | 0.239583 | 0.137755 | KSR2 | missense_variant |
| C/G | 0 | 0.463768 | 0.258706 | 0.140704 | KSR2 | missense_variant |
| C/T | 0 | 0.272116 | 0.217514 | 0.0723606 | SEZ6L2 | missense_variant |
| A/C | 0 | 0.285 | 0.209567 | 0.0588235 | FAM83G,SLC5A10 | missense_variant,intron_variant |
| ACAG/- | 0 | 0.267123 | 0.195035 | 0.0902527 | DDX5 | splice_donor_variant |
| G/A | 0 | 0.231908 | 0.197767 | 0.092827 | ATP8B3 | synonymous_variant |
| C/T | 0 | 0.218997 | 0.196262 | 0.0897833 | SLC7A10 | missense_variant |
| G/A | 0 | 0.284182 | 0.227041 | 0.0623229 | MEGF8 | synonymous_variant |
| G/A | 0 | 0.27931 | 0.209964 | 0.0664452 | CLTCL1 | synonymous_variant |
| T/A | 0 | 0.285024 | 0.185941 | 0.0762332 | RASD2 | missense_variant |
| A/C | 0 | 0 | 0.144681 | 0 | A2ML1 | missense_variant |
| G/T | 0 | 0 | 0.0441176 | 0.0811456 | OR9Q1 | synonymous_variant |
| G/A | 0 | 0 | 0.0531401 | 0 | CLIP2 | missense_variant |

GBM-3

| **Ref/Alt** | **VAF** | **VAF** | **VAF** | **VAF** | **VAF** | **Gene** | **Sequence Ontology (Clinically Relevant)** |
| --- | --- | --- | --- | --- | --- | --- | --- |
| G/- | 0 | 0.0127389 | 0.0746269 | 0.344371 | 0.0472973 | MEX3A | frameshift_variant |
| A/- | 0.00704225 | 0.00492611 | 0.0387097 | 0.282209 | 0.00649351 | CCDC38 | frameshift_variant |
| TGC/- | 0.01 | 0.0512821 | 0.0380952 | 0.0336134 | 0.0619469 | R3HDM2 | inframe_deletion |
| GGC/- | 0.00869565 | 0.0314136 | 0.0484848 | 0.0545455 | 0.0204082 | BHLHE22 | inframe_deletion |
| C/T | 0 | 0 | 0.0555556 | 0.257143 | 0.0276243 | AGBL1 | missense_variant |
| G/T | 0 | 0 | 0.106557 | 0.378571 | 0.030303 | RYR2 | missense_variant |
| G/A | 0 | 0.00540541 | 0.0849673 | 0.398964 | 0.0454545 | ADGRL2 | missense_variant |
| C/A | 0 | 0 | 0.152439 | 0.502513 | 0.0632911 | CAPN13 | missense_variant |
| T/A | 0 | 0 | 0.0576923 | 0.289855 | 0.031746 | REXO1 | missense_variant |
| A/C | 0 | 0 | 0.0932643 | 0.245059 | 0.0408163 | KDM7A | missense_variant |
| A/G | 0 | 0 | 0.108571 | 0.391534 | 0.0616114 | CATSPER3 | missense_variant |
| G/A | 0 | 0 | 0.0520833 | 0.208494 | 0.0324074 | SLC26A3 | missense_variant |
| A/C | 0 | 0 | 0 | 0 | 0.195266 | RSRC2 | missense_variant |
| G/A | 0 | 0 | 0.117021 | 0.383621 | 0.0226244 | CCER1 | missense_variant |
| G/A | 0 | 0 | 0.0689655 | 0.366492 | 0.0649351 | NOP56 | missense_variant |
| T/G | 0 | 0 | 0.201183 | 0 | 0 | OTULIN | missense_variant |
| C/T | 0 | 0 | 0.0531915 | 0.281081 | 0.0232558 | TP53INP1 | missense_variant |
| C/T | 0 | 0 | 0.11828 | 0.263736 | 0.0608696 | SLC12A1 | missense_variant |
| C/T | 0 | 0.00350877 | 0.097166 | 0.238342 | 0.0474308 | EPHB4 | missense_variant |
| C/T | 0 | 0 | 0.0847458 | 0.363946 | 0.0260223 | SGSM1 | missense_variant |
| G/A | 0 | 0 | 0.0912698 | 0.39172 | 0.0352113 | OR5R1 | missense_variant |
| C/T | 0 | 0.0026455 | 0.089404 | 0.354396 | 0.0430108 | FCN1 | missense_variant |
| C/T | 0 | 0.0026178 | 0.160819 | 0.357843 | 0.0324484 | TMC6 | missense_variant |
| T/A | 0 | 0.00260417 | 0.0643087 | 0.226087 | 0.0225989 | ZNF479 | missense_variant |
| G/A | 0 | 0 | 0.108787 | 0.274691 | 0.0428135 | RPE | missense_variant |
| G/A | 0 | 0 | 0.103858 | 0.386574 | 0.0431655 | TRPC7 | missense_variant |
| C/T | 0 | 0.00243902 | 0.0987654 | 0.21881 | 0.0156658 | ZNF135 | missense_variant |
| G/A | 0 | 0 | 0.078125 | 0.387755 | 0.0204604 | OR2W5 | missense_variant |
| C/T | 0.00288184 | 0 | 0.116208 | 0.627737 | 0.0473538 | TP53 | missense_variant |
| G/A | 0 | 0 | 0.0909091 | 0.386207 | 0.035533 | MOV10L1 | missense_variant |
| C/T | 0.00292398 | 0.0020202 | 0.0809249 | 0.348837 | 0.0375 | FLT4 | missense_variant |
| G/A | 0.00257732 | 0 | 0.101877 | 0.391061 | 0.035124 | CRELD1 | missense_variant |
| C/T | 0.00239234 | 0 | 0.0782998 | 0.295615 | 0.0307167 | ZNF71 | missense_variant |
| T/G | 0 | 0 | 0.0791506 | 0 | 0.00136986 | LOC101928841 | missense_variant |
| C/T | 0 | 0 | 0.0535714 | 0.263441 | 0.0534351 | IL1RL1 | stop_gained |
| C/T | 0 | 0 | 0.114583 | 0.347826 | 0.0758929 | FAM98C | stop_gained |
| -/TAGTCCAC | 0 | 0 | 0.0972222 | 0.44863 | 0.0325203 | CHRND | stop_gained |
| C/A | 0 | 0.00297619 | 0.042735 | 0.236364 | 0.027668 | CNTNAP3B | stop_gained |
| C/T | 0 | 0 | 0.097561 | 0.668605 | 0.0471014 | PTEN | stop_gained |
| C/T | 0 | 0 | 0.0717822 | 0.34386 | 0.0372549 | TNK2 | stop_gained |
| G/A | 0 | 0 | 0.057623 | 0.256007 | 0.0360082 | FLG | stop_gained |
| G/A | 0 | 0 | 0.0921053 | 0.284768 | 0.0261438 | NDUFAF6 | synonymous_variant |
| C/T | 0 | 0 | 0.0877193 | 0.347059 | 0.0588235 | ZNF319 | synonymous_variant |
| G/A | 0 | 0 | 0.0909091 | 0.449438 | 0.0224719 | MROH2B | synonymous_variant |
| T/C | 0 | 0 | 0.0643777 | 0.314103 | 0.0355556 | GNAT3 | synonymous_variant |
| A/G | 0 | 0 | 0.078341 | 0.325671 | 0.0412844 | SERPINA11 | synonymous_variant |
| G/A | 0 | 0 | 0.0996169 | 0.385057 | 0.0627063 | CACNA1G | synonymous_variant |
| G/A | 0 | 0.00296736 | 0.1 | 0.325301 | 0.0539419 | UBR1 | synonymous_variant |
| C/T | 0 | 0 | 0.0900621 | 0.394015 | 0.0690691 | TOR4A | synonymous_variant |
| C/T | 0.00448431 | 0 | 0.108696 | 0.397306 | 0.0606061 | SLC13A2 | synonymous_variant |
| C/T | 0 | 0 | 0.0699301 | 0.397394 | 0.0470588 | UGT2B7 | synonymous_variant |
| G/A | 0 | 0 | 0.0231214 | 0.248918 | 0.027933 | PPP1R13L | synonymous_variant |
| A/G | 0 | 0 | 0.102941 | 0.355882 | 0.027027 | LRRC4C | synonymous_variant |
| C/T | 0 | 0.0020202 | 0.103175 | 0.383562 | 0.0573951 | SHARPIN | synonymous_variant |
| G/A | 0 | 0 | 0.116959 | 0.4 | 0.0196507 | RTN1 | synonymous_variant |
| G/A | 0.00271003 | 0 | 0.106024 | 0.363636 | 0.0514403 | BCAR1 | synonymous_variant |
| C/T | 0.003003 | 0 | 0.0920716 | 0.376147 | 0.056338 | OR56A1 | synonymous_variant |
| C/T | 0 | 0 | 0.100251 | 0.282178 | 0.0492505 | MUC16 | synonymous_variant |
| G/A | 0 | 0 | 0.0994152 | 0.282167 | 0.0459459 | NT5C1B,NT5C1B-RDH14 | synonymous_variant,synonymous_variant |
| G/A | 0 | 0.00245098 | 0.107527 | 0.373832 | 0.011236 | MTUS2 | x |

GBM-4

| **Ref/Alt** | **VAF** | **VAF** | **VAF** | **VAF** | **VAF** | **Gene Names** | **Sequence Ontology (Clinically Relevant)** |
| --- | --- | --- | --- | --- | --- | --- | --- |
| C/T | 0.00297619 | 0.526316 | 0.0153846 | 0.00872093 | 0.338753 | OPRK1 | 3_prime_UTR_variant |
| T/- | 0 | 0.323232 | 0 | 0 | 0.276995 | IGSF21 | frameshift_variant |
| T/G | 0 | 0.00930233 | 0.164251 | 0 | 0 | OR10A2 | initiator_codon_variant |
| G/T | 0 | 0.276543 | 0.00486618 | 0 | 0.0595238 | HYAL3,NAT6 | intron_variant,missense_variant |
| G/A | 0.00189394 | 0.316372 | 0.0142857 | 0.00497512 | 0.336502 | EYS,LOC441155 | intron_variant,missense_variant |
| C/G | 0.00952381 | 0.075 | 0.0510204 | 0.0719424 | 0.0588235 | LGR4 | missense_variant |
| C/T | 0 | 0.0537634 | 0.0108401 | 0.00697674 | 0.287206 | CYP1A2 | missense_variant |
| A/G | 0 | 0.101887 | 0.00828729 | 0 | 0.303896 | NEO1 | missense_variant |
| C/T | 0 | 0.274194 | 0 | 0 | 0.302013 | CPNE5 | missense_variant |
| C/T | 0 | 0.308943 | 0.00714286 | 0 | 0.174194 | IGFN1 | missense_variant |
| A/C | 0.0137615 | 0.0508475 | 0.08 | 0.0394737 | 0.0375 | C10orf142 | missense_variant |
| C/T | 0 | 0.323741 | 0.00952381 | 0 | 0.288889 | PTGER3 | missense_variant |
| T/G | 0 | 0.163009 | 0 | 0 | 0 | ALMS1 | missense_variant |
| T/C | 0 | 0.282178 | 0 | 0.00571429 | 0.0348837 | TBL2 | missense_variant |
| T/G | 0 | 0.18429 | 0 | 0 | 0 | ALMS1 | missense_variant |
| C/T | 0 | 0.27907 | 0.0248447 | 0.00512821 | 0.204348 | ASZ1 | missense_variant |
| G/T | 0 | 0.403846 | 0.0202703 | 0 | 0.402299 | MPHOSPH10 | missense_variant |
| G/A | 0.00558659 | 0.379888 | 0.0166667 | 0 | 0.401198 | MAPK7 | missense_variant |
| A/C | 0 | 0.255973 | 0 | 0 | 0.219048 | CFTR | missense_variant |
| T/C | 0 | 0.357843 | 0.0223881 | 0.005 | 0.358824 | PUM1 | missense_variant |
| G/A | 0.00657895 | 0.387097 | 0.011976 | 0.00621118 | 0.29375 | CD1E | missense_variant |
| C/T | 0 | 0.36859 | 0.020979 | 0.00546448 | 0.35942 | ZNF436 | missense_variant |
| G/A | 0 | 0.397321 | 0.0402299 | 0.0045045 | 0.323529 | DDR2 | missense_variant |
| T/C | 0 | 0.343675 | 0.00561798 | 0 | 0.269058 | TP53BP2 | missense_variant |
| G/C | 0 | 0.357447 | 0 | 0.00220264 | 0.352298 | PCYT1A | missense_variant |
| A/G | 0 | 0.366667 | 0.0181818 | 0 | 0.316 | SLIT2 | missense_variant |
| C/T | 0 | 0.368254 | 0.0144404 | 0 | 0.338109 | OTUD4 | missense_variant |
| C/T | 0 | 0.351724 | 0.017316 | 0 | 0.31085 | BDP1 | missense_variant |
| C/T | 0 | 0.39313 | 0.0121457 | 0 | 0.351974 | ADGRV1 | missense_variant |
| C/T | 0.0019305 | 0.320298 | 0.0133588 | 0.00660066 | 0.265781 | PCDHB7 | missense_variant |
| C/G | 0 | 0.377892 | 0.00611621 | 0.00224719 | 0.30163 | GPR151 | missense_variant |
| C/T | 0 | 0.363636 | 0.0174419 | 0 | 0.338501 | COL23A1 | missense_variant |
| C/A | 0 | 0.34 | 0.00425532 | 0.00373134 | 0.350195 | GNL1 | missense_variant |
| C/T | 0 | 0.369681 | 0.0084507 | 0.00478469 | 0.326146 | COL11A2 | missense_variant |
| C/A | 0 | 0.276786 | 0.0045045 | 0.0035461 | 0.240331 | AHR | missense_variant |
| G/A | 0 | 0.241584 | 0.0166205 | 0.00692841 | 0.253333 | DDC | missense_variant |
| C/T | 0 | 0.088729 | 0.00500357 | 0.00068871 | 0.0932282 | MUC12 | missense_variant |
| A/T | 0 | 0.27051 | 0.0171821 | 0.00268817 | 0.344186 | NAT1 | missense_variant |
| G/C | 0 | 0.491682 | 0.0169014 | 0.00468384 | 0.379562 | TEX15 | missense_variant |
| C/T | 0 | 0.517442 | 0.0130435 | 0 | 0.121324 | COL14A1 | missense_variant |
| C/T | 0 | 0.378906 | 0.020202 | 0 | 0.298805 | RANBP6 | missense_variant |
| C/T | 0 | 0.332258 | 0.011583 | 0 | 0.263699 | FOLR3 | missense_variant |
| A/C | 0 | 0.384354 | 0 | 0 | 0.35443 | RILPL2 | missense_variant |
| T/G | 0 | 0.535714 | 0.0269058 | 0.0128755 | 0.34322 | BTBD1 | missense_variant |
| C/G | 0 | 0.344482 | 0.0202492 | 0.00412088 | 0.320863 | ZNF469 | missense_variant |
| G/A | 0 | 0.650376 | 0.00246305 | 0 | 0.440111 | TP53 | missense_variant |
| G/A | 0.004329 | 0.6 | 0 | 0.00378788 | 0.379888 | DNAH2 | missense_variant |
| A/T | 0 | 0.269444 | 0.0208333 | 0 | 0.298482 | TEX2 | missense_variant |
| C/T | 0 | 0.339695 | 0.00934579 | 0.00495049 | 0.320138 | FAM71E2 | missense_variant |
| G/T | 0 | 0.363014 | 0 | 0.00576369 | 0.327703 | MMP24 | missense_variant |
| T/C | 0 | 0.34965 | 0 | 0 | 0.257143 | CYP24A1 | missense_variant |
| G/A | 0 | 0.385686 | 0.019084 | 0.010101 | 0.301766 | TRPM2 | missense_variant |
| T/G | 0 | 0 | 0.100686 | 0.0125673 | 0 | KCNJ4 | missense_variant |
| T/G | 0 | 0 | 0.0542986 | 0 | 0.00113636 | REN | missense_variant |
| C/T | 0 | 0.369403 | 0 | 0 | 0.0497925 | LIMS1 | stop_gained |
| C/A | 0.00207039 | 0.386 | 0.0211416 | 0.0035461 | 0.332696 | ANKRD18A | stop_gained |
| G/A | 0.00657895 | 0.0576923 | 0.0106383 | 0.0327103 | 0.0233918 | GOLGA6A | synonymous_variant |
| C/T | 0 | 0.272277 | 0.0223464 | 0.0150376 | 0.285068 | ZDHHC1 | synonymous_variant |
| A/G | 0 | 0.34104 | 0.0121212 | 0.0102041 | 0.285714 | SPTA1 | synonymous_variant |
| C/A | 0 | 0.375433 | 0.00865052 | 0.0058997 | 0.316527 | HSPG2 | synonymous_variant |
| C/T | 0 | 0.369099 | 0 | 0 | 0.327586 | HMCN1 | synonymous_variant |
| C/G | 0 | 0.42437 | 0.0103093 | 0.00406504 | 0.335 | UTRN | synonymous_variant |
| C/T | 0 | 0.403448 | 0.0151515 | 0.00307692 | 0.355263 | SYNE1 | synonymous_variant |
| T/C | 0.0021692 | 0.239757 | 0.0207039 | 0.002 | 0.248711 | GTF2IRD2 | synonymous_variant |
| G/A | 0 | 0.351351 | 0 | 0 | 0.0595238 | TMEM199 | synonymous_variant |
| C/T | 0 | 0.356275 | 0 | 0 | 0.294393 | ABCC3 | synonymous_variant |
| A/G | 0 | 0.385757 | 0.00453172 | 0.00282885 | 0.25523 | FUT1 | synonymous_variant |
| C/T | 0 | 0.339552 | 0.0309735 | 0.00729927 | 0.317829 | GTPBP1 | synonymous_variant |
| T/A | 0 | 0.754717 | 0.0340136 | 0.0167598 | 0.724138 | ATP11C | synonymous_variant |
| C/T | 0 | 0 | 0.0016835 | 0 | 0.059593 | PRAMEF2 | synonymous_variant |

GBM-5

| **Ref/Alt** | **VAF** | **VAF** | **VAF** | **VAF** | **VAF** | **Gene Names** | **Sequence Ontology (Clinically Relevant)** |
| --- | --- | --- | --- | --- | --- | --- | --- |
| AT/- | 0 | 0.296296 | 0.25 | 0.268908 | 0 | NF1 | frameshift_variant |
| T/- | 0 | 0.472779 | 0.420074 | 0.432602 | 0.128814 | NECTIN3 | frameshift_variant |
| C/- | 0 | 0.267857 | 0.168627 | 0.213115 | 0.069853 | MECOM | frameshift_variant |
| TG/- | 0 | 0.001919 | 0 | 0 | 0.076923 | NF1 | frameshift_variant |
| CGG/- | 0 | 0.246667 | 0.230769 | 0.315789 | 0.056338 | SOX5 | inframe_deletion |
| C/T | 0 | 0.305085 | 0.233251 | 0.262032 | 0.075758 |  | intergenic_variant |
| C/T | 0.003125 | 0.321138 | 0.297297 | 0.315315 | 0 | KIF1B | intron_variant |
| A/C | 0.014218 | 0.082279 | 0.05 | 0.048485 | 0.069307 | RUNX1 | missense_variant |
| C/T | 0 | 0.212903 | 0.196262 | 0.216667 | 0.066667 | ATR | missense_variant |
| G/A | 0.002793 | 0.151786 | 0.030303 | 0.032609 | 0.005435 | KRT83 | missense_variant |
| G/A | 0 | 0.280576 | 0.27957 | 0.378378 | 0.062992 | C1orf167 | missense_variant |
| A/G | 0 | 0.236686 | 0.25 | 0.19697 | 0 | SULT1C3 | missense_variant |
| T/G | 0 | 0.248485 | 0.258621 | 0.253521 | 0.08 | TRAPPC11 | missense_variant |
| T/A | 0 | 0.153061 | 0.173333 | 0.172297 | 0.030303 | ZNF479 | missense_variant |
| G/T | 0 | 0.193966 | 0.230769 | 0.273585 | 0.042169 | TTN | missense_variant |
| G/A | 0.003322 | 0.177358 | 0.24 | 0.178862 | 0 | ITPR1 | missense_variant |
| G/A | 0 | 0.289308 | 0.287037 | 0.23741 | 0.057971 | SPACA1 | missense_variant |
| T/C | 0 | 0.216814 | 0.212121 | 0.216561 | 0 | SLC9A4 | missense_variant |
| G/A | 0 | 0.253731 | 0.215116 | 0.303922 | 0.061539 | OR7A5 | missense_variant |
| C/T | 0 | 0.3125 | 0.277311 | 0.316547 | 0.04698 | DNAH14 | missense_variant |
| C/T | 0 | 0.237288 | 0.233129 | 0.276995 | 0 | GRID2 | missense_variant |
| C/A | 0 | 0.292929 | 0.305195 | 0.231579 | 0.046632 | UTP20 | missense_variant |
| C/T | 0 | 0.273543 | 0.290698 | 0.248705 | 0 | GRIN2B | missense_variant |
| C/T | 0 | 0.290476 | 0.25 | 0.236364 | 0.09 | UNC13C | missense_variant |
| G/A | 0.014634 | 0.034965 | 0.027523 | 0.062016 | 0.051282 | TPSAB1 | missense_variant |
| C/T | 0 | 0.276423 | 0.259459 | 0.319635 | 0.098039 | P2RY10 | missense_variant |
| G/A | 0 | 0.248201 | 0.25 | 0.292776 | 0.003922 | APLN | missense_variant |
| C/T | 0 | 0.323944 | 0.263804 | 0.314815 | 0.064286 | ACAP3 | missense_variant |
| G/T | 0 | 0.280303 | 0.274775 | 0.257143 | 0.060241 | SAMD4B | missense_variant |
| C/A | 0 | 0.330317 | 0.241379 | 0.323529 | 0 | C2CD3 | missense_variant |
| C/A | 0 | 0.294821 | 0.287554 | 0.288066 | 0 | KIF4A | missense_variant |
| C/T | 0.002 | 0.21968 | 0.175978 | 0.230769 | 0.003268 | SCN5A | missense_variant |
| C/T | 0 | 0.197826 | 0.157609 | 0.219512 | 0.069182 | SCN11A | missense_variant |
| G/A | 0 | 0.284182 | 0.248148 | 0.251592 | 0.049689 | NWD2 | missense_variant |
| G/A | 0 | 0.308197 | 0.336 | 0.339934 | 0.041667 | FREM3 | missense_variant |
| C/T | 0 | 0.28839 | 0.269058 | 0.339623 | 0.06087 | MFAP3L | missense_variant |
| C/A | 0 | 0.357388 | 0.272 | 0.277966 | 0 | TENM3 | missense_variant |
| T/A | 0 | 0.294964 | 0.240741 | 0.235521 | 0 | FAT2 | missense_variant |
| A/G | 0 | 0.337302 | 0.352941 | 0.281553 | 0.08 | OR2H1 | missense_variant |
| G/A | 0 | 0.20751 | 0.143223 | 0.178879 | 0 | TNRC18 | missense_variant |
| T/C | 0 | 0.317647 | 0.270588 | 0.348315 | 0.046693 | PPP1R3B | missense_variant |
| T/A | 0 | 0.390476 | 0.273256 | 0.2891 | 0.06 | EXOSC4 | missense_variant |
| C/T | 0 | 0.284639 | 0.271523 | 0.312057 | 0.06367 | MUC5B | missense_variant |
| G/C | 0.002976 | 0.332016 | 0.268817 | 0.276243 | 0 | SLC2A14 | missense_variant |
| C/T | 0 | 0.316265 | 0.264407 | 0.263323 | 0 | ZNF469 | missense_variant |
| A/G | 0 | 0.294964 | 0.247934 | 0.245763 | 0.008368 | ABR | missense_variant |
| G/A | 0 | 0.29085 | 0.259912 | 0.277966 | 0.046595 | MYH13 | missense_variant |
| C/T | 0 | 0.306905 | 0.299363 | 0.289552 | 0.087879 | PLIN3 | missense_variant |
| G/A | 0.002882 | 0.339056 | 0.262857 | 0.277228 | 0.069565 | USP29 | missense_variant |
| G/T | 0 | 0.279365 | 0.256579 | 0.28125 | 0 | CECR1 | missense_variant |
| A/C | 0 | 0.310861 | 0.296117 | 0.345622 | 0.074236 | FANCB | missense_variant |
| C/A | 0 | 0.341346 | 0.262295 | 0.279141 | 0 | CDK16 | missense_variant |
| G/A | 0 | 0.286996 | 0.240896 | 0.249389 | 0.081461 | CACNA1F | missense_variant |
| C/T | 0.001724 | 0.307143 | 0.28125 | 0.304688 | 0 | FLNA | missense_variant |
| C/T | 0 | 0.272727 | 0.242424 | 0.364407 | 0 | PATJ | stop_gained |
| G/A | 0 | 0.3125 | 0.206186 | 0.281818 | 0 | NF1 | stop_gained |
| C/T | 0 | 0.258929 | 0.238994 | 0.303191 | 0.087963 | VN1R2 | stop_gained |
| C/T | 0 | 0.314433 | 0.231343 | 0.232044 | 0.051814 | GZMK | stop_gained |
| G/T | 0 | 0.321608 | 0.271386 | 0.290909 | 0.065903 | MAP7D2 | stop_gained |
| G/T | 0 | 0.316716 | 0.288136 | 0.242902 | 0.094637 | SLITRK4 | stop_gained |
| A/C | 0.005208 | 0.00216 | 0.09375 | 0 | 0 | PIGO | stop_gained |
| C/T | 0 | 0.274336 | 0.228261 | 0.396396 | 0.073171 | SLCO1B1 | synonymous_variant |
| G/A | 0 | 0.135338 | 0 | 0 | 0 | OR5B3 | synonymous_variant |
| C/T | 0 | 0.136842 | 0.156627 | 0.136691 | 0.039326 | TRRAP | synonymous_variant |
| G/A | 0 | 0.306011 | 0.274194 | 0.369159 | 0 | MROH2B | synonymous_variant |
| C/T | 0 | 0.314721 | 0.225131 | 0.255435 | 0.053571 | SCNN1D | synonymous_variant |
| C/T | 0 | 0.280769 | 0.218605 | 0.277778 | 0 | THEMIS | synonymous_variant |
| G/A | 0 | 0.272727 | 0.220264 | 0.257937 | 0 | FLNA | synonymous_variant |
| C/T | 0.001323 | 0.330922 | 0.238938 | 0.282332 | 0 | FLG | synonymous_variant |
| G/A | 0 | 0.276139 | 0.345455 | 0.320588 | 0.003546 | SPRR1A | synonymous_variant |
| G/A | 0.000459 | 0.052983 | 0.040635 | 0.048942 | 0.015212 | MUC12 | synonymous_variant |
| T/A | 0 | 0.16329 | 0.165605 | 0.180305 | 0.05988 | MUC17 | synonymous_variant |
| A/G | 0 | 0.260753 | 0.274678 | 0.255738 | 0 | FAM186A | synonymous_variant |
| T/C | 0 | 0.286885 | 0.272152 | 0.325879 | 0 | TNK1 | synonymous_variant |
| C/T | 0 | 0.271008 | 0.253197 | 0.275488 | 0 | BCOR | synonymous_variant |
| C/T | 0 | 0.259136 | 0.243902 | 0.308696 | 0.084211 | NONO | synonymous_variant |

GBM-6

| **Ref/Alt** | **VAF** | **VAF** | **VAF** | **VAF** | **VAF** | **Gene Names** | **Sequence Ontology (Clinically Relevant)** |
| --- | --- | --- | --- | --- | --- | --- | --- |
| TAGCCTACAG/- | 0 | 0.448718 | 0.387324 | 0.0833333 | 0 | GADL1 | frameshift_variant |
| GTCCCGCCCCGGCCCCCACCCCCGCCT/- | 0 | 0.283333 | 0.215054 | 0.0483871 | 0 | RUSC1 | inframe_deletion |
| AGG/- | 0 | 0.448819 | 0.392593 | 0.0670391 | 0.00925926 | ZNF107 | inframe_deletion |
| T/C | 0 | 0.548673 | 0.488372 | 0.0887097 | 0.00869565 | ZNF287 | initiator_codon_variant |
| C/T | 0 | 0.16849 | 0.12963 | 0.0060423 | 0.00321543 |  | intergenic_variant |
| C/A | 0 | 0.166481 | 0.155975 | 0.048583 | 0.00096062 |  | intergenic_variant |
| G/C | 0.0116959 | 0.037037 | 0.0451977 | 0.0218579 | 0.0545455 | ABCA9 | missense_variant |
| G/A | 0 | 0.172619 | 0.219512 | 0.0299401 | 0.0190476 | PGLYRP3 | missense_variant |
| C/T | 0 | 0.0278207 | 0.00179533 | 0.0949868 | 0 | NMU | missense_variant |
| G/A | 0 | 0.161157 | 0.191919 | 0.0607477 | 0 | KCTD16 | missense_variant |
| A/C | 0 | 0.280576 | 0 | 0 | 0 | EMC7 | missense_variant |
| G/A | 0 | 0.184426 | 0.220513 | 0.0267857 | 0.0168539 | CCDC27 | missense_variant |
| T/G | 0 | 0.0846561 | 0 | 0 | 0 | CRLF1 | missense_variant |
| C/T | 0 | 0.277108 | 0.268456 | 0.075 | 0.0147059 | ANKRD18A | missense_variant |
| G/C | 0.017341 | 0.0483871 | 0.0588235 | 0.0428571 | 0.0560748 | TTK | missense_variant |
| G/A | 0.00609756 | 0.0461538 | 0.0185185 | 0.0592593 | 0.0545455 | MUC4 | missense_variant |
| C/T | 0 | 0.5 | 0.505376 | 0.0426829 | 0 | TP53 | missense_variant |
| T/C | 0 | 0.405797 | 0.363636 | 0.0531915 | 0.00595238 | DRD2 | missense_variant |
| C/T | 0.00440529 | 0.299492 | 0.307692 | 0.046875 | 0 | PTPRR | missense_variant |
| G/A | 0.00234742 | 0.558282 | 0.510345 | 0.0982659 | 0.0111111 | SLIT2 | missense_variant |
| C/T | 0 | 0.734783 | 0.668317 | 0.124161 | 0.0111732 | PLA2G7 | missense_variant |
| G/A | 0 | 0.248826 | 0.218274 | 0.09699 | 0 | VIPR2 | missense_variant |
| G/T | 0 | 0.304729 | 0.276392 | 0.0791667 | 0.00584795 | SPATA31A6 | missense_variant |
| C/T | 0 | 0.333333 | 0.270992 | 0.0795848 | 0 | PAPPA | missense_variant |
| G/A | 0.00371747 | 0.296178 | 0.283217 | 0.048 | 0 | OBP2B | missense_variant |
| C/T | 0 | 0.596491 | 0.55102 | 0.164835 | 0.008 | IQSEC3 | missense_variant |
| C/T | 0 | 0.377709 | 0.259136 | 0.0366972 | 0 | SLCO1B7 | missense_variant |
| C/G | 0 | 0.697959 | 0.650224 | 0.14094 | 0.0105263 | RPGRIP1 | missense_variant |
| C/T | 0 | 0.488806 | 0.414097 | 0.0765957 | 0.0157068 | CACNA1H | missense_variant |
| A/T | 0 | 0.69265 | 0.629121 | 0.219858 | 0.0478723 | MYH1 | missense_variant |
| G/A | 0 | 0.684713 | 0.657993 | 0.085044 | 0.00338983 | LOXHD1 | missense_variant |
| G/A | 0.00547945 | 0.289389 | 0.258993 | 0.0648855 | 0.00446429 | MUC16 | missense_variant |
| C/T | 0.00321543 | 0.766102 | 0.646226 | 0.119691 | 0.0208333 | TEX13C | missense_variant |
| A/G | 0 | 0.746888 | 0.698113 | 0.160142 | 0.0209424 | BRCC3 | missense_variant |
| T/G | 0 | 0 | 0.0609137 | 0 | 0 | ADGRG1 | missense_variant |
| C/G | 0 | 0.983425 | 0.00571429 | 0.0223881 | 0 | PLEKHA2 | splice_acceptor_variant |
| C/T | 0 | 0.175 | 0.153226 | 0.0228758 | 0.0101523 | OR10K1 | stop_gained |
| G/A | 0 | 0.259459 | 0.214286 | 0.122222 | 0 | CYP27C1 | stop_gained |
| G/T | 0 | 0.334884 | 0.225275 | 0.0414938 | 0 | RSRC2 | stop_gained |
| -/ACCCT | 0 | 0.397436 | 0.349138 | 0.100503 | 0.00632911 | BAZ2B | stop_gained |
| G/A | 0.0147059 | 0.0764331 | 0.0451613 | 0.108108 | 0.0526316 | CNTNAP3 | synonymous_variant |
| C/T | 0 | 0.0258949 | 0.00116077 | 0.0876289 | 0 | NMU | synonymous_variant |
| G/A | 0 | 0.169014 | 0.154589 | 0.036036 | 0.00584795 | DNAH5 | synonymous_variant |
| C/T | 0 | 0.224719 | 0.152866 | 0.0287356 | 0 | WDR6 | synonymous_variant |
| G/A | 0 | 0.0555556 | 0.141509 | 0.00847458 | 0 | ANGPTL5 | synonymous_variant |
| A/G | 0 | 0.301471 | 0.262295 | 0.0970149 | 0 | PARP11 | synonymous_variant |
| C/T | 0 | 0.0723781 | 0.0977918 | 0 | 0 | ADH4 | synonymous_variant |
| G/A | 0.00387597 | 0.24186 | 0.232044 | 0.036 | 0.00552486 | ATL3 | synonymous_variant |
| A/T | 0 | 0.369863 | 0.359712 | 0.048951 | 0 | POTEM | synonymous_variant |
| G/A | 0.00200401 | 0.193939 | 0.159722 | 0.0298507 | 0.00837989 | CRP | synonymous_variant |
| C/T | 0.0017094 | 0.450581 | 0.405405 | 0.100167 | 0.0136054 | STEAP3 | synonymous_variant |
| G/A | 0 | 0.32732 | 0.281768 | 0.0553936 | 0.00434783 | TTN | synonymous_variant |
| G/A | 0 | 0.551562 | 0.484 | 0.0857664 | 0.00239234 | NBEAL2 | synonymous_variant |
| A/G | 0 | 0.51462 | 0.505747 | 0.0777202 | 0.0145985 | ANKRD17 | synonymous_variant |
| G/A | 0 | 0.52657 | 0.489247 | 0.112745 | 0.00598802 | C7 | synonymous_variant |
| C/G | 0 | 0.419922 | 0.425721 | 0.0880196 | 0 | ANAPC2 | synonymous_variant |
| C/T | 0.00443787 | 0.216758 | 0.206422 | 0.0294118 | 0.00229358 | MUC5B | synonymous_variant |
| C/T | 0 | 0.433333 | 0.384615 | 0.0466102 | 0 | FOXO1 | synonymous_variant |
| C/T | 0.00469484 | 0.438871 | 0.394161 | 0.0573066 | 0 | FMN1 | synonymous_variant |
| G/A | 0.00323625 | 0.284483 | 0.28012 | 0.0684524 | 0.00448431 | FBN3 | synonymous_variant |
| G/A | 0 | 0.395498 | 0.454545 | 0.0434783 | 0.00487805 | ZNF134 | synonymous_variant |
| C/T | 0.00217391 | 0.246193 | 0.237785 | 0.0426065 | 0.00946372 | TGM2 | synonymous_variant |
