# Supplementary material for: Multiomic profiling of glioblastoma metabolic lesions reveals complex intratumoral genomic evolution and dipeptidase-1-driven vascular proliferation
Source: Neuro Oncol. 2025 May 4;27(10):2547–63. doi: 10.1093/neuonc/noaf071 (PMC12833548; doi:10.1093/neuonc/noaf071)
Supplement: noaf071_Supplementary_Tables_S1-S4_Figures_1-S13 [file noaf071_supplementary_tables_s1-s4_figures_1-s13.zip › Table S4.docx]

Fusion gene all Samples

| **FusionName** | **LeftBreakpoint** | **RightBreakpoint** | **sample** |
| --- | --- | --- | --- |
| ABHD12--KANK1(4479),DMRT1(91105) | 20:25323325 | 9:750585 | C3 |
| AC092691.1--LSAMP | 3:117997182 | 3:116812396 | B9 |
| AL049544.1--KHDRBS2 | 6:61606290 | 6:61697253 | B4 |
| AL049544.1--KHDRBS2 | 6:61606290 | 6:61697253 | C1 |
| AL049544.1--KHDRBS2 | 6:61606290 | 6:61697253 | D9 |
| AL391840.3--SH3BGRL2 | chr6:79538843:+ | chr6:79673614:+ | A2 |
| AL391840.3--SH3BGRL2 | chr6:79538843:+ | chr6:79673614:+ | A3 |
| AL391840.3--SH3BGRL2 | chr6:79538843:+ | chr6:79673614:+ | A8 |
| AL391840.3--SH3BGRL2 | chr6:79538843:+ | chr6:79673614:+ | A9 |
| AL391840.3--SH3BGRL2 | chr6:79538843:+ | chr6:79673614:+ | B1 |
| AL391840.3--SH3BGRL2 | chr6:79538843:+ | chr6:79673614:+ | B4 |
| AL391840.3--SH3BGRL2 | chr6:79538843:+ | chr6:79673614:+ | B8 |
| AL391840.3--SH3BGRL2 | chr6:79538843:+ | chr6:79673614:+ | B9 |
| AL391840.3--SH3BGRL2 | chr6:79538843:+ | chr6:79673614:+ | C1 |
| AL391840.3--SH3BGRL2 | chr6:79538843:+ | chr6:79673614:+ | C3 |
| AL391840.3--SH3BGRL2 | chr6:79538843:+ | chr6:79673614:+ | C8 |
| AL391840.3--SH3BGRL2 | chr6:79538843:+ | chr6:79673614:+ | C9 |
| AL391840.3--SH3BGRL2 | chr6:79538843:+ | chr6:79673614:+ | D1 |
| AL391840.3--SH3BGRL2 | chr6:79538843:+ | chr6:79673614:+ | D3 |
| AL391840.3--SH3BGRL2 | chr6:79538843:+ | chr6:79673614:+ | D4 |
| AL391840.3--SH3BGRL2 | chr6:79538843:+ | chr6:79673614:+ | D8 |
| AL391840.3--SH3BGRL2 | chr6:79538843:+ | chr6:79673614:+ | D9 |
| AL391840.3--SH3BGRL2 | chr6:79538843:+ | chr6:79673614:+ | E4 |
| ARHGAP31--ZNF385D | chr3:119368516:+ | chr3:21665028:- | C3 |
| ARHGAP31--ZNF385D | chr3:119368516 | chr3:21665028 | D3 |
| ATP2C1--NMNAT3 | chr3:130894775:+ | chr3:139579055:- | C3 |
| ATP2C1--NMNAT3 | chr3:130894775:+ | chr3:139579055:- | D3 |
| CACNA2D1--GLI3 | chr7:82170550 | chr7:41872008 | A3 |
| CACNA2D1--INHBA-AS1(92620),GLI3(88942) | 7:82170550 | 7:41872008 | C3 |
| CADPS--ASTE1 | 3:62645722 | 3:131025594 | C3 |
| CADPS--ASTE1 | 3:62645722 | 3:131025594 | C3 |
| CAVIN4--MSANTD3-TMEFF1 | 9:100585927 | 9:100498765 | B4 |
| CAVIN4--MSANTD3-TMEFF1 | 9:100585927 | 9:100498765 | C1 |
| CAVIN4--MSANTD3-TMEFF1 | 9:100585927 | 9:100498765 | D1 |
| CAVIN4--MSANTD3-TMEFF1 | 9:100585927 | 9:100498765 | D3 |
| CAVIN4--MSANTD3-TMEFF1 | 9:100585927 | 9:100498765 | D8 |
| CAVIN4--MSANTD3-TMEFF1 | 9:100585927 | 9:100498765 | D9 |
| CAVIN4--MSANTD3-TMEFF1 | 9:100585927 | 9:100498765 | D9 |
| CAVIN4--MSANTD3-TMEFF1 | 9:100585927 | 9:100498765 | E4 |
| CAVIN4--TMEFF1 | 9:100585927 | 9:100498765 | A3 |
| CAVIN4--TMEFF1 | 9:100585927 | 9:100498765 | B4 |
| CAVIN4--TMEFF1 | 9:100585927 | 9:100498765 | C1 |
| CAVIN4--TMEFF1 | 9:100585927 | 9:100498765 | D1 |
| CAVIN4--TMEFF1 | 9:100585927 | 9:100498765 | D3 |
| CAVIN4--TMEFF1 | 9:100585927 | 9:100498765 | D8 |
| CAVIN4--TMEFF1 | 9:100585927 | 9:100498765 | D9 |
| CAVIN4--TMEFF1 | 9:100585927 | 9:100498765 | E4 |
| CDH12P3(63905),AC138866.2(377)--GUSBP3 | 5:70196878 | 5:69642289 | A2 |
| CDH12P3(63905),AC138866.2(377)--GUSBP3 | 5:70196878 | 5:69642289 | B1 |
| CDH12P3(63905),AC138866.2(377)--GUSBP3 | 5:70196878 | 5:69642289 | D4 |
| CDH12P3(63905),AC138866.2(377)--GUSBP3 | 5:70196878 | 5:69642289 | E4 |
| CDK14--AC092681.1 | 7:90604249 | 7:149909705 | A1 |
| CDK14--AC092681.1 | 7:90604249 | 7:149909705 | B1 |
| CHIC2--AC105384.1 | chr4:54048955:- | chr4:53899920:+ | A9 |
| CHIC2--AC105384.1 | chr4:54048955:- | chr4:53899920:+ | A9 |
| CHIC2--AC105384.1 | chr4:54048955:- | chr4:53899920:+ | A9 |
| CHIC2--AC105384.1 | chr4:54048955:- | chr4:53899920:+ | A9 |
| CHIC2--SCFD2 | chr4:54064182:- | chr4:52874061:- | D1 |
| CHIC2--SCFD2 | chr4:54064182:- | chr4:52874061:- | D1 |
| CHIC2--SCFD2 | chr4:54064182:- | chr4:52874061:- | D1 |
| CMSS1--AC114477.1(88782),RANP7(3306) | 3:99818043 | 3:22900829 | C3 |
| CMSS1--AC114477.1(88782),RANP7(3306) | 3:99818043 | 3:22900829 | C3 |
| CXADR--AP000959.1(149527),AP000949.1(212997) | 21:17547193 | 21:22669585 | A8 |
| CXADR--AP000959.1(149527),AP000949.1(212997) | 21:17547193 | 21:22669585 | A9 |
| FNDC3B--KCNAB1 | chr3:172362845 | chr3:156239504 | B3 |
| FNDC3B--KCNAB1 | 3:172362845 | 3:156239504 | C3 |
| HERC1--CA12 | 15:63749367 | 15:63346709 | A1 |
| HERC1--CA12 | 15:63749367 | 15:63346709 | B1 |
| HERC1--CA12 | 15:63749367 | 15:63346709 | C1 |
| KHDRBS2-OT--KHDRBS2 | chr6:61652255:- | chr6:61697253:- | B3 |
| KHDRBS2-OT--KHDRBS2 | chr6:61652255:- | chr6:61697253:- | B4 |
| KHDRBS2-OT--KHDRBS2 | chr6:61652255:- | chr6:61697253:- | C1 |
| KHDRBS2-OT--KHDRBS2 | chr6:61652255:- | chr6:61697253:- | D3 |
| KHDRBS2-OT--KHDRBS2 | chr6:61652255:- | chr6:61697253:- | D4 |
| KHDRBS2-OT--KHDRBS2 | chr6:61652255:- | chr6:61697253:- | D8 |
| KHDRBS2-OT--KHDRBS2 | chr6:61652255:- | chr6:61697253:- | D9 |
| LANCL2--AC015908.7(151715),AC015908.2(151715),RN7SL601P(124845) | 7:55412089 | 17:11032610 | A8 |
| LANCL2--AC015908.7(151715),AC015908.2(151715),RN7SL601P(124845) | 7:55412089 | 17:11032610 | A9 |
| LANCL2--AC015908.7(151715),AC015908.2(151715),RN7SL601P(124845) | 7:55412089 | 17:11032610 | C9 |
| LCLAT1--KCNH7 | 2:30533314 | 2:162537080 | A8 |
| LCLAT1--KCNH7 | 2:30533314 | 2:162537080 | A9 |
| LRP1--AC090022.1(475972),AC090017.1(335894) | 12:57187456 | 12:60895265 | A4 |
| LRP1--AC090022.1(475972),AC090017.1(335894) | 12:57187456 | 12:60895265 | E4 |
| LRRC42--GLIS1 | 1:53960474 | 1:53600278 | C3 |
| LSAMP--IGF2BP2 | chr3:116444877:- | chr3:185698347:- | C3 |
| LSAMP--IGF2BP2 | chr3:116444877:- | chr3:185698347:- | C3 |
| LSAMP--IGF2BP2 | chr3:116444877:- | chr3:185698347:- | C3 |
| LSAMP--IGF2BP2 | chr3:116444877:- | chr3:185698347:- | C3 |
| MCCC1--AC034187.1 | chr3:183086693:- | chr3:8573874:- | C3 |
| MED12L--SENP7(4323),FAM172BP(4327) | 3:151127984 | 3:101517564 | C3 |
| MED12L--SENP7(4323),FAM172BP(4327) | 3:151127984 | 3:101517564 | C3 |
| MIR4300HG--PRCP | chr11:82713069:- | chr11:82860117:- | A8 |
| MIR4300HG--PRCP | chr11:82713069:- | chr11:82860117:- | A9 |
| MIR4300HG--PRCP | chr11:82713069:- | chr11:82860117:- | C9 |
| MITF--LMLN | 3:69739701 | 3:198031056 | C3 |
| MITF--RAD54L2 | 3:69739701 | 3:51657580 | C3 |
| MSL3(102265),RF00019(176806)--FRMPD4 | X:11878018 | X:12498680 | C1 |
| MSL3(102265),RF00019(176806)--FRMPD4 | X:11878018 | X:12498680 | D1 |
| MSL3(102265),RF00019(176806)--FRMPD4 | X:11878018 | X:12498680 | D9 |
| MTAP--CDKN2AB-AS1 | chr9:21818202 | chr9:22112320 | A1 |
| MTAP--CDKN2AB-AS1 | chr9:21818202 | chr9:22112320 | C1 |
| MTAP--CDKN2B-AS1 | 9:21818202 | 9:22112320 | B1 |
| MTAP--CDKN2B-AS1 | 9:21818202 | 9:22112320 | D1 |
| MTMR2--CEP57 | chr11:95923875:- | chr11:95812932:+ | B8 |
| MTMR2--CEP57 | chr11:95923875:- | chr11:95812932:+ | B9 |
| MTMR2--CEP57 | chr11:95923875:- | chr11:95812932:+ | C8 |
| MTMR2--CEP57 | chr11:95923875:- | chr11:95812932:+ | D8 |
| MTMR2--CEP57 | chr11:95923875 | chr11:95812932 | D9 |
| MTRNR2L9(31888),AL049544.1(23943)--KHDRBS2 | 6:61606290 | 6:61697253 | C1 |
| MTRNR2L9(31888),AL049544.1(23943)--KHDRBS2 | 6:61606290 | 6:61697253 | D1 |
| MTRNR2L9(31888),AL049544.1(23943)--KHDRBS2 | 6:61606290 | 6:61697253 | D4 |
| MTRNR2L9(31888),AL049544.1(23943)--KHDRBS2 | 6:61606290 | 6:61697253 | D8 |
| MTRNR2L9(31888),AL049544.1(23943)--KHDRBS2 | 6:61606290 | 6:61697253 | D9 |
| NBAS--AC010745.2 | chr2:15511212:- | chr2:16228024:+ | A8 |
| NBAS--AC010745.2 | chr2:15511212:- | chr2:16239389:+ | A8 |
| NBAS--FAM84A(128333),AC068286.1(107500) | 2:15218773 | 2:14779147 | A8 |
| NBAS--FAM84A(128333),AC068286.1(107500) | 2:15218773 | 2:14779147 | A9 |
| NBAS--FAM84A(128333),AC068286.1(107500) | 2:15218773 | 2:14779147 | A9 |
| NBAS--FAM84A(62481),AC068286.1(173352) | 2:15218773 | 2:14713295 | A8 |
| NBAS--FAM84A(62481),AC068286.1(173352) | 2:15218773 | 2:14713295 | A9 |
| NBAS--FAM84A(62481),AC068286.1(173352) | 2:15218773 | 2:14713295 | A9 |
| NCAM2--LINC01425(33972),AP000472.1(101928) | 21:20998618 | 21:21831387 | A9 |
| NEK10--NR2C2 | 3:27141482 | 3:15003876 | C3 |
| NGLY1--TMCC1 | 3:25778574 | 3:129671264 | C3 |
| NGLY1--TMCC1 | 3:25778574 | 3:129671264 | C3 |
| NGLY1--TMCC1 | 3:25778574 | 3:129671264 | C3 |
| NLGN1--CLDN18(13055),DZIP1L(15280) | 3:173807832 | 3:138046710 | C3 |
| NSUN6--AC096725.1(377595),LINC02494(28850) | 10:18642476 | 4:58495665 | A1 |
| OSBPL11--AC078918.1(422196),AC117430.1(127519) | 3:125567394 | 3:110399963 | C3 |
| PBRM1--KCNAB1 | 3:52651742 | 3:156375433 | C3 |
| PDCD6IP--LAPTM4BP2(2350),GXYLT2(1044) | 3:33845588 | 3:72887029 | C3 |
| PDGFRA--FIP1L1 | chr4:54229415:+ | chr4:53425872:+ | A1 |
| PDGFRA--FIP1L1 | chr4:54229415:+ | chr4:53425872:+ | B1 |
| PDGFRA--TMEM165 | chr4:54290554:+ | chr4:55452239:+ | A8 |
| PLXNA1--GADL1(139239),MIR466(127700) | 3:127022818 | 3:31034004 | C3 |
| PLXNA1--GADL1(162226),MIR466(104713) | 3:127022818 | 3:31056991 | C3 |
| PPP2R2A--AC073581.1(33052),DOCK5(9703) | 8:26293740 | 8:25175020 | A4 |
| PPP2R2A--AC073581.1(33052),DOCK5(9703) | 8:26293740 | 8:25175020 | A4 |
| PPP2R2A--AC073581.1(33052),DOCK5(9703) | 8:26293740 | 8:25175020 | E4 |
| PPP4R1--ANKRD12 | 18:9549197 | 18:9254211 | A2 |
| PRBM1--KCNAB1 | chr3:52651742 | chr3:156375433 | D3 |
| PTN--DPY19L2P3 | chr7:137343439:- | chr7:29731948:+ | B8 |
| PTN--DPY19L2P3 | chr7:137343439:- | chr7:29731948:+ | B9 |
| PTN--DPY19L2P3 | chr7:137343439:- | chr7:29731948:+ | C8 |
| PTPRZ1--LINC01510(39956),MET(17614) | 7:121928221 | 7:116654776 | A1 |
| PTPRZ1--LINC01510(39956),MET(17614) | 7:121928221 | 7:116654776 | B1 |
| RAD54L2--AC104629.1(171793),LINC01326(103547) | 3:51656170 | 3:166466146 | C3 |
| RAD54L2--AC104629.1(171793),LINC01326(103547) | 3:51656170 | 3:166466146 | C3 |
| RBMS3--CADM2 | 3:29899755 | 3:85471304 | C3 |
| RBMS3--CADM2 | 3:29899755 | 3:85471304 | C3 |
| RPSAP53(30527),LINC00364(74154)--PCDH9 | 13:67298233 | 13:66631411 | B4 |
| RPSAP53(30527),LINC00364(74154)--PCDH9 | 13:67298233 | 13:66631411 | D4 |
| RSRC1--AC108740.1(78241),AC068756.1(240496) | 3:158123991 | 3:80520546 | C3 |
| RSRC1--AC108740.1(78241),AC068756.1(240496) | 3:158123991 | 3:80520546 | C3 |
| RSRC1--AC108740.1(78241),AC068756.1(240496) | 3:158123991 | 3:80520546 | C3 |
| RYK--BBX | 3:134195082 | 3:107710452 | C3 |
| RYK--BBX | chr3:134195082 | chr3:107710452 | D3 |
| SENP7--CADPS | chr3:101493873:- | chr3:62592748:- | C3 |
| SETD2--FRMD4B | 3:47116623 | 3:69250099 | B3 |
| SETD2--FRMD4B | 3:47116623 | 3:69250099 | C3 |
| STX8--THSD7A | chr17:9505038:- | chr7:11636961:- | A8 |
| STX8--THSD7A | chr17:9505038:- | chr7:11636961:- | A9 |
| STX8--THSD7A | chr17:9505038:- | chr7:11636961:- | C9 |
| TFEC--CAV1 | chr7:116110708:- | chr7:116526525:+ | A1 |
| TMEM165--PDGFRA | chr4:55396396:+ | chr4:54267289:+ | A1 |
| TMEM165--PDGFRA | chr4:55396396:+ | chr4:54267289:+ | B1 |
| UBXN7--STIMATE | 3:196361844 | 3:52855444 | C3 |
| UBXN7--TMEM110-MUSTN1 | 3:196361844 | 3:52855444 | C3 |
| VGLL4--DOCK3 | chr3:11643437 | chr3:51360511 | B3 |
| VGLL4--DOCK3 | chr3:11643437 | chr3:51374469 | B3 |
| VGLL4--DOCK3 | chr3:11643437 | chr3:51374487 | B3 |
| VGLL4--DOCK3 | chr3:11643437 | chr3:51374469 | D3 |
| VGLL4--DOCK3 | chr3:11643437 | chr3:51374487 | D3 |
| VGLL4--DOCK3_1 | 3:11643437 | 3:51374487 | C3 |
| VGLL4--DOCK3_2 | 3:11643437 | 3:51374469 | C3 |
| VGLL4--DOCK3_3 | 3:11643437 | 3:51360511 | C3 |
| WDR35-PIGF | 2:19975530 | 2:46600594 | A8 |
| WDR35-PIGF | 2:19975530 | 2:46600594 | A9 |
| ZBTB20-EPHA3_1 | 3:114500352 | 3:89395837 | C3 |
| ZBTB20-EPHA3_2 | 3:114454579 | 3:89395837 | C3 |
| ZMAT3--EPHB1 | 3:179067483 | 3:134689261 | C3 |
| ZNF208--PAF1 | 19:21985605 | 19:39390289 | B2 |
