# Supplementary figures and images for: Multiomic profiling of glioblastoma metabolic lesions reveals complex intratumoral genomic evolution and dipeptidase-1-driven vascular proliferation
Source: Neuro Oncol. 2025 May 4;27(10):2547–63. doi: 10.1093/neuonc/noaf071 (PMC12833548; doi:10.1093/neuonc/noaf071)

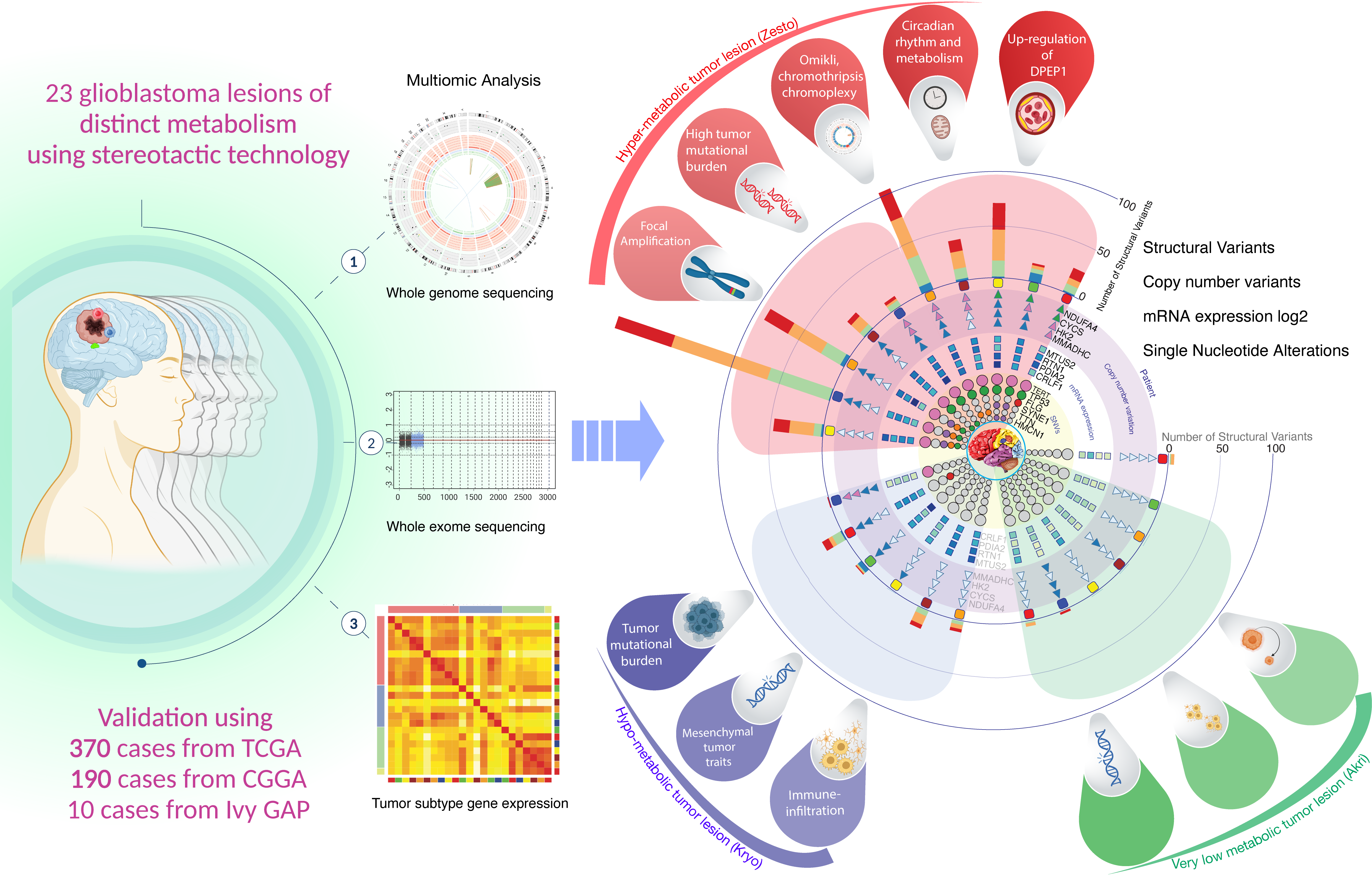

Supplement: noaf071_Supplementary_Tables_S1-S4_Figures_1-S13 [file noaf071_supplementary_tables_s1-s4_figures_1-s13.zip › Graphical abstract.tif]

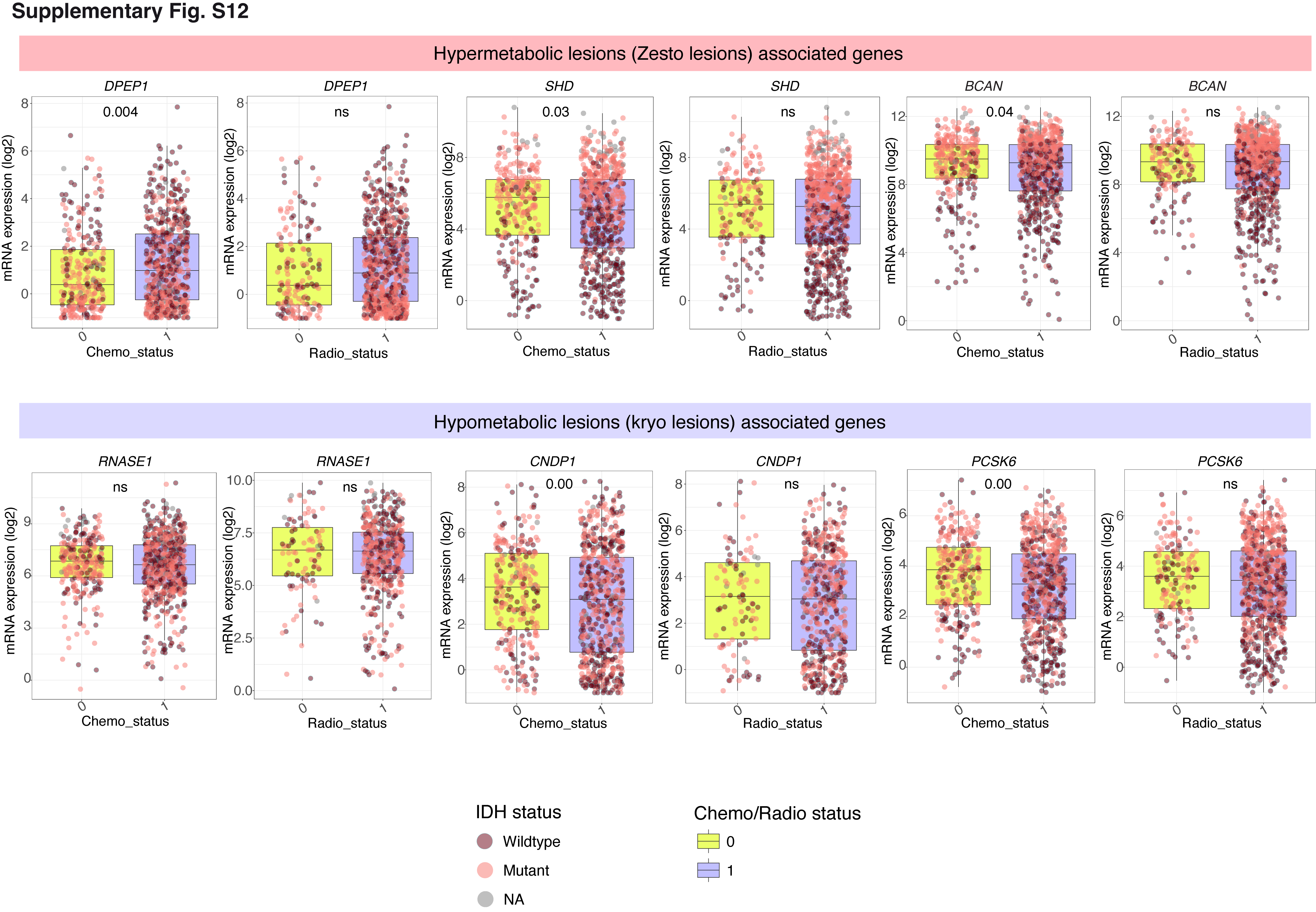

Supplement: noaf071_Supplementary_Tables_S1-S4_Figures_1-S13 [file noaf071_supplementary_tables_s1-s4_figures_1-s13.zip › S12.tif]
